# Natural switches in behaviour rapidly modulate hippocampal coding

**DOI:** 10.1038/s41586-022-05112-2

**Published:** 2022-08-24

**Authors:** Ayelet Sarel, Shaked Palgi, Dan Blum, Johnatan Aljadeff, Liora Las, Nachum Ulanovsky

**Affiliations:** 1 Department of Brain Sciences, Weizmann Institute of Science, Rehovot, Israel; 2 Department of Neurobiology, University of California, San Diego, CA USA

**Keywords:** Spatial memory, Neural encoding, Hippocampus

## Abstract

Throughout their daily lives, animals and humans often switch between different behaviours. However, neuroscience research typically studies the brain while the animal is performing one behavioural task at a time, and little is known about how brain circuits represent switches between different behaviours. Here we tested this question using an ethological setting: two bats flew together in a long 135 m tunnel, and switched between navigation when flying alone (solo) and collision avoidance as they flew past each other (cross-over). Bats increased their echolocation click rate before each cross-over, indicating attention to the other bat^1–9^. Hippocampal CA1 neurons represented the bat’s own position when flying alone (place coding^10–14^). Notably, during cross-overs, neurons switched rapidly to jointly represent the interbat distance by self-position. This neuronal switch was very fast—as fast as 100 ms—which could be revealed owing to the very rapid natural behavioural switch. The neuronal switch correlated with the attention signal, as indexed by echolocation. Interestingly, the different place fields of the same neuron often exhibited very different tuning to interbat distance, creating a complex non-separable coding of position by distance. Theoretical analysis showed that this complex representation yields more efficient coding. Overall, our results suggest that during dynamic natural behaviour, hippocampal neurons can rapidly switch their core computation to represent the relevant behavioural variables, supporting behavioural flexibility.

## Abstract (web summary)

During rapid behavioural switches in flying bats, hippocampal neurons can rapidly switch their core computation to represent the relevant behavioural variables, supporting behavioural flexibility.

## Main

The real world is ever dynamically changing, requiring humans and other animals to rapidly switch between different behavioural modes. For example, when a wild rodent is foraging for food, it occasionally needs to avoid predators and decide towards which burrow to escape, therefore switching dynamically between foraging, predator avoidance and decision-making. However, the neural basis of behaviour is typically studied while the animal is performing one behavioural task at a time, and little is known about how brain circuits rapidly switch between different natural behaviours. Navigation is a complex, dynamic natural behaviour that enables the testing of behavioural switches. It requires the animal to know its own location within the environment, while also paying attention to abrupt events—such as the appearance of unexpected obstacles, predators or conspecifics; the animal may therefore also need to assess the distance to ‘things out there’. The animal’s position is encoded by hippocampal place cells^10–13^; however, this coding has been studied mostly in empty, stationary set-ups that do not imitate the rich dynamism of real-world navigation. There have also been a number of studies that investigated the representation of ‘things out there’ by neurons in the hippocampal formation and surrounding structures^15–23^, but these were all studied under static conditions, without examining dynamic behavioural switches. Here we set out to investigate how brief natural attentional switches to ‘things out there’, which are essential for real-life navigation, affect the representation of space in the hippocampus during navigation. We aimed to test several hypotheses regarding how hippocampal circuits may encode position and distance to ‘things out there’ during dynamic navigation: (1) hippocampal activity always encodes only position; (2) hippocampal activity switches between pure position and pure distance representations; (3) hippocampal activity always multiplexes position and distance information; (4) hippocampal activity switches from a position code to a conjunctive representation of distance by position upon a behavioural need. As we show below, our results are most consistent with hypothesis 4.

## Encoding of distance during brief attentional switches

**Fig. 1:**
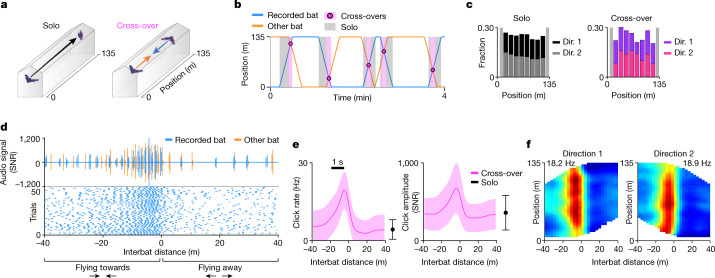
Set-up and behavioural task. **a**, The experimental set-up. Bats flew in pairs and alternated between two behavioural modes: solo (left) and cross-over (right). **b**, Example behaviour (4 min of the full session that is shown in Extended Data Fig. 1a). The blue and orange lines show the positions of the recorded bat and the other bat, respectively; the pink circles show cross-over events; pink rectangles show cross-over flights (±40 m distance around cross-over events); grey rectangles show solo flights. **c**, The distribution of behavioural coverage along the tunnel in an example session: solo (left) and cross-over (right) flight is shown separately for the two flight directions (dir.) (dark and light coloured, stacked). The light grey vertical rectangles show the areas in which cross-overs were not analysed (Methods). **d**, Echolocation example. Top, the audio signal during one cross-over flight for the recorded bat (blue) and for the other bat (orange) as a function of the interbat distance (negative/positive distances: bats flying towards/away from each other). Bottom, raster plot showing the echolocation clicks of the recorded bat (blue dots) for the 50 cross-overs in this session (one flight direction). Note that Egyptian fruit bats emit pairs of clicks^5^. SNR, signal-to-noise ratio. **e**, The population average echolocation click rate (left) and click amplitude (right) for bat 2299 (*n* = 11 sessions) during cross-over flight (data are mean ± s.d. (pink shading), with the s.d. computed over all behavioural data in each distance bin) and solo flight (data are mean ± s.d.). Scale bar, the mean distance flown in 1 s. **f**, 2D click rate maps for interbat distance (*x* axis) by position (*y* axis) for the two flight directions for all sessions of bat 2299, coloured from zero (blue) to peak click rate (red; value indicated). Note that the click rate increased before cross-over, similarly along all of the positions (see the vertical band). Source data

**Extended Data Fig. 1:**
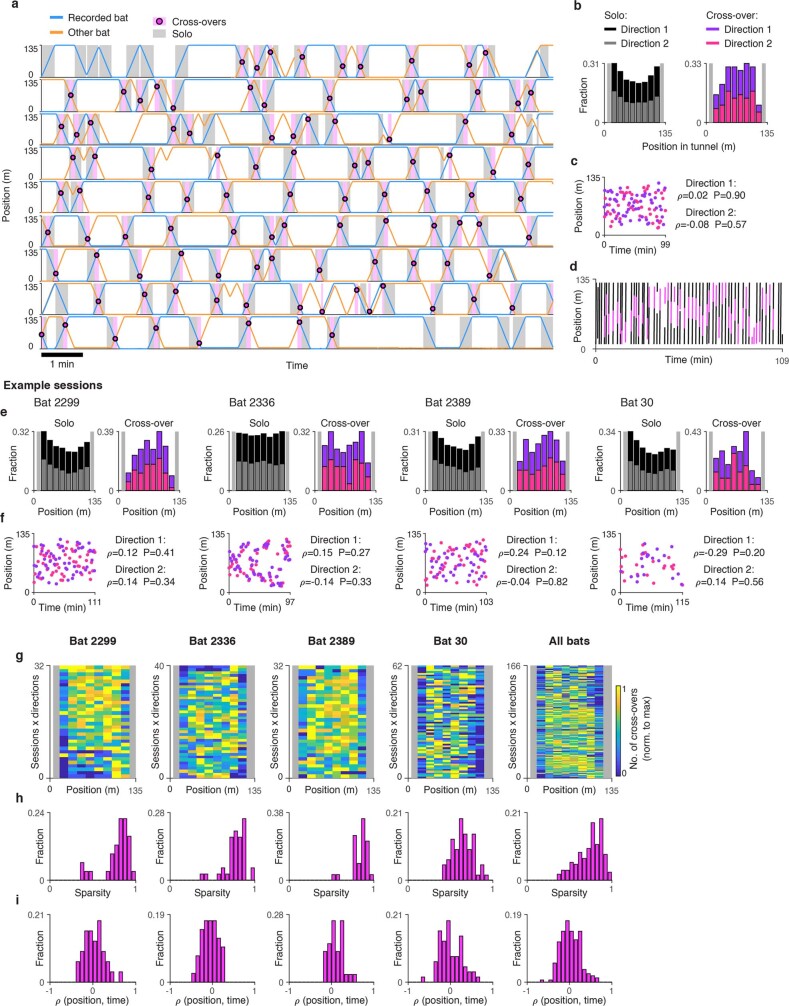
Solo and cross-over behaviours were intermingled and distributed quite uniformly along the tunnel. **a–d**, Example of one experimental session: same experimental session as shown in Fig. 1b. **a**, The positions of the two bats along the entire experimental session. Time in session runs from top-left to bottom-right; scale bar, 1 min. Blue and orange lines: positions of the recorded bat and other bat, respectively. Cross-over events are marked with pink circles, and cross-over flights (window of ±40-m of interbat distance around the cross-over event) are marked with pink rectangles. Solo flights are marked with grey rectangles. **b**, Distribution of behavioural coverage along the tunnel: Solo (left) and cross-overs (right), plotted separately for the two flight-directions (dark and light-coloured, stacked). Light grey vertical rectangles at the edges of both panels: areas where cross-overs were not analysed (Methods); we also note that there were less cross-overs in the bins adjacent to the grey rectangles (see also panels e and g). **c**, Position versus time within the session (time from first to last cross-over), for all the cross-over events in the session shown in a. The cross-over events occurred quite uniformly along the entire tunnel (*y* axis), and were pseudo-randomly distributed over time (*x* axis; direction 1, Spearman correlation of position versus time: ρ = 0.02, *P* = 0.90, *n* = 52 cross-overs; direction 2, Spearman correlation: ρ = −0.08, *P* = 0.57, *n* = 52 cross-overs). **d**, Position versus time within the session for all the solo flights (black) and all the cross-over flights (pink), for one flight-direction, in the same session shown in panels a–c. Note that cross-over flights were intermingled with solo flights, which created “holes” in the solo behaviour, where cross-over flights occurred (see examples of such holes also in the solo rasters in Fig. 2a and Extended Data Fig. 3c). Other reasons for holes in the solo data could be U-turns (which were removed from the analysis) or tracking flights (which were analysed separately in Extended Data Fig. 9). **e–f**, Additional example sessions, one session per bat. For each session the upper panels (e) are plotted as in b, and the lower panels (f) are plotted as in c. Indicated are the Spearman correlations of the cross-over positions versus the time of the cross-overs within the session. **g–i**, Population summaries of behaviour for all the experimental sessions of each bat separately (columns 1–4) and all bats together (column 5). **g**, Distribution of cross-over positions for all sessions × directions (rows) – demonstrating relatively uniform distribution of cross-overs along the tunnel, with no strong behavioural biases (in most sessions). Each row is normalized to its maximal value. **h**, Distribution of sparsity, where the sparsity for each session was computed over the histogram of cross-overs – i.e. sparsity of the rows of the matrix plotted in panel g (${\rm{sparsity}}={\left\langle {r}_{i}\right\rangle }^{2}/\left\langle {r}_{i}^{2}\right\rangle$, where *r*_i_ are the values in each bin in the histogram). Sparsity is bound between 0 and 1, with a value of 1 indicating uniform distribution. Note that for most sessions the sparsity was relatively high (close to 1), indicating nearly-uniform distribution of cross-over events along the tunnel. **i**, Distribution of Spearman correlations between position of cross-overs and time of cross-overs, for all the sessions × directions (examples of these Spearman correlations are shown in panels **c**,**f**). Note that for most sessions there was low correlation between time and the position of the cross-overs – indicating a relatively uniform behaviour along the session, with no systematic trends over time.

**Extended Data Fig. 2:**
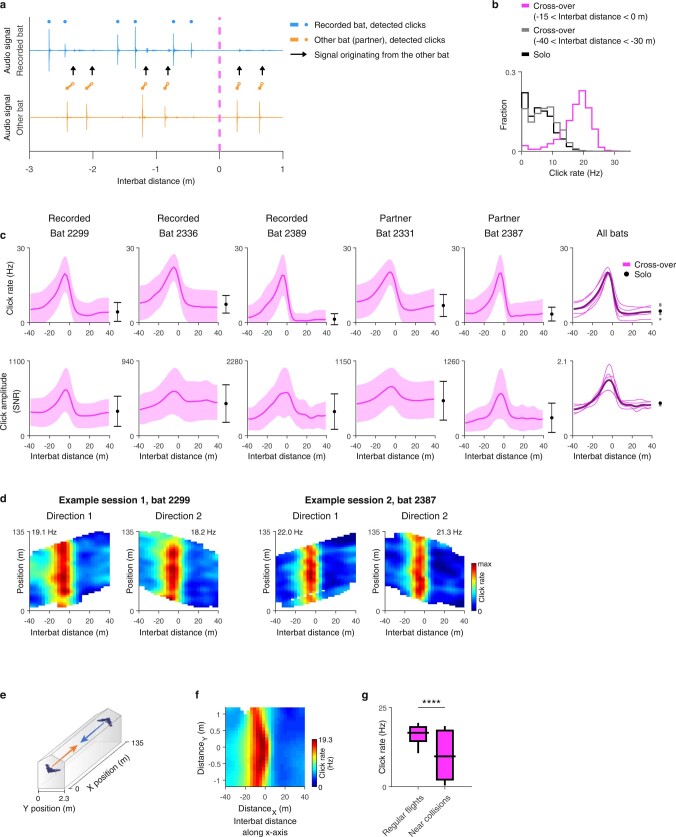
Bats were attentive to the other bat during cross-overs, as indicated by increased echolocation click-rate. **a**, Audio signals (filtered 10–40 kHz) for the recorded bat (blue) and the other bat (orange), at short interbat distances during a cross-over (dashed pink line indicates the cross-over event = distance 0). The two signals were recorded through ultrasonic microphones placed on the head of each bat (Methods). Blue dots mark clicks detected via the microphone on the recorded bat. Bottom filled orange dots mark clicks detected via the microphone on the other bat. Top empty orange dots (shifted in time) mark the time at which the bottom (filled) orange click of the other bat was expected to be recorded through the microphone on the recorded bat, given the distance between the two bats (see also black arrows; this ‘expected time shift’ was computed as the distance between the bats divided by the speed of sound). Note that indeed above each black arrow there is a very small click in the blue audio trace of the recoded bat – which corresponds to clicks produced by the other bat (these small signals were observed only when the bats were very close to one another: ~3 m). Therefore, by excluding such small clicks that appeared around the expected time (as marked here by the black arrows), we could ensure that none of the detected clicks on the recorded bat (blue) has originated from the other bat’s echolocation (orange) – and vice versa (see Methods). **b**, Distribution of click-rates for solo flights (black) and for cross-over flights in two different distance ranges: pink, computed for interbat distance between –15 m to 0 m, and grey, computed for interbat distance between −40 m to −30 m (a large distance, where the bats behaved similarly to solo). Note that the pink distribution is highly separated both from the grey distribution and from the black distribution (Kolmogorov-Smirnov test: *P* < 10^−300^ for both comparisons; Wilcoxon rank sum test: *P* < 10^−300^ for both) – suggesting that the bats were in different behavioural modes during solo versus cross-overs. In particular, note that during solo flights, the bats almost never increased the click-rate as high as during the cross-over encounters. **c**, Population average click-rate (top) and click-amplitude (bottom), during cross-over flights (pink lines) and solo flights (black error bars). Left 5 panels – individual bats: the black error bar and pink shading represent mean ± s.d for solo and cross-over, respectively (bat 2299 [same bat as in Fig. 1e]: *n* = 962 cross-over flights and *n* = 107,418 solo time bins; bat 2336: *n* = 254 cross-over flights and *n* = 26,783 solo time bins; bat 2389: *n* = 108 cross-over flights and *n* = 9,831 solo time bins; bat 2331: *n* = 1,217 cross-over flights and *n* = 116,737 solo time bins; bat 2387: *n* = 106 cross-over flights and *n* = 10,529 solo time bins; the time bins for solo flight were adjusted to match the bins in cross-over tuning; bat 30 was not analysed here because its audio was recorded using a different audio-logger device and therefore its click-amplitude was not comparable with the other bats). Rightmost panel – population average click-rate (top) and amplitude (bottom; *n* = 5 bats; each bat was normalized here to the mean amplitude during cross-over): shown are the averages for individual bats during cross-overs (pink curves) and grand-average over all bats (purple curve) – and the averages during solo (dots; individual bats are shown by the grey dots, population average is the black dot). **d**, 2D click-rate map plotted as a function of the interbat distance (*x*-axis) and position (*y*-axis) – shown for two example sessions from two different bats (see population of bat 2299 in Fig. 1f). Colour-coded from zero (blue) to maximal click-rate (red; value indicated): see colour-bar. Note that the click-rate increased at approximately −20 metres before cross-over, along *all* the positions in the tunnel (vertical red band). **e**, Schematic of the tunnel, showing the tunnel’s X coordinate (long axis) and Y coordinate (lateral axis); the accuracy of the Z measurement was lower than X,Y, hence we did not analyse it. **f**, Echolocation click-rate map (pooled over all bats) plotted for *lateral* interbat distance in the Y-axis of the tunnel (Distance_Y_ = the lateral offset between the two bats) versus the interbat distance in the X-axis of the tunnel (Distance_X_). Note that the click-rate was higher when the distance between the bats was low in both axes – i.e. when the bats were on a tight collision-course (notice the red hotspot at (0,0) in this map). **g**, Median click-rate per-flight for regular cross-over flights (left, *n* = 948 flights: all the regular flights of bat 2299), compared to cross-over flights where the bats nearly collided (right, *n* = 14 flights). These actual near-collisions were identified as flights in which at short Distance_X_ between the bats (less than ±0.25 m) they also flew-by at short Distance_Y_ (less than ±0.25 m), and reduced their speed within this distance window to < 4 m/s (a highly-unusual slowing-down, akin to a “push on the brakes”). In those rare near-collision flights, the bats exhibited significantly lower click-rate (Permutation test on the difference between the means of the two groups: *P* = 0.0001; Wilcoxon rank sum test: *P* = 0.006), and sometimes they did not echolocate at all – suggesting that lapse of attention (low click-rate or absence of clicks) leads to near-collisions or full collisions. Note that since these “braking events” were such rare events (*n*=14), their contribution to the heat-map in panel f was small and was averaged out. Box-and-whisker plots show the median (horizontal line), 25–75% range (box) and 10–90% range (whiskers).

We trained pairs of Egyptian fruit bats to fly together in a long 135 m linear tunnel between two landing balls, where food was given. The bats alternated between two behavioural modes (Methods): (1) solo: only one bat flew alone, or was >40 m away from the other bat (Fig. 1a (left)); or (2) cross-over: the two bats flew towards each other from opposite directions at <40 m (Fig. 1a (right)). The bats took off from the balls non-synchronously at random timing relative to each other, creating intermingled solo and cross-over flights, which were distributed approximately uniformly along the tunnel (Fig. 1b,c and Extended Data Fig. 1). During cross-overs, the bats bypassed each other at a very high relative speed of around 14 m s^−1^ (the sum of both bats’ speeds; the speeds of individual bats are shown in Extended Data Table 1) and, therefore, had to be attentive to avoid collision between one another. To measure the bats’ attention, we recorded their echolocation clicks (sonar signals), because many bat species have been shown to increase echolocation click rate when attention is needed; thus, echolocation provides an index of the bat’s moment-to-moment attention^1–9^. Indeed, we found that, during cross-overs, the bats increased their echolocation click rate by around fourfold and click amplitude by around twofold (Fig. 1d,e and Extended Data Fig. 2c), with this echolocation profile being uniform along the entire tunnel (Fig. 1f and Extended Data Fig. 2d). In all of the bats, the increase was rapid (about 1 s), and constituted a switch between two distinct behavioural phases (Extended Data Fig. 2b). This increase in click rate suggests that the bats were highly attentive during these demanding cross-over flights. In the rare cases of near collisions, the bats exhibited fewer echolocation clicks (Extended Data Fig. 2e–g), suggesting that a low click rate indicates a lapse of attention, which may lead to collisions. This provides further support for the link between echolocation rate and attention.

**Extended Data Fig. 3:**
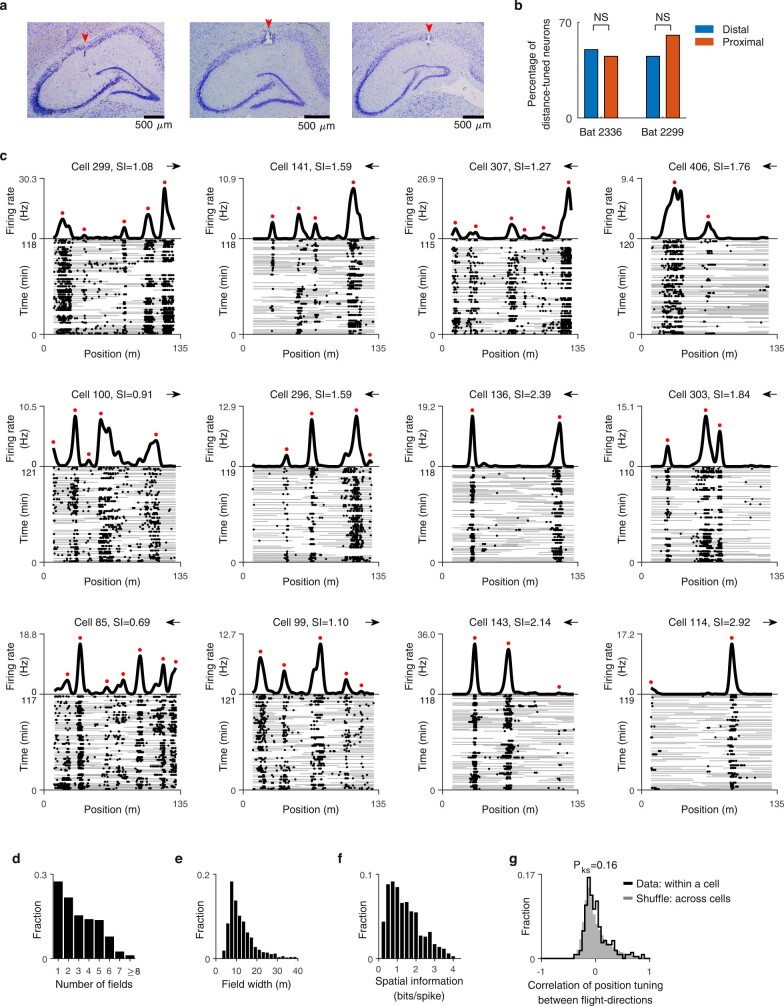
Histology, and place cells in the large-scale linear track during solo flights. **a**, Examples of coronal sections through dorsal hippocampus of two of our recorded bats (left, bat 2336; middle and right, bat 2299). Red arrowheads mark holes due to a tetrode-track (left), or electrolytic lesions in CA1 at the end of tetrode-tracks (middle and right: two different tetrodes). These examples illustrate the large proximo-distal anatomical span of our recording-sites in dorsal CA1. Scale bars, 500 µm. Similar reconstruction of tetrode-tracks was performed for all the tetrodes in all the 4 bats. **b**, Percentage of pyramidal distance-modulated neurons (defined either in 1D or 2D, see Fig. 3g, thick black line) in proximal tetrodes (red) versus distal tetrodes (blue), for bats 2336 and 2299 (the two bats for which we had large proximo-distal spread of the tetrodes). For each bat we separated the tetrodes into the proximal tetrodes versus the distal tetrodes of the tetrode-bundle (excluding ambiguously-located tetrodes in the centre of the bundle). There was no relation between the proximal/distal location of the tetrode in CA1 and the percentage of distance-tuned neurons (comparing proximal versus distal: bat 2336: log odds ratio test: *P* = 0.654, Fisher’s exact test: *P* = 0.673, *n*_proximal_ = 31 cells, *n*_distal_ = 70 cells; bat 2299: log odds ratio test: *P* = 0.102, Fisher’s exact test: *P* = 0.116, *n*_proximal_ = 114 cells, *n*_distal_ = 62 cells; FDR-corrected for multiple comparisons for 2 bats). **c**, Examples of 12 place cells, during solo flights. Top, position tuning-curves (firing rate maps). Significant place fields are marked by red dots above the peak; arrows indicate flight direction. Bottom, spike rasters: raw positional data (grey) with spikes overlaid (black dots). Spatial information (SI, in bits/spike) is indicated above each cell. **d**, Distribution of the number of fields per direction for all the significant place cells (*n* = 613 cells × directions). **e**, Distribution of place-field sizes for all the significant place cells (*n* = 1,856 place fields). **f**, Distribution of spatial information for all the place cells (*n* = 613 cells × directions). **g**, Distribution of Pearson correlations between position tuning-curves for the two flight-directions of the same cell (black line) or between the two directions across different cells (grey bars: cell shuffling; Kolmogorov-Smirnov test of data versus shuffles: *P* = 0.16; *n* = 251 cells in the black distribution – computed only for cells where both directions were significantly place-tuned). Overall, our data for place fields in this large-scale environment is similar to what we found before^14^: we observed here multiple place fields per neuron, with a variety of spatial scales per neuron, i.e. different fields of the same neuron exhibited highly-varying sizes (multiscale code). We note that there were some small numerical differences in place-field numbers and place-field sizes, as compared to ref. ^14^, because: (i) we used here a shorter portion of the tunnel (135 m) than in our previous work (200 m)^14^; and (ii) we modified here the parameters used to compute the position tuning-curves (we used a larger bin size and larger smoothing-kernel, since we had here less solo data than in ref. ^14^).

The use of a very large environment enabled us to examine these fast behavioural switches, because (1) bats fly very fast in large environments, allowing for very rapid switches; (2) the large space allowed for a substantial baseline before and after the cross-overs; and (3) it allowed the bats to perform cross-overs at multiple positions, therefore enabling us to disentangle distance from position. We used a wireless electrophysiology device to record the activity of 430 neurons from dorsal hippocampal area CA1 of four bats during flight (Extended Data Fig. 3a,b and Extended Data Table 1; 389 putative pyramidal cells, 41 putative interneurons; we analysed the data separately for the two flight directions: 693 valid pyramidal cells × directions, 74 valid interneurons × directions; Methods). On the basis of the solo flights, we classified 88.5% of the pyramidal cells as significant place cells (*n* = 613 cells × directions exhibited significant positional modulation of firing rate). Place cells exhibited multiple place fields in this long track (mean ± s.d., 3.03 ± 1.81 place fields per direction), with variable field sizes (Extended Data Fig. 3c–g), consistent with our previous report^14^.

**Fig. 2:**
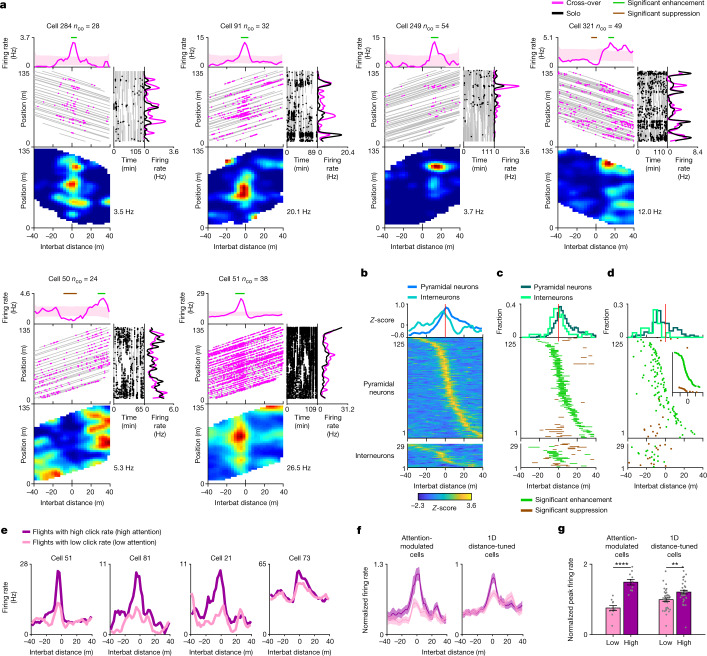
Hippocampal CA1 neurons represent the distance to another bat upon brief attentional switches during navigation. **a**, Examples of 1D distance neurons. *n*_co_ represents the number of cross-overs. For each cell, the top row shows the tuning curve for 1D distance (pink line) and shuffles (shading). Horizontal lines denote significant enhancement (green) or suppression (brown). In the middle row, the left plot shows the recorded bat position (*y* axis) and interbat distance (*x* axis) during cross-overs (grey; the two flight directions yield positive versus negative slopes of the grey lines), with spikes overlaid (pink dots); the centre plot shows the spike raster during solo flights (grey, behaviour; black dots, spikes), showing position (*y* axis) versus time in the session (*x* axis; the solo raster has holes in which cross-overs occurred; Extended Data Figs. 1d and 3c); and the right plot shows place tuning during solo flights (black) and during cross-over flights (pink). The bottom row shows the 2D firing-rate map of position (*y* axis) by interbat distance (*x* axis), coloured from zero (blue) to peak firing rate (red; value indicated). **b**–**d**, Population summary of all significant 1D distance neurons for putative pyramidal neurons (*n* = 125) and interneurons (*n* = 29). **b**, Top, the mean of *z*-scored distance tuning curves. A raster of *z*-scored tuning curves is shown separately for pyramidal neurons (middle) and interneurons (bottom), sorted by peak distance. **c**, Top, the fraction of cells with significant distance enhancement, as a function of interbat distance. Significantly enhanced and suppressed bins for pyramidal neurons (middle) and interneurons (bottom) are shown. **d**, Top, the distribution of enhancement response onset (the distance at which the tuning crossed 90% of the shuffle distribution). The response onset for pyramidal neurons (middle) and interneurons (bottom) is shown. Inset, pyramidal cells sorted by onset distance (separately for enhancement and suppression). **e**–**g**, Attentional modulation of distance tuning, comparing higher-click-rate flights (purple, high attention) with lower-click-rate flights (pink, low attention). **e**, Example cells: 1D distance tuning curves. **f**, The average population tuning curves (each cell was normalized to its peak firing rate computed using all flights) for all 1D distance neurons (right) and all cells significantly modulated by click rate (left). **g**, The peak firing rate (normalized as described in **f**), for high- and low-click-rate flights (purple and pink), plotted for 1D distance neurons (right; one-tailed *t*-test: *t* = 2.47, *P* = 0.009*, n* = 37 cells × directions), and neurons that were significantly modulated by click rate (left; one-tailed *t*-test: *t* = 6.44, *P* = 3.72 × 10^−5^, *n* = 11). For **f** and **g**, data are mean ± s.e.m. Source data

**Extended Data Fig. 4:**
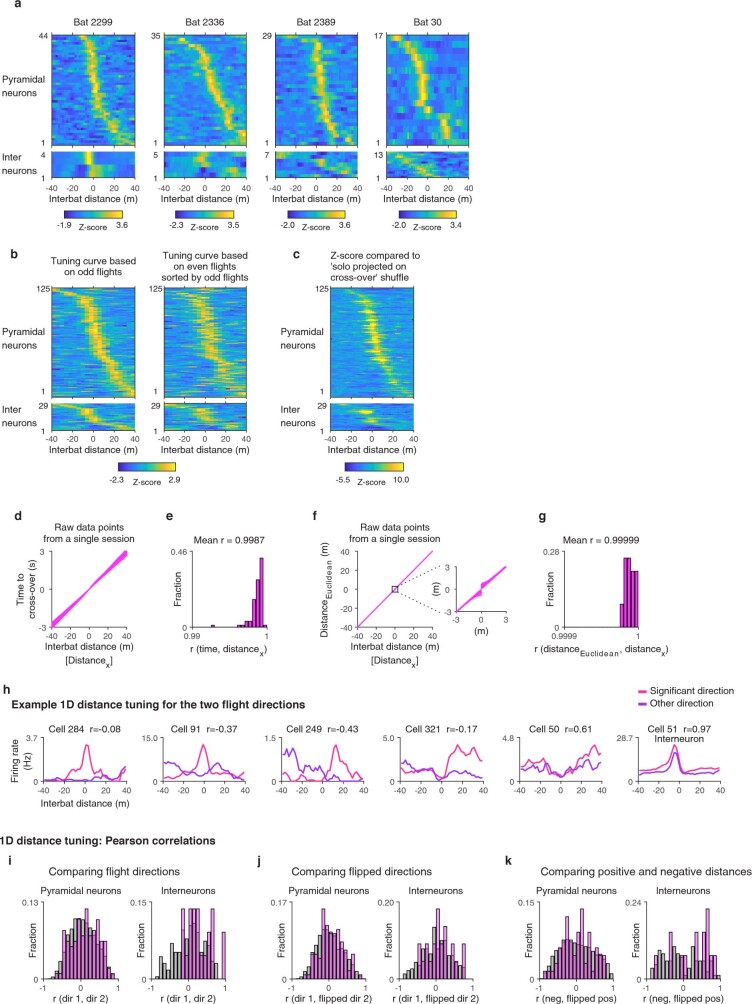
Tuning to 1D interbat distance. **a**, Population summary for individual bats (plotted similar to Fig. 2b): *z*-scored tuning-curves plotted separately for pyramidal neurons (top) and interneurons (bottom), sorted by preferred distance (the distance of the peak of the distance-tuning). **b**, Stability of the 1D distance tuning: shown are *z*-scored tuning-curves that were constructed based on odd flights (left) or even flights (right), separately for pyramidal neurons (top; *n* = 125) and interneurons (bottom; *n* = 29); sorted by the preferred distance (peak of the distance-tuning) in the odd-flights data (i.e., sorted according to the left panel). Note that the tuning was stable and was preserved in even flights versus odd flights. **c**, *Z*-scored distance tuning curves, where the *z*-scoring was done relative to the solo-projected on cross-over shuffle (see Extended Data Fig. 5) for pyramidal neurons (top) and interneurons (bottom); sorted by the preferred distance (peak of the distance-tuning). Colour limits were set from the minimum across all tuning curves to *z* = 10; the most strongly-responding neuron had *z* = 36.5, and the 90% percentile across the population was *z* = 15.7 – signifying very strong and significant distance responses. **d–e**, Interbat distance and time to cross-over were highly correlated. **d**, Raw data from a single session: time to cross-over (*y*-axis) versus interbat distance (*x*-axis). **e**, Distribution of Pearson correlations between time to cross-over and interbat distance, for all the recording sessions (*n* = 83 sessions). Note that the range of correlations shown is from 0.99 to 1; mean Pearson correlation is 0.9987. Since both variables (time and distance) are highly correlated, we could not distinguish between time coding versus distance coding, and decided to perform all analyses as a function of distance (except Fig. 3c, and Extended Data Figs. 6k, 7d, 11e). **f–g**, Interbat distance along the long axis of the tunnel (Distance_x_) and the Euclidean distance between the bats (Distance_Euclidean_), which takes into account also the lateral axis of the tunnel (Y-axis in Extended Data Fig. 2e). **f**, Raw data from a single session (same session as in d): Euclidean distance (*y*-axis) versus Distance_x_ (*x*-axis). These two distance-measures were highly correlated. Inset, zoom-in on ±3m on both axes: There is high correlation in this range, too. Note that the region around 0, in which there is a slight difference between the Euclidean distance and Distance_x_, is a very small region – and falls within one bin of our neuronal interbat distance analysis; therefore, plotting neuronal tuning curves using the Euclidean distance would yield virtually identical results to plotting them using Distance_x_ as done in the paper. **g**, Distribution of Pearson correlations between the Euclidean distance and Distance_x_ for all the recorded sessions (*n* = 83 sessions). Note that the range of correlations shown is from 0.9999 to 1; mean Pearson correlation is 0.99999. Since both variables (Distance_x_ and Euclidean distance) are highly correlated, we decided to focus in this study on Distance_x_ (distance along the tunnel) – to be consistent with the 1D position coding, which was also measured along the tunnel. **h–k**, Comparisons of 1D distance tuning across flight-directions and across positive/negative distances. Here we tested whether our neurons are tuned to absolute interbat distance in a similar manner regardless of flight-direction (flying east versus west) and regardless of the relative direction between the bats (flying towards or away from one another – we note that bats can sense the presence of another bat via their echolocation also behind them, as echolocation signals spread also backwards^5,27^). We expect that a ‘pure distance cell’ with a preferred distance of 20 m, for example, would fire symmetrically at two distances: ±20 m (i.e. would have a double peak in its distance tuning curve, at −20 m and at +20 m); and we also expect to find the same tuning in both flight directions (east and west) – i.e. overall we expect a 4-fold symmetry. Alternatively, if the response of the neurons is not purely to distance, it suggests that there is a directional component to the tuning, which is similar to a vectorial signal. **h**, Examples of 1D distance neurons (same neurons as in Fig. 2a), showing the 1D distance tuning in the significant direction (pink; plotted also in Fig. 2a) and in the other flight direction (blue-purple). Pearson correlations of the distance tuning curves between the two flight-directions are indicated. **i–k**, Population: Various comparisons of Pearson correlations between the 1D distance tuning-curve of a cell versus the other direction or versus flipped direction (pink); for control shown are the same comparisons across different cells (grey: cell shuffling). **i**, Comparing distance tuning in the two different flight directions (flying east versus west). Left: pyramidal cells (Kolmogorov-Smirnov test of data versus shuffles: *P* = 0.09, Wilcoxon rank sum test: *P* = 0.08; *n* = 108 pyramidal cells that had at least one direction with significant distance modulation). Right: interneurons (Kolmogorov-Smirnov test: *P* = 0.04, Wilcoxon rank sum test: *P* = 0.003; *n* = 22 interneurons that had at least one direction with significant distance modulation). The higher correlations in interneurons suggest that interneurons are more likely to be direction-invariant distance cells, while pyramidal neurons fire differently depending on flight direction – as would be expected from a vectorial representation. Notably, when we limited this analysis to include only neurons that had significant distance-tuning in both flight directions, we found that those cells had a higher correlation values between the distance-tuning of both flight directions, as compared to the shuffle correlations across cells (Pyramidal neurons: Kolmogorov-Smirnov test of data versus shuffles: *P* = 0.01, Wilcoxon rank sum test: *P* = 0.01, *n* = 17 cells; Interneurons: Kolmogorov-Smirnov test of data versus shuffles: *P* = 0.01, Wilcoxon rank sum test: *P* = 2.49 × 10^−3^, *n* = 7 cells) – indicating that some neurons do encode distance invariant of flight direction. We note that the probability to exhibit significant distance tuning in both flight directions (P_observed distance-tuning both directions_) was higher than expected from the null hypothesis of independence (P_null distance-tuning both directions_ = P_distance-tuning dir 1_ × P_distance-tuning dir 2_) – both for pyramidal cells (binomial test: *P* = 0.023) and for interneurons (binomial test: *P* = 7.81 × 10^−5^): This suggests that CA1 neurons have a “propensity” to exhibit distance tuning, which is expressed in both flight-directions. **j**, Comparing Pearson correlations between the distance-tuning in flight direction 1 and the flipped distance-tuning in flight direction 2. Left: pyramidal cells (Kolmogorov-Smirnov test of data versus shuffles: *P* = 0.13, Wilcoxon rank sum test: *P* = 0.17; *n* = 108 pyramidal cells that had at least one direction with significant distance modulation). Right: interneurons (Kolmogorov-Smirnov test: *P* = 0.84, Wilcoxon rank sum test: *P* = 0.44; *n* = 22 interneurons that had at least one direction with significant distance modulation). **k**, Comparing Pearson correlation computed between negative distances and flipped positive distances (mirror image around zero-distance; this was aimed to test tuning symmetry when bats are flying toward each other compared with flying away). Computed only for neurons that did not have a significant bin at zero distance (because such cases would exhibit by definition high correlations due to high mirror-symmetry). Left: pyramidal cells (Kolmogorov-Smirnov test of data versus shuffles: *P* = 0.65, Wilcoxon rank sum test: *P* = 1.00; *n* = 64 pyramidal cells). Right: interneurons (Kolmogorov-Smirnov test: *P* = 0.02, Wilcoxon rank sum test: *P* = 0.02; *n* = 14 interneurons). Overall, the data in this figure suggest that the majority of distance-tuned neurons exhibited different distance-tuning for the two flight directions (east versus west), and different distance-tuning for the two relative directions between the bats (other bat in front or behind – i.e. flying towards or away). This suggests that our results reflect vectorial signals that have both a distance and a direction component. Interestingly, the bat’s distance-tuned cells showed a mixture of enhanced and suppressed responses – akin to the behaviour of other types of vectorial cells in the hippocampal formation and surrounding regions, such as boundary-vector cells^18,66–69^. However, it is noteworthy that some of our cells did show higher correlations between flight directions (this was true for pyramidal cells with significant distance-tuning in both flight direction, and also for interneurons) – and thus some of these neurons could be regarded as direction-invariant distance-coding neurons.

**Extended Data Fig. 5:**
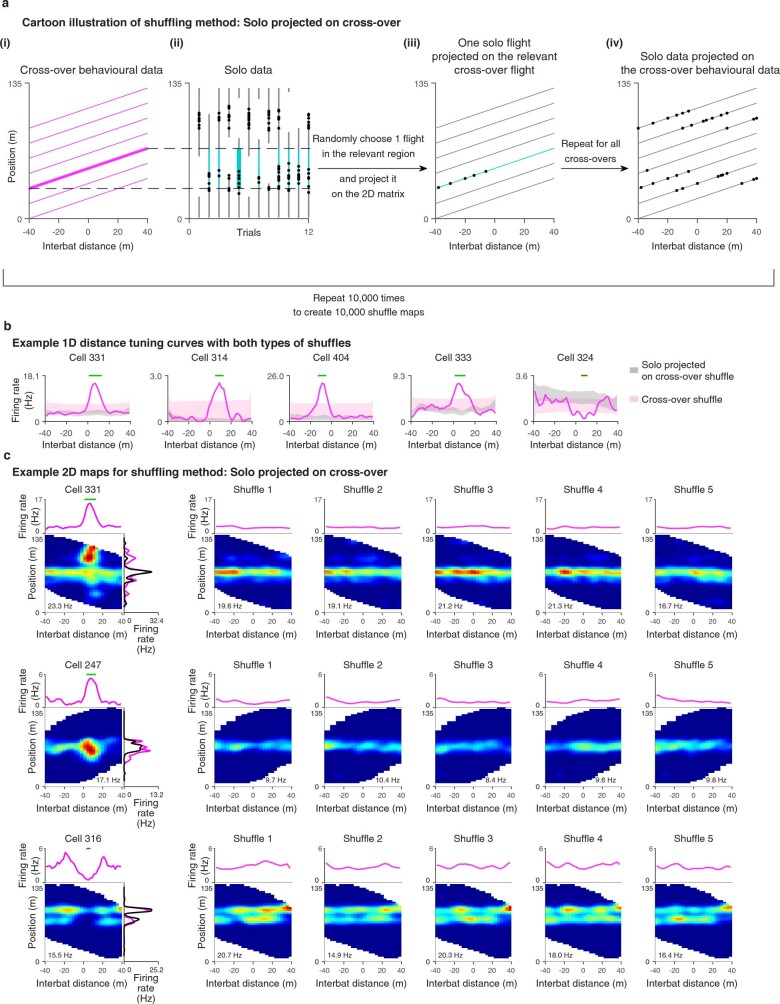
Shuffling method ‘solo projected on cross-over’. **a**, Cartoon illustrating the “solo projected on cross-over” shuffling method (one of the two shuffling methods used throughout this study; see Methods). This shuffling-method is aimed to compare the actual firing during cross-overs to the expected firing based on the solo data – in order to account for the prominent position-coding in the hippocampus. For each cross-over flight (panel a (i)) we looked for all the solo flights that occurred within the same position-range as the cross-over flight (panel a (ii); all turquoise-coloured solo flight epochs covered fully the same position range as the thick pink cross-over flight in panel a (i)). Then we randomly chose 1 of these solo flights (panel a (ii): thick turquoise line), and linearly projected the spikes that occurred during this solo flight onto the cross-over flight data, to yield the projected interbat distance of each spike (panel a (iii); black dots on top of the turquoise flight). We repeated this process for all the cross-over flights, to create a full matrix that is based on combining behaviour taken from the cross-over data and neuronal responses taken from the solo data (panel a (iv)). This entire process (all four panels i–iv) was repeated 10,000 times, to create 10,000 shuffle matrices, each combining the cross-over behaviour with the cell’s position solo tuning and spiking-statistics – but *without* any explicit distance modulation. These 10,000 shuffle matrices (shuffle maps) served as a null hypothesis for how the cell should fire assuming it has no distance tuning. **b**, Examples of significant 1D distance cells. Shown for each cell: the 1D distance tuning-curve (pink line), tuning-curves of the ‘solo projected on cross-over’ shuffles (shaded grey – the shuffles from a), and tuning-curves of the rigid cross-over shuffles (shaded pink – this is the second type of shuffle that we used in this study: see Methods). Horizontal green lines denote significant enhancement bins, and horizontal brown line denotes significant suppression bins: significance was computed based on *both* types of shuffles (Methods; we note that for enhancement-tuning, the cross-over shuffles were above the other type of shuffle, and vice versa for the suppression-tuning – because the cross-over shuffle preserves the number of spikes and thus reflects the average of the pink tuning-curve, which goes up in enhancement and down in suppression). **c**, Examples of 2D distance-by-position firing maps of ‘solo projected on cross-over’ shuffles from three cells. Leftmost column – the cell’s actual experimental data during cross-over: Top left, tuning-curve for 1D distance; horizontal green/brown lines denote bins with significant enhancement/suppression. Bottom left (main panel): firing rate map of position (*y*-axis) by interbat distance (*x*-axis). Bottom right: position place tuning of the cell during solo flights (black) and during cross-over flights (pink). Five rightmost columns – examples of five random shuffle maps for each neuron: bottom, 2D firing rate map of position (*y*-axis) by interbat distance (*x*-axis); top, the corresponding 1D distance tuning curve of the shuffled data. The Y limits and colour-scale were held fixed for each cell, i.e. for all the panels of the same row (2D maps coloured from zero [blue] to the maximum value over all panels [red; value indicated for each map]). Note that the 2D firing rate maps of the shuffles show horizontal stripes, representing the place fields, and there is no real distance modulation in these maps: the small modulations in the firing rate seen along the distance axis in these shuffles are caused by the somewhat non-uniform behavioural coverage and by the random spiking statistics. These two factors exist also in the cross-over data – and therefore these matrices serve as a ‘null hypothesis’ that controls both for behavioural coverage and for spiking statistics, reflecting how the cell would fire if it was *not* truly modulated by distance.

**Extended Data Fig. 6:**
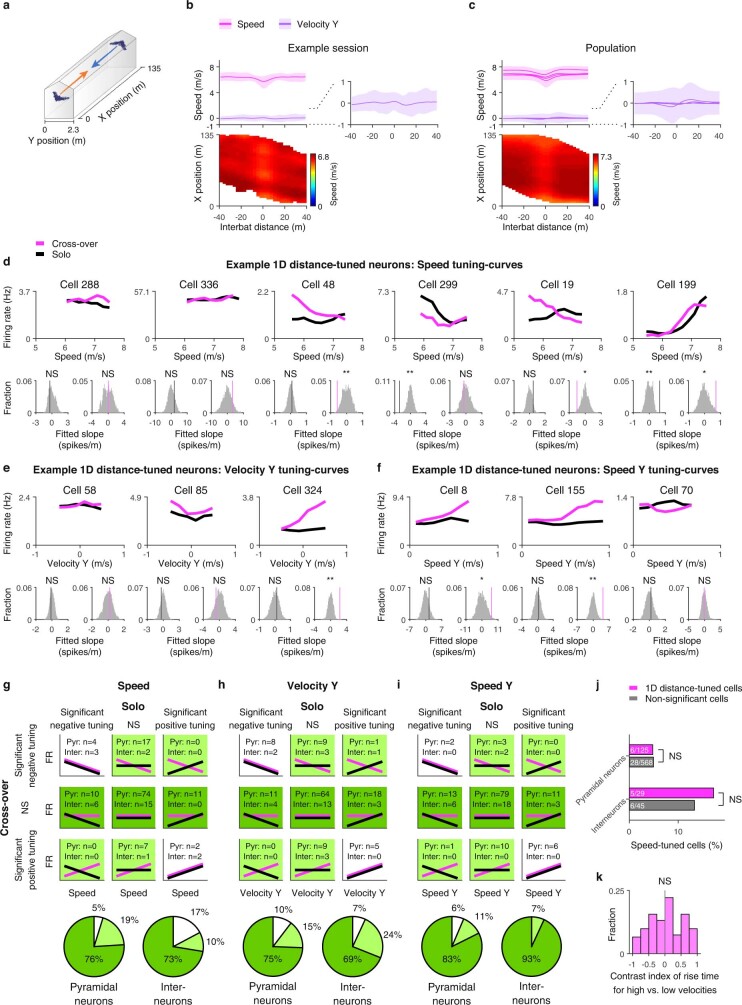
The 1D distance tuning could not be explained by changes in movement variables during cross-overs. **a**, Rationale of analysis. Shown is a schematic of the tunnel, depicting the tunnel’s X coordinate (long axis) and Y coordinate (lateral axis); the accuracy of the Z measurement was lower than X,Y, hence we did not analyse it. In order to test whether the neuronal modulations found during cross-overs reflect genuine distance-tuning, which could not be explained by motor variables, we reasoned as follows: If a neuron is modulated by motor variables, then we expect to see the *same* movement-related modulation both during solo and during cross-over flights. We considered three types of movement variables: (i) Speed – the speed computed over both axes of the tunnel (X and Y together), where we aimed to control for changes in speed, such as slowing down. (ii) Velocity Y – velocity just in the Y axis of the tunnel (lateral), aiming to control for lateral movements of the bat towards the side of the tunnel during cross-overs. (iii) Speed Y – the absolute velocity in the Y axis (here lateral movement to the left or to the right are considered to be the same). **b–c**, Profiles of the movement variables during cross-overs for an example session (**b**) and population pooled over all the sessions of all bats (*n* = 166 sessions × directions) (**c**). Top: mean speed (pink) and velocity Y (purple); shaded colours show the 5–95% percentile range. The lines in panel c-top represent individual means for each bat. Inset, zoom on velocity Y (purple) for the relevant velocity range. Bottom: 2D speed maps as a function of interbat distance and position, for one flight direction; position here denotes the position along the X-axis of the tunnel (X position); maps are colour-coded from zero (blue) to maximal speed (red; see colour-bar). Note that speed-changes were rather small (very small colour-changes), and were distributed quite uniformly along the tunnel (see vertical red band in c). **d–f**, Examples of tuning-curves to movement variables for 1D distance cells: Tuning to Speed (**d**), Velocity Y (**e**) and Speed Y (**f**). For each cell: top, tuning curves for movement variables during solo (black) and during cross-over (pink). Bottom, linear slope fitted to the tuning-curve during solo (left; black line) and to the tuning-curve during cross-over (right; pink line), together with the slopes fitted to the shuffle tuning-curves (grey histograms, 10,000 shuffles). ‘*’ denotes significant tuning with *P* < 0.05, ‘**’ with *P* < 0.01; ‘NS’, non-significant. Exact *P*-values: Cell 288: *P*_*solo*_ = 0.30, *P*_*cross-over*_ = 0.46; Cell 336: *P*_*solo*_ = 0.20, *P*_*cross-over*_ = 0.12; Cell 48: *P*_*solo*_ = 0.30, *P*_*cross-over*_ = 0.009; Cell 299: *P*_*solo*_ = 0.003, *P*_*cross-over*_ = 0.33; Cell 19: *P*_*solo*_ = 0.07, *P*_*cross-over*_ = 0.01; Cell 199: *P*_*solo*_ = 0.003, *P*_*cross-over*_ = 0.01; Cell 58: *P*_*solo*_ = 0.32, *P*_*cross-over*_ = 0.37; Cell 85: *P*_*solo*_ = 0.23, *P*_*cross-over*_ = 0.22; Cell 324: *P*_*solo*_ = 0.34, *P*_*cross-over*_ = 0.003; Cell 8: *P*_*solo*_ = 0.16, *P*_*cross-over*_ = 0.02; Cell 155: *P*_*solo*_ = 0.38, *P*_*cross-over*_ = 0.003; Cell 70: *P*_*solo*_ = 0.33, *P*_*cross-over*_ = 0.47. **g-i**, Top: tables showing the number of 1D distance cells in each of the 3×3 possible combinations of speed modulations: positive/NS/negative modulation by speed or velocity during cross-over × positive/NS/negative modulation during solo. The numbers are shown for pyramidal neurons (*n*=125) and interneurons (*n*=29) – separately for speed (**g**), velocity Y (**h**) and speed Y (**i**). The schematic line-graphs in pink and black denote the combination of tunings that represent each rubric. Green background represents modulation by speed that could *not* explain the neurons’ distance tuning: specifically, this occurs in cases in which the speed modulation during cross-over was not significant (dark green; examples in d-f that correspond to this case: cells 288, 336, 299, 58, 85, 70), or in cases where the speed modulation during cross-over was significant but was different than in solo (light green; examples in d-f: cells 48, 19, 324, 8, 155) – suggesting that these cells do *not* genuinely encode this motor variable. White background represents cells whose motor modulation might potentially explain the distance tuning: These are cases in which the speed modulation was significant and had the same slope-direction during solo and during cross-over (as in cell 199 in panel d). Bottom, summary of the fraction of cells in each one of the three categories (three colours) taken from the table above. Overall, the majority of the distance modulation of 1D distance cells could *not* be explained by motor variables – as indicated by the high percentage of cells in the two types of green areas of the pie-charts below. **j**, Percentages of speed-tuned neurons (as defined above) for 1D distance cells (pink; 125 pyramidal neurons, top, and 29 interneurons, bottom) and cells that were not significantly tuned to 1D distance (grey; 568 pyramidal neurons, top, and 45 interneurons, bottom). Note that there was no relation between the tendency of cells to exhibit significant 1D distance tuning, and their tendency to exhibit significant speed tuning (i.e., no difference in percentage of speed-tuned cells between the pink and grey bars). In other words, 1D distance-tuned cells were not more likely to be modulated by speed as compared with the rest of the population (χ^2^ test for pyramidal cells, *P* = 0.95; χ^2^ test for interneurons, *P* = 0.64). This again argues against the possibility that speed tuning can underlie the observed 1D distance tuning. **k**, Flight velocity does not affect the rise-time of the neuronal responses during cross-over – supporting neuronal switch rather than multiplexing. Shown is the distribution of contrast indices of the rise-time slope of the neuronal tuning for high versus low flight velocities: (rise time at faster flights – rise time at slower flights) / (rise time at faster flights + rise time at slower flights). Here we tested whether the 2D distance-by-position tuning reflects static multiplexing of the two variables (hypothesis 3 in the main text). If the representation is static, then we expect that the rise-time of the response will depend on flight velocity; specifically, for higher flight velocities the cell would exhibit a steeper slope (shorter rise-time) when computing the time-to-crossover tuning. To this end, we defined the rise-time window as before, namely the time from the median of the shuffle to the first significant bin. We then divided the cross-over flights into two equally-sized groups, according to the flight velocity within the rise-time window (median bisection of velocity; we used here the combined velocity of the two bats, because this is the effective velocity at which the bats move along the distance axis). This yielded two sets of flights: flights with high velocity versus flights with low velocity in the relevant distances for each cell. We then computed the two tuning-curves as a function of time-to-cross-over separately for these two sets of flights. We computed the slope of these two tuning curves at the same rise-time window, and calculated the contrast index between the two slopes – and the histogram of these contrast indices is plotted here. As mentioned above, for a static tuning, the high-velocity tuning curves should yield higher slopes. However, we found that the contrast index of slopes for high velocity versus low velocity was not significantly different from zero (*t*-test: *t* = 0.84, *P* = 0.41; Wilcoxon sign rank test: *P* = 0.43; we included here all the *n* = 120 neurons with 1D distance tuning that had more than 10 cross-over flights and more than 30 spikes for each of the two flight groups [low-velocity and high-velocity]). Further, there was no correlation between the difference in velocities (quantified via the contrast index between the flight velocities) and the difference in the tuning slopes (contrast index of slopes) (the Pearson correlation between the two was: *r* = −0.11, *P* = 0.25; Spearman correlation: *r* = −0.13, *P* = 0.15; *n* = 120). Since we found here that there was no relation between the flight velocity and the rise time, this argues against the hypothesis of a multiplexed code. By contrast, for a neuronal switch (hypothesis 4 in the main text) we expected that the switch-time will have a fixed duration, irrespective of velocity – as we found here. Thus, these results are more consistent with a neuronal switch than with multiplexing.

**Extended Data Fig. 7:**
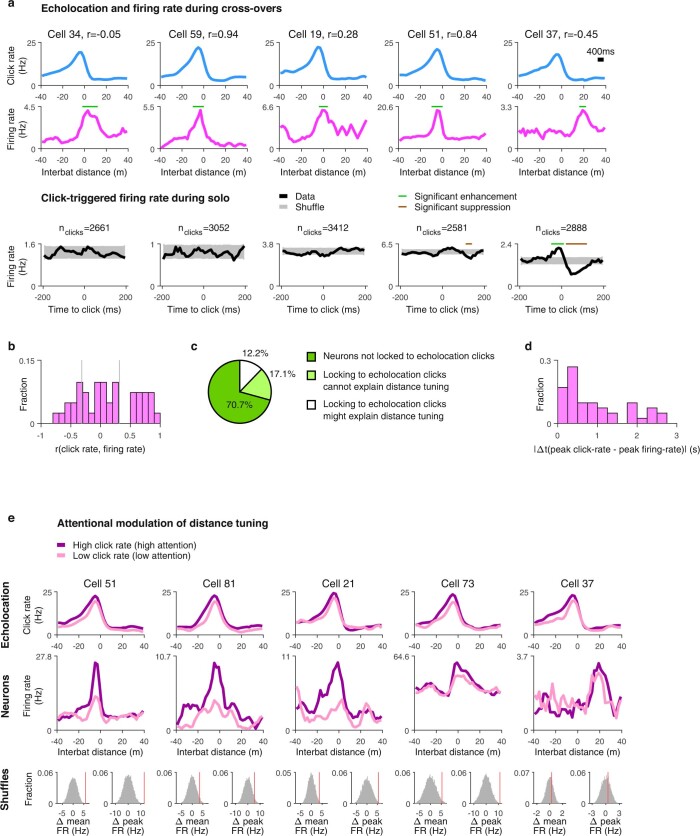
The 1D distance tuning could not be explained by pure sensory responses to echolocation clicks or by motor activity preceding echolocation clicks. All the data included in this figure are taken from bat 2299, in which audio was recorded simultaneously with neurons. **a–d**, Control for sensory or motor responses to individual clicks. To test whether the neuronal modulations found during cross-overs could be explained by sensory or motor responses to clicks, we reasoned as follows: During cross-overs there is a strong behavioural coupling between interbat distance and increase in click-rate (Fig. 1d–f), and thus it is hard to disentangle these variables during cross-overs and reveal the underlying signal driving the neuron. Since during solo the bats also emit echolocation calls (albeit at lower rates compared with cross-overs: Fig. 1e, Extended Data Fig. 2b), we can test the neuronal response to echolocation clicks during solo, where they are not coupled with the cross-over context. If a cell was not modulated by echolocation clicks during solo, it strongly suggests that also during cross-overs this cell was not modulated by pure sensory or motor responses to clicks. **a**, Five examples of 1D distance cells. For each cell: Top, echolocation behaviour: mean click-rate as a function of interbat distance (blue). The scale-bar in the rightmost cell shows the interbat distance that correspond to 400 ms, which is the time-duration of the panels in the third row (see below). Middle, 1D distance tuning curve (during cross-over; pink); horizontal green line denotes significantly enhanced bins. Correlation values above each cell correspond to the Pearson correlation between the click-rate (first row) and the distance tuning curve of the cell (second row). Bottom, click-triggered firing rate during solo (black), and shuffles (grey); n_clicks_ denotes the number of echolocation clicks during solo (which were used to compute the click-triggered firing rate). We focused on a time window of ±200 ms, since this time-scale allows capturing most of the classical motor or sensory responses. Areas with significant enhancement or suppression are marked in the bottom panel by green or brown lines, respectively. Note that cells 34, 59 and 19 showed significant modulation of the 1D distance tuning (middle panels) but showed *no* significant response around the echolocation-clicks during solo (bottom panels). Overall, 70.7% of the cells (*n* = 29) did *not* show any significant responses to echolocation clicks. **b**, Distribution of Pearson correlations between the click-rate and firing-rate tuning curves (correlations between upper panels in **a** and middle panels in **a**; *n* = 41 cells × directions). Since pure sensory and motor responses in bats are known to have short timescales on the order of tens of milliseconds, we expect that a neuron which responds purely to clicks will follow faithfully the click-rate; therefore, its distance tuning will show a strong positive correlation with the click-rate (or negative correlation – depending on the sign of the response). However, the correlations were broadly distributed around zero, with many cells showing low and non-significant correlations (grey lines denote the significance threshold) – suggesting that the distance tuning of many cells cannot be explained by direct sensory or motor responses to clicks. **c**, Percentages of three sub-populations of 1D distance cells recorded in bat 2299: (i) Neurons that were not locked significantly to echolocation clicks during solo (dark green, 70.7% of the cells, *n* = 29 neurons; i.e. no significant responses in **a**-bottom: for example, see the three leftmost neurons in the bottom-most panel in **a**: cells 34, 59, 19). (ii) Neurons that showed significant locking to echolocation clicks during solo (i.e. significant response in the bottom-most panel of **a**), but this locking could *not* explain their distance tuning (light green, 17.1% of the cells, *n* = 7 neurons) – because of two reasons: (1) Their click-rate as a function of interbat distance (panel **a**, top) was *not* significantly correlated with their distance neural tuning (panel **a**, middle; correlations shown in panel **b**); or: (2) The click-triggered locking exhibited an opposite effect from the distance modulation, e.g. cells that had significant *positive* correlation between click-rate and firing rate but had significant *suppression* of firing in their click-triggered response (e.g. cell 51 in panel **a**); or conversely, they had significant *negative* correlation between click-rate and firing rate but *enhancement* of firing in their click-triggered response. (iii) Neurons whose significant locking to echolocation-clicks might potentially explain their distance tuning (white, 12.2% of the cells, *n* = 5 neurons; e.g. cell 37 in panel a [significant negative correlation and negative click-triggered response (suppression)]). Overall, the tuning of 87.8% of the 1D distance cells (sum of the two green areas) could *not* be explained by simple sensory or motor responses to clicks. **d**, Distribution of the absolute time-difference between the peak click-rate (peak of the blue curves in **a**) and the peak firing rate during cross-over (peak of the pink curves in **a**; *n* = 41 cells × directions). Time differences were computed based on tuning-curves that were calculated in time-to-cross-over rather than distance (these two variable are highly correlated, see Extended Data Fig. 4d, e, and thus yield very similar curves). Note that for many cells the peak in firing rate and the peak in click-rate could be more than 0.5 s apart – which is much more than expected from a pure sensory or motor responses in the brain. Further, we note that pure sensory or motor responses also could not explain the complex 2D characteristics of the distance by position tuning shown in Figs. 2 and 4. The rationale for this is as follows: Since the increase in click-rate during cross-overs is very robust and occurs at all positions in the tunnel (Fig. 1f, Extended Data Fig. 2d), then we expect that if a cell purely responds to clicks, we should see similar firing-rate modulation (as a function of distance) at *all* positions. However, we observed many neurons with distance modulations that were restricted to specific positions in the tunnel (Fig. 3a, d, f), as well as non-separable representation of position by distance (shown in Fig. 5a–g) – which rules out this possibility. **e**, Distance modulation of firing rate by attention. Shown are five examples of 1D distance cells, plotted separately for high click-rate flights (purple) versus low click-rate flights (pink). Four leftmost cells are the same neurons as in main Fig. 2e, and are significantly modulated by attention. Rightmost cell (cell 37) is one of the five neurons that were found to be potentially explained by sensory or motor response to clicks (according to the controls in panels **a**–**d**) – yet, it was not significantly modulated here by attention. For each cell: Top, echolocation behaviour: mean click-rate as a function of interbat distance, for high click-rate flights (purple) and low click-rate flights (pink) (see Methods for flight bisection into these two groups of flights). Middle, neuronal responses: distance tuning-curves for high click-rate flights and low click-rate flights. Bottom left: Δ mean firing rate (high–low) for the actual data (red line) and for 10,000 random permutations of the flights (grey histogram); firing rates (FR) were computed in a ±10-m window (see Methods). Bottom right: Δ peak firing rate (high–low) for the actual data (red line) and for random permutations (grey histogram). Note that although the differences in click-rate between high- and low-attention flights were relatively small (top row), the differences in firing rate were prominent in some neurons (middle row), and were highly significant (bottom row; *P* < 0.05 in all tests for the three leftmost cells) – and were also significant across the population (Fig. 2f, g). Finally, we note that the fact that the distance tuning could not be explained by simple responses to clicks (see panels **a**–**d**), suggests that the modulation that we found by high/low click-rate (Fig. 2e–g and panel **e** here) reflects modulation by attention or other high cognitive variable.

**Extended Data Fig. 8:**
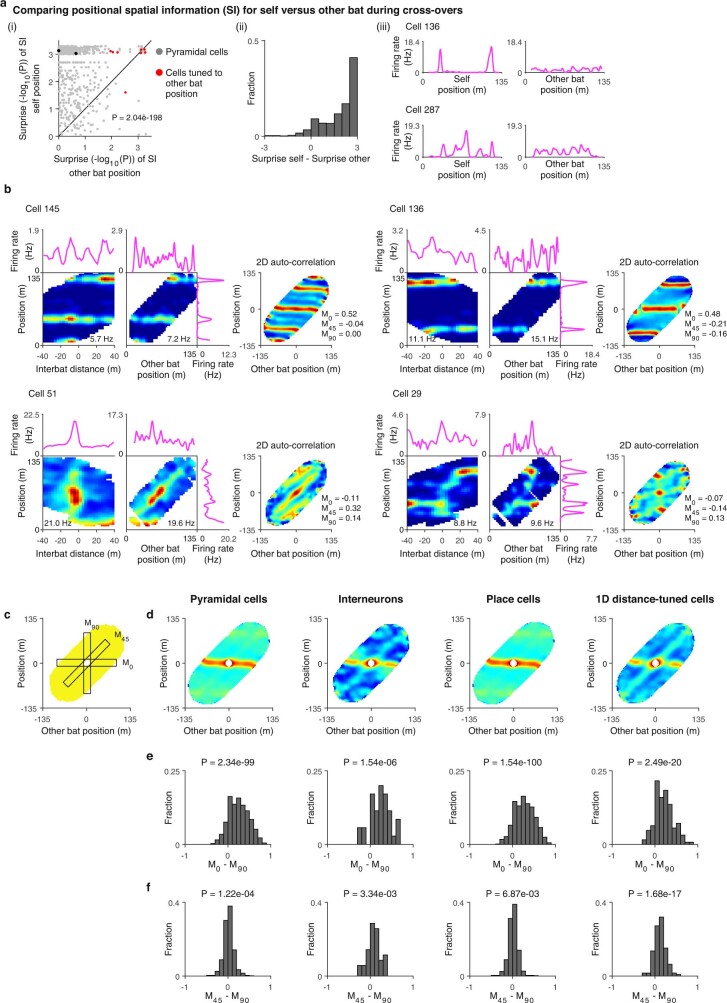
During cross-overs, the position of the other bat (i) was not represented by CA1 neurons, and (ii) could not explain the distance tuning. **a**, No tuning to the position of the other bat. (i) Scatter plot of surprise values (–log_10_(P-value)) for the spatial information (SI) of the tuning-curves during cross-over flights for the self-position (*y*-axis; see the left tuning curves in a(iii)) versus position of the other bat (*x*-axis; see the right tuning curves in a(iii); these two examples are marked on the scatter-plot by two black dots). The position tuning for self was much more prominent that the position tuning for the other bat (paired *t*-test: *P* = 2.04×10^–198^, *n* = 660 pyramidal cells × directions [all the valid pyramidal cells that had ≥ 30 spikes during cross-over]). P-values were computed compared to 1,000 shuffles. For display purposes only, the points with the maximum value in the scatter (surprise = 3) were slightly jittered positively in *x* or *y*, respectively. The high surprise values on the *y*-axis correspond to the very prominent self place-tuning in the hippocampus. By contrast, we note that only 1.2% of the cells were significantly modulated and stable to the other bat’s position (*n* = 8 neurons, marked in red; see also Methods) – which is not different than expected by chance given our 99% significance threshold (Binomial test for population-wide significance [with expected *P*_0_ = 0.01]: *P* = 0.34). We note that none of these 8 cells (one of which is cell 29 in panel **b**) showed convincing representation of the position of the other bat during cross-overs. (ii) Distribution of surprise differences: –log_10_(P_self_) – (–log_10_(P_other_)); i.e. distribution of the *y*–*x* differences for each point in a(i) (*n* = 660 pyramidal cells × directions). (iii) Example cells for this analysis: tuning curve to self-position (left) and to the other bat’s position (right) during cross-overs. Surprise values in panels a(i) and a(ii) were computed based on these two types of position tuning curves. Note the clear tuning to self-position (place-tuning) and the lack of such tuning to the other bat’s position in these two cells. **b–f**, To further explore the possibility that during cross-overs, CA1 neurons represent the position of the other bat, we replotted the data as follows. We constructed a 2D firing-rate map as a function of the self-position on the *y*-axis versus the position of the other bat on the *x*-axis (central maps for each cell in panel **b**, using the same procedure as we did for the 2D distance by position maps). On such 2D maps, a pure place cell would show horizontal stripes with high firing at the positions of its place fields (stripes at angle 0°); a cell that purely represents the other bat’s position would show vertical stripes – at the positions where the cell is responding to the other bat (stripes at 90°); while a 1D distance cell that responds at a specific *distance* from the other bat would show diagonal stripes (at 45°) because the interbat distance is by definition the difference between the *y* and the *x* axes in this panel. Since all three predictions yield stripes in these 2D firing rate maps, we computed the autocorrelations of these maps (2D shifted Pearson correlations map^70,71^; see panel **b**: “2D auto-correlation”) – which are known to emphasize stripes. We then computed for each cell the mean correlation value in the relevant three bands – horizontal: 0°, vertical: 90° and diagonal: 45°, within the 2D autocorrelation map. Each band had a width of 7 bins. Since the diagonal of the 2D autocorrelation map is longer than its horizontal or vertical dimensions, we cropped the diagonal-band from both ends, such that its length was equal to the mean length of the vertical and horizontal bands (see panel **c**, cartoon). For all bands we excluded the central circle in the 2D autocorrelation map (radius of 3.5 bins) – to avoid the dominant central peak in the autocorrelation. A place cell would have high average correlation in the *horizontal* band at 0° (denoted M_0_); a cell representing the other bat’s position would have high average correlation in the *vertical* band at 90° (M_90_); and a 1D distance cell would have a high average correlation in the *diagonal* band at 45° (M_45_). **b**, Four example cells (for each cell the data are shown only during cross-over flights): Left, the standard position-by-distance 2D firing rate map – which shows the firing rate as a function of position (*y*-axis) and interbat distance (*x*-axis); and plotted also is the distance tuning curve (top). Centre, position-by-position 2D firing rate map – which shows the firing-rate as a function of self-position (*y*-axis) and other bat’s position (*x*-axis); the magenta-coloured tuning curves depict the tuning-curve for the self-position (right) and the tuning-curve for the other bat’s position (top). Both of these firing-rate maps are colour-coded from zero (blue) to maximal firing rate (red; value indicated). Right, 2D autocorrelation map (shifted Pearson correlations) of the position-by-position firing rate map of self-position versus other bat’s position (i.e. autocorrelation of the map in the centre; colour-coded from minimum to maximum = 1). Values of M_0_, M_45_, M_90_ for these four neurons are indicated. As expected, place cells (e.g. cells 145 and 136) showed prominent horizontal stripes in their 2D autocorrelation-maps, resulting in high correlation values in the horizontal band (high M_0_; see panel **c** for cartoon of computation); 1D distance cells (e.g. cell 51) showed high correlation in the diagonal band (high M_45_); and cells representing the other bat’s position (cell 29) showed high correlations in the vertical band (high M_90_). We note that cell 29 had one of the highest values of M_90_ compared to its other 2 values – i.e. it was a potential candidate for a neuron representing the other bat’s position – and yet its 2D map and 2D autocorrelation do not show true vertical stripes. In fact, we did not find a single neuron that was convincingly tuned to the position of the other bat in this experiment (see also population analyses in panels **a**, **d**, **e**, **f**) – but rather the dominant signals were the interbat distance and the self-position. We believe that this is probably because: (i) the distance to the other bat is directly available via the bat’s sonar sensory system^2,39^; (ii) in this collision-avoidance experiment it was more behaviourally-important to represent the distance to the other bat (M_45_), in order to avoid collisions – rather than representing the position of the other bat. **c**, Schematic showing the 3 rectangular bands in which we computed M_0_, M_45_ and M_90_ from the 2D autocorrelations (values of M_0_, M_45_ and M_90_ are indicated for each cell in panel **b**): for each band we computed the mean correlation over all of its bins, while excluding the central circle (white; radius 3.5 bins). **d**, Mean 2D autocorrelation maps, computed only over cells with enough behavioural coverage in the 2D map of self-position versus other bat position (cells for which ≥75% of the full 2D behavioural map was covered): these mean 2D autocorrelations are plotted separately for pyramidal cells (*n* = 577 cells × directions), interneurons (*n* = 35 cells × directions), place cells (*n* = 519 cells × directions), and 1D distance cells (*n* = 125 cells × directions). We excluded from the display the central circle (radius 3.5 bins), which is the same circle that we removed for the calculation of M_0_, M_45_ and M_90_ above; we also excluded here bins which comprised < 10 neurons. **e**, Distribution of differences between M_0_ and M_90_, for the same groups of cells as in panel d. Note that for most cells, M_0_ was higher than M_90_ (*P*-values of *t*-tests are shown) – indicating stronger representation of self-position as compared to the representation of the other bat’s position. **f**, Distribution of differences between M_45_ and M_90_, for the same groups of cells as in **d**. Note that in all groups, M_45_ was higher than M_90_ for most cells (*P*-values of *t*-tests are shown) – indicating stronger representation of interbat distance as compared to the other bat’s position. Specifically, we note that for the group of 1D distance cells (rightmost panel), which are of particular interest, the M_45_ values were significantly higher than M_90_ (*t*-test: *P* = 1.68 × 10^–17^) – suggesting that the 1D distance tuning seen in the data could *not* be explained via a representation of the other bat’s position.

During cross-overs, a subpopulation of hippocampal neurons showed significant modulation of their firing rate at specific interbat distances, exhibiting either enhanced or suppressed firing (Fig. 2a). We started by analysing the one-dimensional (1D) tuning to interbat distance, irrespective of position along the tunnel: 18.0% of the pyramidal neurons and 39.2% of the interneurons were classified as significantly tuned to distance (pyramidal, *n* *=* 125 cells × directions; interneurons, *n* *=* 29 cells × directions; Fig. 2b–d and Extended Data Fig. 4a); we refer to cells showing 1D distance modulation as 1D distance cells. This classification was based on three main criteria: (1) rigid spike shuffling for cross-over flights, which preserves the spiking pattern but dissociates it from behaviour; (2) shuffling compared to firing expected from solo flights to account for the place tuning (Extended Data Fig. 5); and (3) stability of the distance tuning (Extended Data Fig. 4b and Methods). These criteria ensured the detection of significant and stable distance tuning that did not result from the place tuning. The distance modulation was very prominent (Extended Data Fig. 4c; mean *z*-scored peak enhancement compared with the firing expected from solo: *z* = 7.18 (pyramidal cells) and *z* = 7.17 (interneurons)). The distance tuning could not be explained by speed changes during cross-overs (Extended Data Fig. 6), nor by direct responses to individual echolocation clicks (Extended Data Fig. 7a–d). Moreover, the distance tuning did not reflect coding of the absolute position of the other bat but, rather, reflected genuine coding of the interbat distance (Extended Data Fig. 8f (right)). In fact, almost no neurons showed significant tuning to the other bat’s position in this behavioural paradigm (Extended Data Fig. 8a; 1.2% of the cells were significant at a 1% significance level: binomial test, *P* = 0.34). The finding that hippocampal neurons encode distance information during cross-overs rules out hypothesis 1, which suggested that there is only pure position coding in the hippocampus.

Across the population, 1D distance-coding cells showed significant modulation at many interbat distances, with over-representation of cells tuned to short distances (Fig. 2b–d; see Extended Data Fig. 4a for individual animals). The response onset of most neurons occurred early: 70.4% of the pyramidal neurons and 93.1% of the interneurons started responding before the cross-over event (Fig. 2d; sign test for the enhancement response onset compared to zero distance: pyramidal neurons, *P* = 1.27 × 10^−4^, *n* = 109 cells × directions; interneurons, *P* = 3.05 × 10^−5^, *n* = 16 cells × directions). Notably, the neuronal distance signal started at a similar interbat distance as the attention signal, indexed by an increased rate of echolocation clicks (the response onset in Fig. 2d starts at approximately −20 m, similar to the initial increase in click rate: Fig. 1e) but the neuronal responses ended later—many pyramidal cells were distance tuned also after the cross-over event (interbat distance > 0), when the echolocation click rate had returned to the baseline. This might be explained as follows: (1) while most neurons were ‘switched on’ by attention before the cross-over event, these cells continued firing for some time, and then other neurons became active after cross-over due to network reverberations or neuromodulators. (2) Some pyramidal cells with late activity may have been released from inhibition (Fig. 2c; the bottom ~20 pyramidal cells show suppression followed by enhancement), which could stem from interneurons being most strongly active before and around the cross-over event (compare pyramidal cells and interneurons in Fig. 2b–d (top)). (3) Population activity both before and after cross-over could represent neuronal sequences^24–26^, perhaps reflecting memory encoding of the entire cross-over event. (4) Finally, it might be behaviourally relevant to represent the distance from the other bat also after the cross-over event because the other bat could perform a U-turn after the cross-over and fly back towards the recorded bat (indeed, we observed such U-turns occasionally in the experiment). Furthermore, bats can directly sense the distance to the other bat behind their back using echolocation, which spreads also backwards^5,27^; or they can estimate the distance to the other bat after cross-over using path integration by relying on the bats’ fixed speed.

The distance tuning was generally uncorrelated between the two flight directions (Extended Data Fig. 4h–j) and was also uncorrelated between positive and negative distances (Extended Data Fig. 4k). This suggests that the distance-tuned neurons did not encode absolute distance but, rather, encoded distance and direction. Thus, these neurons could be interpreted as vectorial distance cells (other types of vectorial cells in the hippocampal formation were described previously^18,19,21,23^).

To explicitly test whether attention modulates the 1D distance tuning, we performed several analyses. First, we bisected the cross-over flights into those with lower attention versus those with higher attention (lower versus higher click rate), and found that cells with 1D distance tuning exhibited stronger responses during high-attention flights (Fig. 2e–g and Extended Data Fig. 7e; bisection was performed for entire cross-over flights; we included here only cells recorded simultaneously with audio; Methods). As distance tuning could not be explained by direct responses to echolocation clicks (Extended Data Fig. 7a–d), these results suggest that 1D distance neurons are modulated by high-level cognitive variables, such as attention, arousal or an enhanced state of active sensing.

**Extended Data Fig. 9:**
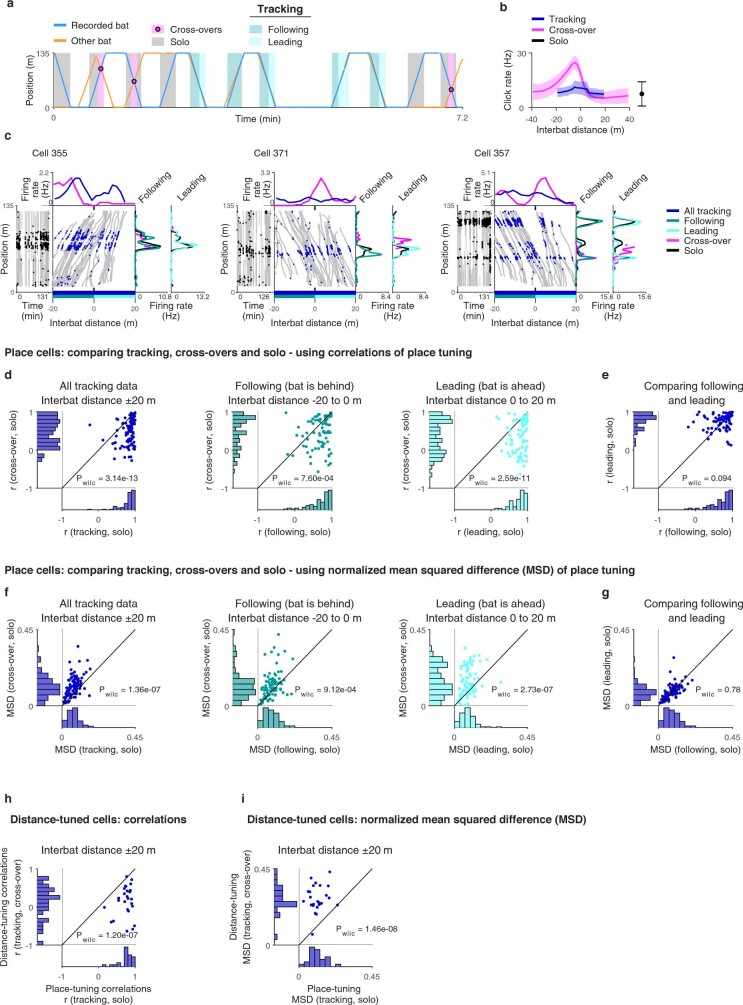
During tracking behaviour CA1 neurons were not modulated by interbat distance and preserved their position tuning. All the data included in this figure are taken from bat 30, which performed also tracking behaviour. **a**, An example of a 7.2-min epoch from one session, which included both solo, cross-over, and tracking behaviours. Plotted are the positions of the two bats – the recorded bat in blue line and the other bat in orange line. Cross-over events are marked with pink circles, and cross-over flights (window of ±40 m of interbat distance around the cross-over event) are marked with pink rectangles. Solo flights are marked with grey rectangles. Tracking behaviours are marked with 2 different aquamarine rectangles: (i) *Following*: when the recorded bat was behind the other bat, with an interbat distance between −20 m to 0 m. (ii) *Leading*: when the recorded bat was ahead of the other bat, with interbat distance between 0 m to 20 m. **b**, Population average of the click-rate during cross-overs (pink), tracking (blue), and solo (black); shaded colours and black error-bar indicate mean ± s.d (*n* = 426 cross-over flights, *n* = 602 tracking flights, and *n* = 64,454 solo time bins). Note that during tracking, the bats did not increase their click-rate as much as during cross-over – suggesting that tracking behaviour is less attentionally-demanding than collision-avoidance behaviour during cross-overs. **c**, Three example neurons. Top, 1D distance tuning-curve during tracking (dark blue) and during cross-over (pink). Bottom left, spike raster during solo flights (black dots), showing position (*y*-axis) versus time (*x*-axis). Central large panel, position of the recorded bat (*y*-axis) and interbat distance (*x*-axis) during tracking (grey) with spikes overlaid (dark blue). Right two panels, position tuning curves (place-tuning) of the cell during solo flights (black), during following (left; aquamarine line) or during leading (right; light aquamarine line), and during cross-over flights (pink; computed over −20 to 0 m or 0 to 20 m, which is the same distance-range as following and leading). Note that in all three examples, the position tuning-curves during tracking (right) were very similar to the position tuning-curves during solo flights; and that the distance tuning-curves during tracking (top: dark blue line) were rather flat for two of these three neurons, and were very different from the distance tuning-curve during cross-overs (top: pink line). **d–g**, Population summaries of place cells that were recorded in bat 30 in the tracking condition (*n* = 91 place cells × directions in total). **d**, In all panels: the *y*-axis is the Pearson correlations between position tuning during cross-overs and during solo flights, and the *x*-axis is the Pearson correlations between position tuning during tracking flights and solo flights. Histograms show marginal distributions. Left – all tracking data (Wilcoxon rank sum test of *y* versus *x* for the dots, *P* = 3.14 × 10^–13^). Middle – Following (Wilcoxon rank sum test, *P* = 7.60 × 10^−4^). Right – Leading (Wilcoxon rank sum test, *P* = 2.59 × 10^–11^). In all panels, the position-tuning correlations between tracking and solo were high, and significantly higher than the position-tuning correlations between cross-over and solo: This suggests that the solo position-tuning of place cells was *not* strongly altered during tracking behaviours, and thus the position tuning remained essentially the same during tracking and solo. **e**, Comparing following and leading. Shown is the Pearson correlation between position tuning during leading and during solo flights (*y*-axis) versus the Pearson correlation between following and solo flights (*x*-axis). Panel plotted as in **d**. Note the high correlations between both following and solo, and leading and solo: these correlations were not significantly different from each other, i.e. between the following and leading conditions (Wilcoxon rank sum test, *P* = 0.094). **f**, Similar to **d**, but with normalized mean squared difference (MSD) instead of the Pearson correlation. MSD was defined as: ${\rm{M}}{\rm{S}}{\rm{D}}=\langle {({f}_{1}-{f}_{2})}^{2}\rangle /(max({f}_{1},{f}_{2})-min({f}_{1},{f}_{2}))$, where *f*_1_ and *f*_2_ are the two position tuning-curves; the numerator thus denotes the mean of the sum of squared differences between the position tuning-curves (*n* = 91 place cells × directions). Left – all tracking data (Wilcoxon rank sum test, *P* = 1.36 × 10^−7^). Middle – following (Wilcoxon rank sum test, *P* = 9.12 × 10^−4^). Right – leading (Wilcoxon rank sum test, *P* = 2.73 × 10^−7^). In all panels, the position-tuning MSD between tracking and solo was significantly lower than the position-tuning MSD between cross-over and solo: as in panel **d**, this suggests that the position tuning remained essentially the same during tracking and solo. **g**, MSD between position tuning during following and solo flights (*x*-axis) and between leading and solo flights (*y*-axis). Panels plotted as in **f**. Note that MSD values between solo and following and between solo and leading are both low – indicating similar tuning – and are not significantly different between following and leading (Wilcoxon rank sum test, *P* = 0.78). **h–i**, Population summaries of significant 1D distance cells (defined by significant modulation during cross-overs) recorded in bat 30 in the tracking condition (*n* = 27 cells × directions). **h**, Pearson correlations between the *position* tuning-curve during tracking and during solo flights (*x*-axis) versus the Pearson correlations between *interbat distance* tuning-curves during tracking and during cross-over flights (*y*-axis) (Wilcoxon rank sum test, *P* = 1.20×10^−7^). This scatter-plot suggests that cells which are distance-tuned during cross-over flights do *not* preserve their distance tuning during tracking (note the large spread of correlations along the *y*-axis) – while they do preserve their solo position tuning during tracking (note the high correlations in the *x*-axis). **i**, Normalized MSD (see panel **f** for details), computed between the *position* tuning-curves during tracking and during solo flights (*x*-axis) versus the normalized MSD computed between the *interbat distance* tuning-curves during tracking and during cross-overs flights (*y*-axis) (Wilcoxon rank sum test, *P* = 1.46 × 10^−8^). As in panel **h**, this scatter-plot suggests that during tracking, 1D distance cells do *not* maintain their distance tuning as in cross-overs – but *do* preserve their position tuning during tracking as in solo flights. Moreover, when analysing the distance tuning during tracking in the same way as for the cross-over data, we found a low percentage of cells that had significant distance tuning during tracking: Only 5.0% of the pyramidal neurons (*n* = 5 cells × directions) and 16.1% of the interneurons (*n* = 5 cells × directions) were significantly modulated by distance during tracking.

Second, to experimentally test the effect of attention, we trained one pair of bats to perform an additional behaviour—tracking—in which the two bats flew in the same direction at a short interbat distance (Extended Data Fig. 9a (cyan and turquoise rectangles) and Methods). We reasoned that, as the relative speed during tracking was near-zero (in contrast to cross-over, for which it was around 14 m s^−1^), tracking requires lower attention than cross-over. Indeed, bats echolocated at much lower rates during tracking (Extended Data Fig. 9b), suggesting that tracking requires less attention. During tracking flights, CA1 neurons preserved their place tuning, but did not preserve their distance tuning; in fact, distance tuning was largely absent during tracking (Extended Data Fig. 9). This suggests that only when attention is strongly required—during cross-overs, but not during tracking—CA1 neurons encode the interbat distance.

**Extended Data Fig. 10:**
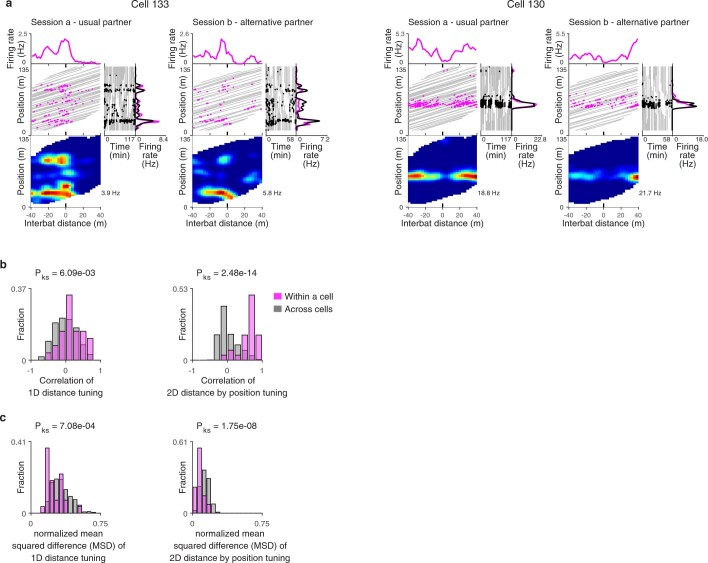
Distance representation was largely invariant to the identity of the other bat. **a**, Two example cells recorded in the switching-partner sessions (Methods). For each cell: shown are session *a* with the usual partner (left) and session *b* with an alternative partner (right); the recorded bat was trained with both bats before the recordings. Both sessions were recorded in the same day, with a break between them for rest. Spike sorting was done across both sessions together, and cells were verified to be stable throughout both sessions. Data plotted as in main Fig. 2a. These two cells exhibited rather similar 2D distance-by-position tunings in both sessions – suggesting that the distance coding is invariant to the identity of the other bat. **b–c**, Comparing session *a* and session *b* (for all 4 panels we show all the *n* = 27 cells × directions which were significant 1D or 2D distance cells). **b**, Left, correlations of the 1D distance tuning-curves between session *a* and session *b*, computed within-cells (pink) and across different cells (shuffles, grey) (Kolmogorov-Smirnov test of pink versus grey: *P* = 6.09 × 10^−3^, Wilcoxon rank sum test: *P* = 3.02 × 10^−3^). Right, correlations of the 2D distance by position firing-maps between session *a* and session *b* (Kolmogorov-Smirnov test: *P* = 2.48 × 10^–14^, Wilcoxon rank sum test: *P* = 1.45 × 10^–14^). **c**, Similar to panel **b**, but with normalized mean squared difference (MSD) instead of the Pearson correlation. MSD was defined as: ${\rm{M}}{\rm{S}}{\rm{D}}=\langle {({f}_{1}-{f}_{2})}^{2}\rangle /(max({f}_{1},{f}_{2})-min({f}_{1},{f}_{2}))$, where *f*_1_ and *f*_2_ are the two tuning-curves. Left, MSD of the 1D distance tuning-curves between session *a* and session *b* (Kolmogorov-Smirnov test of pink versus grey: *P* = 7.08 × 10^−^^4^, Wilcoxon rank sum test: *P* = 2.99 × 10^−5^). Right, MSD of the 2D distance by position firing-maps between session *a* and session *b* (Kolmogorov-Smirnov test: *P* = 1.75 × 10^−8^, Wilcoxon rank sum test: *P* = 3.15 × 10^–9^). The histograms in panels **b**–**c** show that the correlations between sessions *a* and *b* were higher than chance (shuffle) and the MSD values were lower than chance (shuffle). Taken together, these results indicate that the distance code was largely invariant to the other bat’s identity, suggesting that the distance code might be related to collision-avoidance rather than to a social representation – likely because when flying at high speed, the bats care mostly about collision-avoidance, and less about the identity of the other bat they are avoiding collision with. Future experiments could potentially use drones – flying inanimate objects – to further test whether these neurons carry a social signal.

Third and finally, in another pair of bats, after the end of the regular behavioural session, we conducted a second session in which the recorded bat flew with an alternative partner bat. We reasoned that, during collision avoidance (cross-over), it is important to attend to the other bat and represent its distance irrespective of the other bat’s identity. Indeed, distance tuning was largely preserved between the two sessions with different partners (Extended Data Fig. 10). This suggests that the observed distance tuning (Fig. 2) is probably not social but, rather, is related to collision avoidance.

## Conjunctive 2D coding of distance by position

**Fig. 3:**
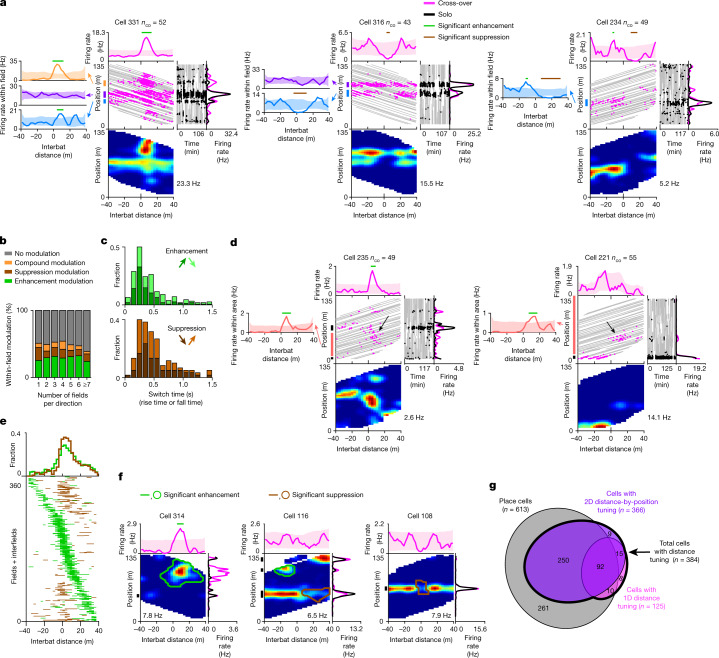
Conjunctive representation of interbat distance by position. **a**, Examples of three neurons. For each cell, the middle and right plots are as described in Fig. 2a, and the left plot shows the 1D distance tuning curves within different place fields (colours correspond to different place fields, marked by vertical coloured lines on the left of the centre plot). Place fields here and elsewhere were defined on the basis of solo data. Shading shows cross-over shuffles; horizontal green and brown lines show bins with significant enhancement and suppression, respectively. **b**, The percentages of different types of distance modulation within place fields for neurons with different numbers of place fields. Compound modulation indicates tuning with both significant enhancement and suppression (for example, cell 234 (blue) in **a**). **c**, The distribution of neuronal switch times of the distance tuning. Top, the rise time (dark green) and fall time (light green; stacked) for place fields with significantly enhanced distance tuning curves. *n* = 143. Bottom, the fall time (dark brown) and rise time (light brown) for place fields with significantly suppressed distance tuning curves. *n* = 62. Only a subset of the fields was valid for analysis here (Methods). **d**, Examples of two neurons with significant enhancement outside their place fields (within an interfield), plotted as described in **a**. The vertical lines on the left of the centre panel mark place fields (black) and interfields (peach). The black arrows indicate spikes contributing to distance tuning within the interfield; note that there were barely any spikes in the same position during solo flights (see the solo raster on the right). **e**, Population summary: distance bins that were significantly enhanced (green) or suppressed (brown) within place fields (*n* = 301 fields) and interfields (*n* = 59) sorted by distance-tuning peak. **f**, 2D distance by position tuning: patch analysis. Examples of cells with significant 2D patches are plotted as described in Fig. 2a without the raw data panels. The outlines show significant 2D patches (enhancement (green); suppression (brown)). The vertical black lines show place fields. **g**, Summary of different functional classes of pyramidal cells (numbers denote cells × directions). Place cells are shown in grey. The thick black curve encompasses the total number of distance-modulated cells (*n* = 384, 55.4% of all pyramidal cells). Source data

**Extended Data Fig. 11:**
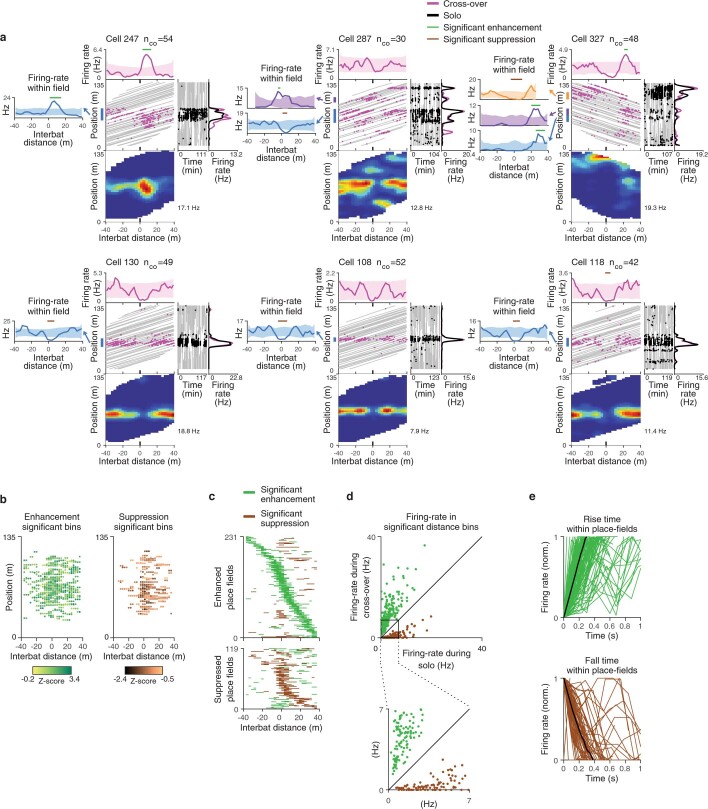
Tuning to interbat distance within place fields. **a**, Additional examples of 6 place cells with significant distance modulation within place fields. Plotted as in Fig. 3a. Note that cell 287 has 2 fields: one field exhibits enhancement during cross-over while the other exhibits suppression – therefore, the two fields cancel each other in the overall 1D distance tuning of this cell, resulting in non-significant distance tuning (pink top tuning-curve). This example emphasizes the need to compute distance tuning-curves within place fields, as well as to perform 2D analysis on the entire 2D map. **b**, Population summary: Position of the place fields (mean position of the place field edges; *y*-axis) versus the interbat distance of significantly distance-modulated bins (*x*-axis); plotted are the significant enhancement bins (left) and significant suppression bins (right); colour of dots depicts the *z*-score of the distance tuning curve. **c**, Distance bins, as in Fig. 3e, plotted here only for significant place-fields (without the interfields that were included in Fig. 3e). Top: significantly enhanced fields, sorted by the peak-distance of the distance tuning-curve. Bottom: significantly suppressed fields, sorted by the trough-distance of the distance tuning-curve. Place fields with compound modulation (exhibiting both enhancement and suppression) appear in both the top panel and bottom panel. **d**, Scatter-plot of the mean firing rate within the significant distance bins during cross-overs (*y*-axis) versus the mean firing rate estimated from the solo-projected on cross-over shuffle for the same distance bins during solo (*x*-axis). By definition, enhancement bins (green) should be above the diagonal identity line (black) and suppression bins (brown) should be below the identity line – however, we note that the dots here were *far* away from the identity line, reflecting an average 5-fold increase of firing rate for enhancement responses, and 10-fold decrease for suppression responses (the ratio between the firing rates was 5.26 ± 9.71 for enhancement bins (mean ± s.d.; $Firing\;rate\;during\;cross\;over/Firing\;rate\;during\;solo$) and 9.76 ± 7.82 for suppression bins (mean ± s.d; $Firing\;rate\;during\;solo/Firing\;rate\;during\;cross\;over$). Inset, zoom-in on 0 to 7 Hz on both axes: Note that even at low firing rates the differences in firing rates between solo and cross-over were highly prominent, i.e., the dots were very far from the diagonal identity line. **e**, Tuning curves of switch times within place-fields (normalized min-to-max; top, rise-time for enhancement tuning, *n* = 143 place fields; bottom, fall-time for suppression tuning, *n* = 62 place fields). The *x*-axis shows the time from crossing 50% of the shuffles. Black curve, median response across all place-fields. Note that most of the tuning curves reached their maximum (or minimum) response within ~300 ms, and some tuning-curves exhibited a rise-time as fast as 100 ms, or even faster.

Next, we considered the 2D tuning of neurons to distance by position. As the vast majority of CA1 pyramidal neurons in the long tunnel were place cells^14^ (Extended Data Table 1), we started by analysing the distance tuning curve separately within each place field. Half of the place fields (49.3%, 301 out of 611) were significantly modulated by the interbat distance, showing enhancement, suppression or both (see Fig. 3a and Extended Data Fig. 11a for examples and Fig. 3b for the population). Interestingly, different place fields of the same neuron could exhibit different distance tuning (Fig. 3a (cell 331) and Extended Data Fig. 11a (cell 287)); we return to this issue below. Overall, place cells encoded the position of the bat when flying alone but, during cross-overs, they conjunctively encoded distance by position, and then switched back to their position coding after the two bats passed each other (Fig. 3a). As the bats flew very fast, these switches between different representations were extremely fast—as fast as 100–200 ms (Fig. 3c and Extended Data Fig. 11e).

**Extended Data Fig. 12:**
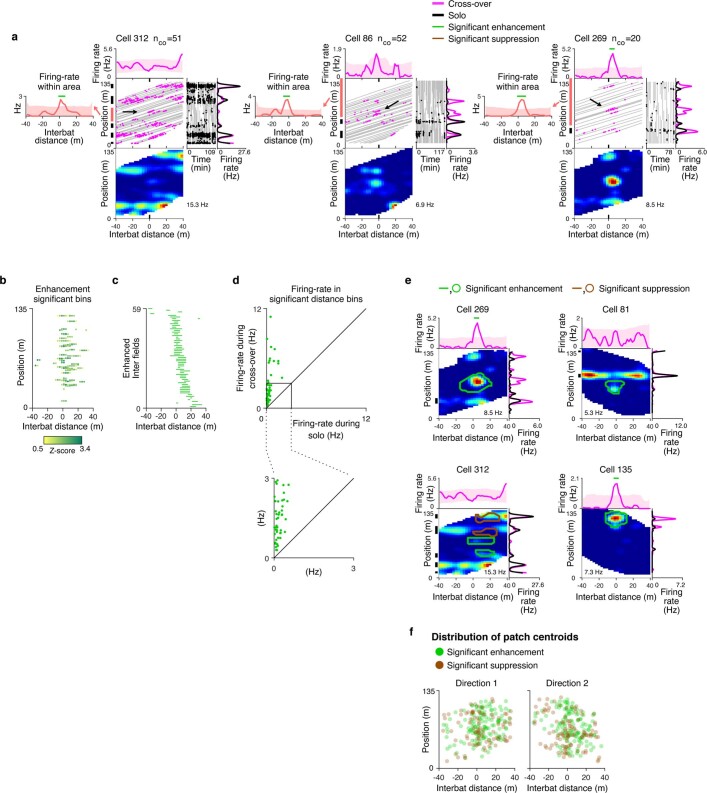
Tuning to interbat distance outside of place fields. **a**, Additional examples of three neurons with significant enhancement within ‘interfields’; plotted as in Fig. 3d. Vertical lines to the left of the central panel mark the place fields (black) and the interfield area that we analysed here (peach-coloured). Black arrows inside the central raw data panels indicate spikes contributing to the distance tuning within the interfields. Some of these interfield regions were areas in the tunnel where during solo-flights the neuron showed almost no activity (see cell 269 here, and Fig. 3d cell 235: note that in the solo-raster [right, black dots] there are almost no spikes within the interfield area). In other neurons, these inter-field regions showed some low firing rate during solo, albeit too low to be detected as a place field (cells 312 and 86 here, and Fig. 3d cell 221). This suggests that the sub-threshold position-inputs that underlie these sub-threshold place fields^28^, might be enhanced by incoming distance inputs and thus rendered supra-threshold – resulting in distance by position increase in firing rate (see also Extended Data Fig. 16). **b**, Population summary: Position of the interfields (mean position of the interfield edges; *y*-axis) versus the interbat distance of significantly distance-modulated bins (*x*-axis); colour of dots depicts the *z*-score of the distance tuning curve. **c**, Distance bins, as in main Fig. 3e, plotted here only for interfields; sorted by the peak-distance of the distance tuning curves. Note that the significantly tuned interfields show only enhancement; we could not detect suppression because, by definition, interfields have very low firing rate to begin with. **d**, Scatter-plot of the firing rate within the significant distance bins during cross-overs (*y*-axis) versus the firing rate estimated from the solo-projected on cross-over shuffle for the same distance bins during solo (*x*-axis). By definition, enhancement bins (green) should be above the diagonal identity line (black) – however, we note that the dots here were *far* from the identity line, and the ratio between the firing rates was 18.50 ± 31.79 (mean ± s.d.; $Firing\;rate\;during\;cross\;over/Firing\;rate\;during\;solo$). Inset, zoom-in on 0 to 3 Hz on both axes: Note that even at very low firing rates, the differences in firing rates between solo and cross-over were highly prominent, i.e., the dots were very far from the diagonal identity line. **e**, Additional examples of four neurons with 2D patches showing significant enhancement or suppression within ‘interfield’ areas (Methods); plotted as in Fig. 3f. Cells 312 and 269 are the same as in panel **a**; note that the 2D patch analysis captures well the extra firing within the cells’ interfields (compare to the raw data in **a**). Cell 135 is an example of a neuron *without* place tuning (it did not pass the criterion for significant place cells during solo) – and yet it showed localized distance-by-position modulation, which was detected by the 2D patch analysis. Finally, we note that cell 81 shows a significant-enhancement 2D patch that occurred outside of place fields (see the green outline) – and there was also a slight reduction in firing rate within the main place field, which was too mild to be detected as significant. **f**, Position and interbat distance of the centroids (centre-of-mass) of all the significant 2D patches (direction1: 134 enhancement patches, 88 suppression patches; direction2: 131 enhancement patches, 103 suppression patches).

Interestingly, significant distance tuning was also seen outside of place fields, in areas defined as interfields (*n* = 59 interfields showed significant responses, out of 87 interfields valid for analysis; Fig. 3d and Extended Data Fig. 12a (black arrows) and Methods). These distance-tuned responses in interfield areas were very strong compared with the low firing rate during solo flights (Extended Data Fig. 12d). This type of distance response might be explained by the presence of subthreshold place fields^28^, which are enhanced by incoming distance inputs and rendered suprathreshold, therefore creating a distance-by-position response. Overall, the distance-tuned place fields and interfields spanned a wide range of distances, with an over-representation of short interbat distances (Fig. 3e and Extended Data Figs. 11c and 12c).

We further examined the existence of significant 2D distance-by-position modulation of firing rate irrespective of place field definitions. We used cluster analysis (Methods) and identified ‘2D patches’ in the 2D distance-by-position firing-rate maps, which were significantly enhanced or suppressed relative to solo (see Fig. 3f for examples and Extended Data Fig. 12f for the population; 9.7% of the significant patches occurred outside of place fields; Fig. 3f (cell 314) and Extended Data Fig. 12e). When considering collectively cells with 2D distance modulation (neurons with significant patches, or significant tuning within-field or interfield; Fig. 3g (purple ellipse)) and cells with 1D distance modulation (Fig. 3g (pink ellipse)), the majority of pyramidal cells in CA1 were significantly modulated by the interbat distance during cross-overs (55.4%, *n* = 384/693 cells × directions; Fig. 3g (thick black line)). The existence of 2D distance-by-position patches rules out hypothesis 2—which suggested a switch between pure position coding and pure distance coding—because both signals were conjunctively encoded during cross-overs. In fact, there are two versions of hypothesis 2—(1) separate populations of neurons encode position and distance, or (2) single neurons switch between purely representing position and purely representing distance—and our results ruled out both options.

**Fig. 4:**
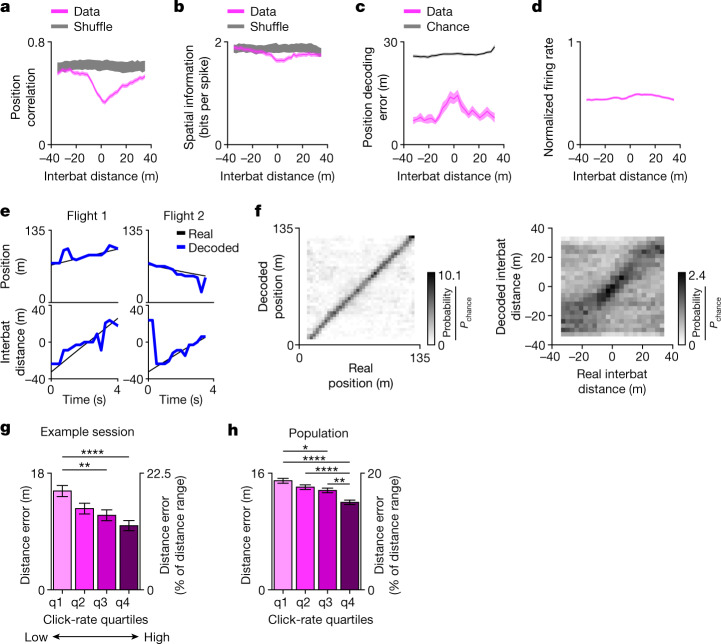
Simultaneous decoding of interbat distance and position. **a**–**d**, Change of position tuning during cross-overs. **a**, The average Pearson correlation between position tuning during solo and during cross-over flights, computed in 10-m distance bins (Methods). Data are mean ± s.e.m. (pink). *n* = 613 place cells × directions. Note the correlations decreased when bats approached each other, indicating a change in position tuning. The grey curve was computed as described for the pink curve, using solo projected on cross-over shuffles. **b**, Spatial information (mean ± s.e.m.; *n* = 613 place cells × directions) for the position-tuning curves computed during cross-over flights (pink), and for solo projected on cross-over shuffles (grey). **c**, Position decoding error as a function of distance during cross-over (pink; mean ± s.e.m.; *n* = 16 sessions) and chance level (grey). Note the increased decoding error at short distances. **d**, Firing rate (normalized to the peak of each cell) as a function of distance during cross-over for all place cells. Data are mean ± s.e.m. *n* = 613 place cells × directions. **e**,**f**, Simultaneous decoding of the interbat distance and position during cross-over flights. **e**, Examples of two flights (columns). Top, real position (black) and decoded position (blue) versus time. Bottom, real and decoded interbat distance. **f**, Confusion matrices (pooled over all sessions with ≥10 cells recorded simultaneously; *n* = 16 sessions). Left, the probability of decoded positions (*y* axis) for each real position (*x* axis), normalized to the uniform chance probability *P*_chance_ = 1/*n*_bins_ (colour bar). Right, the same for the interbat distance. The diagonal structure in these matrices indicates accurate and unbiased decoding. **g**,**h**, The decoding error of interbat distance for different click-rate quartiles (q1–q4, quartiles of low click rate to high click rate) for one example session (**g**) (*n* = 146, *n* = 134, *n* = 146 and *n* = 151 decoding time windows for q1–q4) or pooled over all nine sessions that had audio recordings and ≥10 cells (**h**) (*n* = 998, *n* = 1,051, *n* = 1,074 and *n* = 1,063 decoding time windows for q1–q4). Note that the decoding error decreased as the click rate increased (q4, maximal click rate, that is, maximal attention; click rate is a proxy of attention). Data are mean ± s.e.m. Statistical analysis was performed using analysis of variance with post hoc correction for multiple comparisons: *P < 0.05, **P < 0.01, ***P < 0.001, ****P < 0.0001; no asterisks, not significant. See Extended Data Fig. 13c for the exact *P* values, violin plots and Kruskal–Wallis tests. Source data

Two remaining hypotheses may underlie the results presented so far: hypotheses 3 and 4. Hypothesis 3 suggests multiplexing of position and distance information by the neurons—that is, the neurons exhibit 2D distance-by-position tuning, and are continuously ready to process both of these incoming information streams. Hypothesis 4 suggests that individual hippocampal neurons exhibit switching from position coding during navigation to conjunctive coding of distance by position during collision-avoidance behaviour. We believe that hypothesis 4, that is, switching, is more probable because we found that (1) during cross-overs, the position tuning changed significantly compared with solo flight (Fig. 4a), accompanied by a small but significant decrease in spatial information (Fig. 4b); furthermore, there was a substantial increase in the position decoding error at short interbat distances, when we used the solo-based tuning for decoding (Fig. 4c). Importantly, the changes in the position tuning (Fig. 4a–c) could not be explained by changes in the firing rate because there was no prominent change in the average firing rate during cross-overs (Fig. 4d). This suggests that the distance information comes partially at the expense of the position tuning, as expected from a switch. (2) The rise time of neuronal responses during cross-over was independent of the flight speed (Extended Data Fig. 6k). This is consistent with a neural switch rather than multiplexing, because flight through a static multiplexed tuning curve of distance by position should have yielded a faster rise time at a higher flight speed, which we did not observe (Extended Data Fig. 6k; *t*-test: *t* = 0.84, *P* = 0.41; *n* = 120 neurons). By contrast, for a neuronal switch, we expect that the switch time will have a fixed duration irrespective of velocity, consistent with our results. (3) The neuronal modulation was extremely rapid (Fig. 3c and Extended Data Fig. 11e), consistent with a neuronal switch. (4) Most neurons responded in sync at similar distances (Fig. 2b–d), and this was particularly prominent in each animal separately (Extended Data Fig. 4a). (5) These highly visual bats probably see each other from much greater distances than 20 m (refs. ^29,30^), and yet they exhibited a substantial behavioural and attentional switch at a distance of −20 m (Fig. 1d–f), and the neurons mostly started responding at −20 m (Fig. 2d), which seems to be more consistent with a switch, or gating of neuronal coding. (6) Hippocampal neurons did not always encode distance-by-position information; during tracking, the distance coding was almost absent (Extended Data Fig. 9). This is inconsistent with multiplexing of information, and more consistent with neuronal switching that is based on behavioural and attentional demands. However, in contrast to the switching notion, neurons also exhibited a diversity of preferred distances, consistent with multiplexing of information rather than with a network-level switch. Thus, the data are partially consistent with both hypothesis 3 (multiplexing) and hypothesis 4 (neural switching).

**Extended Data Fig. 13:**
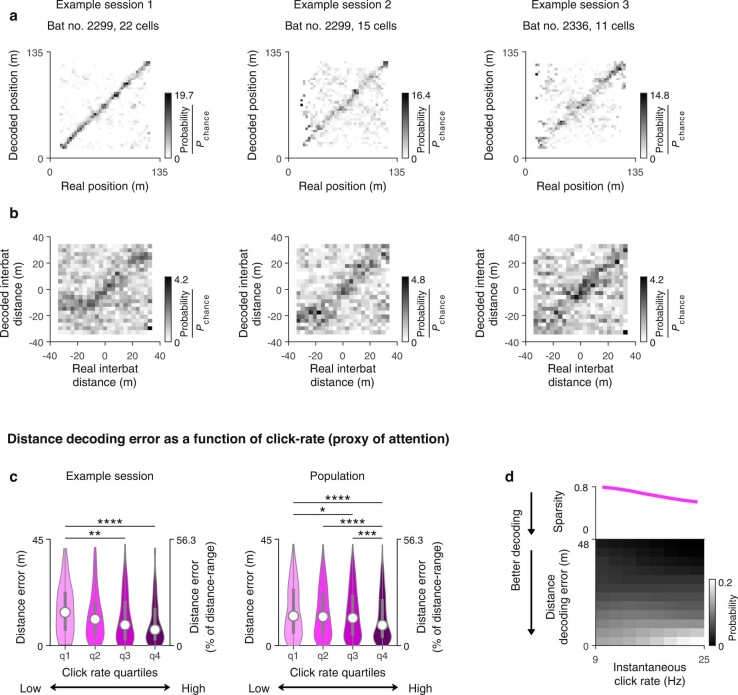
Decoding analysis. **a, b**, Confusion matrices for three example sessions (from two different bats), showing decoding of position (**a**) and decoding of distance (**b**). Plotted as in Fig. 4f. Bin size, 3×3 metres. The number of simultaneously-recorded cells is shown. A diagonal structure in these matrixes indicates good decoding. **c–d**, Higher echolocation click-rate (heightened attention) improves the distance decoding-error. **c**, Decoding error as a function of attention (same data as in Fig. 4g, h, but here plotted as violin-plots), for one example session (left; *n* = 146, 134, 146, 151 decoding time-windows for q1-q4, respectively), and for all the 9 sessions in which we had audio recordings and ≥10 cells (right; *n* = 998, 1051, 1074, 1063 decoding time-windows for q1-q4, respectively). White circle, median; thick grey line, 25–75 percentiles. Note the decoding error decreased as the click-rate increased (q4: maximal click-rate, i.e. maximal attention). Kruskal-Wallis test:* *P*  < 0.05, ** *P* < 0.01, *** *P* < 0.001, **** *P* < 0.0001; no stars means non-significant test. Exact *P*-values for left panel: q1 and q2, *P* = 0.14; q1 and q3, *P* = 3.71 × 10^−3^; q1 and q4, *P* = 1.23 × 10^−5^; q2 and q3, *P* = 0.64; q2 and q4, *P* = 0.07; q3 and q4, *P* = 0.55. Exact *P*-values for right panel: q1 and q2, *P* = 0.44; q1 and q3, *P* = 0.04; q1 and q4, *P* = 4.51 × 10^–9^; q2 and q3, *P* = 0.62; q2 and q4, *P* = 3.36 × 10^−6^; q3 and q4, *P*=7.69×10^−4^. Exact P values for ANOVA in main Fig. 4g: q1 and q2, *P*=0.11; q1 and q3, *P* = 8.80 × 10^−3^; q1 and q4, *P* = 3.38 × 10^−5^; q2 and q3, *P* = 0.83; q2 and q4, *P* = 0.13; q3 and q4, *P*=0.53. Exact P values for ANOVA in main Fig. 4h: q1 and q2, *P* = 0.23; q1 and q3, *P* = 0.02; q1 and q4, *P* = 4.32 × 10^–9^; q2 and q3, *P* = 0.75; q2 and q4, *P* = 2.20 × 10^−5^; q3 and q4, *P* = 1.52 × 10^−3^. **d**, Instantaneous click-rate analysis: the instantaneous click-rate was calculated for each of the decoding time bins (1 s), at interbat distances of –15 m to 0 m (where the increases in click-rate are most prominent). Bottom: probability matrix for distance decoding error (*y*-axis) across different instantaneous click-rates (*x*-axis); each column of the matrix is a probability distribution, i.e. each column sums to 1. The matrix was smoothed using a 2D Gaussian with σ = 1.5 bins. Top: sparsity for the different click-rate columns of the probability matrix (${\rm{sparsity}}={\left\langle {r}_{i}\right\rangle }^{2}/\left\langle {r}_{i}^{2}\right\rangle$, where *r*_i_ are the distance decoding-error values in each bin of each column; higher sparsity denotes a more uniform distribution of decoding-errors within the column). Note that as attention increased (higher click-rate), both the probability of decoding-errors became less uniformly distributed (sparsity decreased, see magenta curve) – and also the prevalence of small errors became much higher (note the white colour at the bottom-right corner of the matrix). In other words, the distance coding became better for high click-rate (high attention). This analysis is complementary to Fig. 4g, h and to panel **c** in the current figure, where click-rate was calculated per-flight – here, by contrast, we computed the instantaneous click-rate, using finer bins of click-rate. Finally, we note that our simultaneous-decoding analysis of distance and position worked surprisingly well, but not as well as reported in the rodent literature. This difference might stem from the following: (i) We conducted simultaneous decoding of two variables, while most studies decode only one variable, namely position. (ii) We recorded in freely flying bats, therefore we were limited by the number of simultaneously-recorded cells per day (13.19 ± 3.08 neurons per day, mean ± s.d.). (iii) We used here a relatively long integration time window of 1 s (Methods), both to account for the low number of simultaneously-recorded cells, and to allow accumulation of enough spikes. However, this long integration time came with a cost: during this 1-second window the bat progressed 7 m in the position axis and 14 m in the distance axis, potentially yielding higher errors.

Next, we examined whether the distance-by-position representation enables decoding simultaneously the interbat distance and the position of the bat during cross-overs. We were able to simultaneously decode both variables using relatively small cell numbers (Fig. 4e,f and Extended Data Fig. 13; *n* = 10–22 simultaneously recorded cells; Methods). The interbat distance was decoded above chance at all ±40 m distances (Fig. 4f (right) and Extended Data Fig. 13b), although decoding was better at short interbat distances of ±20 m. Interestingly, when dividing the cross-over data to attention levels on the basis of the bat’s echolocation click rate, we found that the distance decoding error decreased as the click rate increased, that is, as the attention increased (Fig. 4g,h and Extended Data Fig. 13c,d). This suggests that, on a trial-by-trial basis, hippocampal neurons encode distance information more precisely when the recorded bat is more attentive to the other bat.

## Coding of distance by position is non-separable

**Fig. 5:**
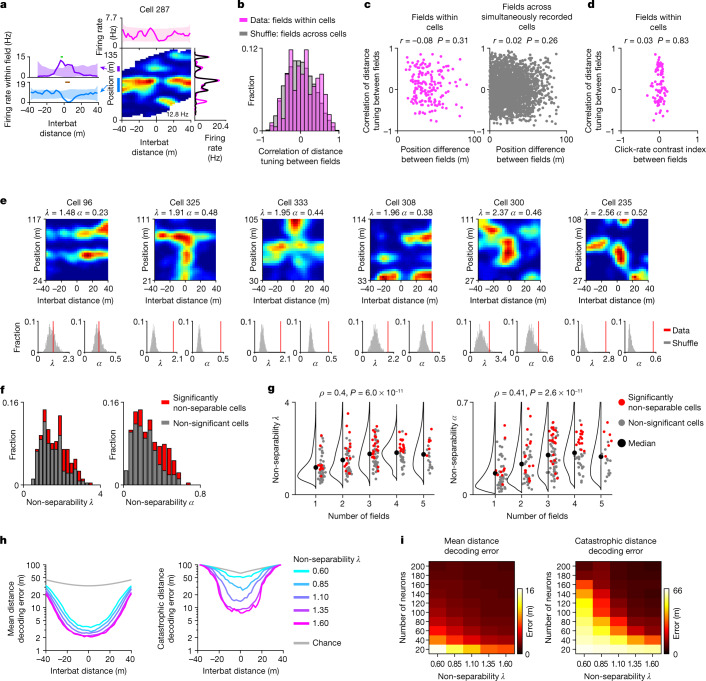
The representation of distance by position is complex and non-separable. **a**–**d**, Different distance tuning across different place fields. **a**, Example cell. Note the two place fields showed opposite distance modulation. **b**, The distribution of Pearson correlations between the 1D distance tuning curves of pairs of place fields of the same cell (pink; *n* = 170 pairs; the inclusion criteria are described in the Methods), or place fields of different simultaneously recorded cells (grey, cell shuffling; *n* *=* 2,479 pairs). **c**, Pearson correlations between the 1D distance tuning curves of pairs of place fields of the same cell (left) or of simultaneously recorded different cells (right), plotted versus the position difference between the place-field peaks (Pearson correlations of the scatters are indicated). **d**, Pearson correlations between the 1D distance tuning curves of pairs of place fields of the same cell (*y* axis) and the contrast index of echolocation click rate during cross-over within these pairs of place fields (*x* axis; contrast index = (CR_field1_ − CR_field2_) / (CR_field1_ + CR_field2_), where CR is the click rate). *n* *=* 79 pairs of place fields recorded with audio. **e**–**g**, SVD analysis shows non-separable distance-by-position coding. **e**, Example neurons with varying degrees of non-separability. Top, 2D firing rate map of position (*y* axis) by interbat distance (*x* axis), cropped and completed for the SVD analysis (Methods); *λ* and *α* (non-separability indices) are indicated: higher values denote non-separable cells; neurons are sorted by *λ*. Bottom, histograms of shuffle distributions (grey) and real values (red lines) for *λ* (left) and *α* (right). Cell 96 is separable (two place fields show same distance tuning), the other five cells are significantly non-separable (different distance modulations at different positions). **f**, The distributions of *λ* and *α* across all cells in the SVD analysis (*n* = 262 cells × directions; inclusion criteria are described in the Methods). Grey, non-significant cells (*n* = 189 cells × directions). Red, significantly non-separable cells (*n* = 73 cells × directions). **g**, Non-separability indices *λ* and *α* versus the number of place fields of the cell (only place fields within the SVD rectangular maps were included; *n* = 237 place cells × directions). Spearman correlation *ρ* values are indicated. The black dots show the median; and the black lines are kernel density plots. **h**,**i**, The functional advantage of non-separability: theoretical analysis of distance decoding for simulated neurons. **h**, Mean decoding error (left) and catastrophic decoding error (right, 99th percentile of errors), plotted on a log scale as a function of the interbat distance, separately for simulated populations of cells with different non-separability values, *λ* (different colours). Grey, chance-level decoding. **i**, Mean decoding error (left, colour-coded) and catastrophic decoding error (right) as a function of the number of neurons used for decoding and the non-separability index, *λ*. Source data

We next examined in more detail the nature of the conjunctive 2D distance-by-position coding. In many place cells, different place fields of the same neuron were modulated differently by the interbat distance (Figs. 3a (cell 331) and 5a). Indeed, over the population of place cells, the distance-tuning correlations between place fields of the same neuron were widely distributed around zero (Fig. 5b (pink)), and were only marginally different from the across-cell shuffle distribution (Fig. 5b (grey); there was a slight over-representation of cells with high correlations; Kolmogorov–Smirnov test, *P* = 0.016). Consistent with this, the correlation of distance tuning between pairs of place fields was independent of the difference in their positions along the tunnel (Fig. 5c (left); Pearson *r* = −0.08, *P* = 0.31). This lack of correlation between different fields could not be explained by differences in behaviour along the tunnel because (1) both echolocation profiles and flight velocity were nearly constant along the tunnel (Fig. 1f and Extended Data Fig. 6c); (2) there was no correlation between the difference (contrast index) of the echolocation click rate within pairs of place fields of the same neuron, and the distance-tuning correlations of these fields (Fig. 5d); and (3) simultaneously recorded neurons often showed very different distance tuning at the same position (Fig. 5c (right); note the wide distribution of correlations for small position difference between fields), despite the same underlying behaviour. Taken together, these results indicate that different place fields of the same neuron exhibited almost independent distance tuning, suggesting a non-separable 2D distance-by-position coding—the 2D coding could not be described by a multiplication of the two 1D marginals, that is, by a multiplication of the tuning curves for distance and for position. This finding may imply modularity, whereby different distance computations are performed at different place fields.

**Extended Data Fig. 14:**
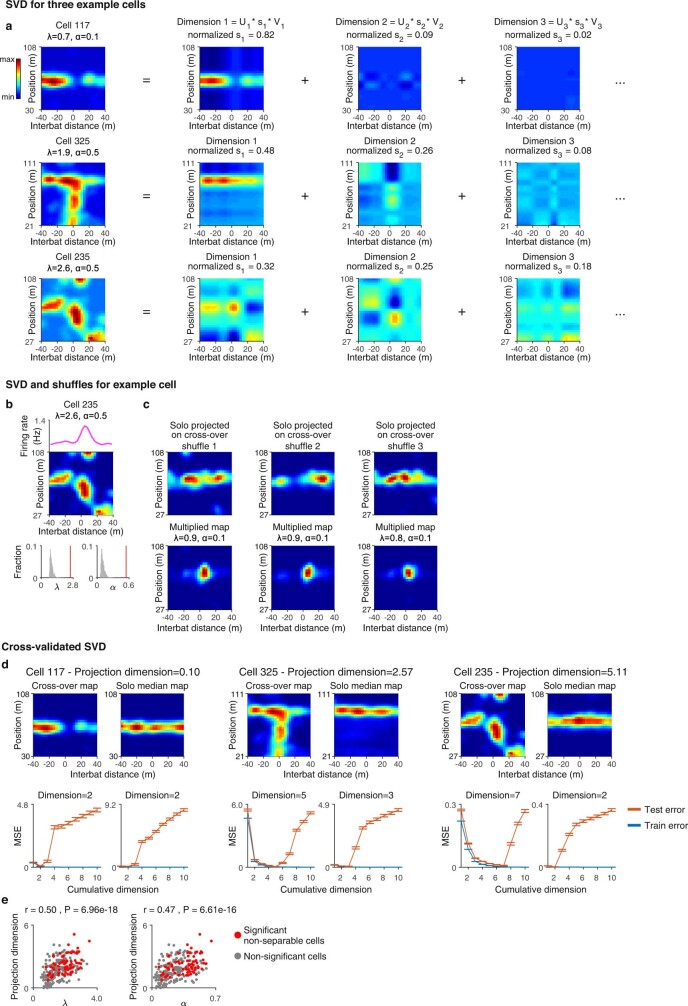
SVD analysis. **a**, Three example neurons (different rows) for SVD analysis (the bottom two cells were also plotted in Fig. 5e). For each cell: left, 2D firing rate map of position (*y*-axis) by interbat distance (*x*-axis), cropped, filled and mean-subtracted to create a full rectangle, as required by the SVD analysis (Methods). Rightmost three panels show the first three matrices reconstructed from the SVD analysis (three first dimensions; see Methods for more details). For each neuron (row), all four maps were set to have the same colour limits (set as the overall minimum and maximum values across the four matrices for that neuron). The singular value of each dimension (s_1_, s_2_ or s_3_) was normalized by the sum of all singular values, and is written above each matrix. For separable cells (such as cell 117), the first dimension captures quite well the 2D firing rate map of the cell, yielding a high singular value, while the second and third dimensions have very low singular values, reflecting their negligible contribution to the reconstructed map (note the nearly-uniform deep-blue colour of the maps for the second and third dimensions in cell 117). By contrast, for non-separable cells (such as cells 325 and 235), the first dimension does not capture well the 2D firing rate map, and therefore adding more dimensions is required: indeed the singular values of the second and sometimes even the third dimensions are not negligible (note the *non*-deep-blue colour of the maps for these dimensions in cells 325 and 235). **b, c**, Example of SVD analysis for cell 235 (this cell is also shown in Fig. 5e right, and in panel **a** above). **b**, Top, 1D distance tuning-curve. Middle, 2D firing rate map of position (*y*-axis) by interbat distance (*x*-axis), cropped and filled to create a full rectangle for the SVD analysis. Bottom, histograms of non-separability indices λ (left) and α (right), calculated for shuffle firing-rate maps (grey, see shuffle examples in panel **c**, bottom); the real values of λ and α for the cell are indicated by a vertical red line – these values were much higher than for the shuffles, indicating significant non-separability for this cell (Methods). **c**, Top, three examples of 2D distance by position firing-rate maps for solo projected on cross-over shuffles – for the cell in panel **b** (see Extended Data Fig. 5 and Methods for the process of generating these types of shuffle matrices). Bottom, same shuffle matrices after multiplying them by the 1D distance tuning of the cell (i.e. by the top pink curve in **b**). These maps at the bottom (‘Multiplied maps’) were the maps used for computing the λ and α values for the shuffle distribution. Note that these shuffle maps are based on the exact same behavioural data, the same spike statistics and the same 1D distance tuning as in the real data – but these shuffle maps are almost separable. Importantly we note that multiplying by the 1D distance tuning did not increase the non-separability, because multiplication is separable by definition – and therefore any non-separability that we would find in these shuffle maps must arise from either the non-uniform coverage of bat-behaviour or from the noisy spiking of the neurons. The λ and α values written above the maps indicate the non-separability indices of these shuffle maps (these are 3 of the 10,000 shuffle values per neuron that are plotted in the grey histograms in **b**-bottom). **d, e**, Cross-validated SVD. **d**, Three example neurons (same neurons as in panel **a**). Top left, firing rate map of position (*y*-axis) by interbat distance (*x*-axis) during cross-over, plotted as in panel **a**. Top right, median map for solo projected on cross-over shuffles (median of all the 10,000 shuffle maps, examples of which are shown in the top row in **c**). Bottom, train errors (blue) and test errors (red) as a function of cumulative dimension, using the cross-validated SVD analysis for the maps above (MSE: mean squared error; see Methods). The dimensionality of each map equals the dimension at which the test error curve (red) reaches its minimum. Then, to compute the effective dimensionality of the cell (‘projection dimension’, denoted in the title of each cell), we projected the median solo singular vectors on the cross-over singular vectors. This procedure captures the dimension of the cross-over maps, after removing any non-separability that might arise from a non-uniform behaviour or noisy spiking statistics (Methods). Cell 117 is a separable cell (i.e., it can be described by multiplication of distance tuning × position tuning), and accordingly its projection dimension is < 1. Cells 325 and 235 are significantly non-separable cells with projection dimension ≥ 1. **e**, Scatter plot of projection dimension computed in the cross-validated SVD analysis, plotted versus λ in the left panel – for all the distance cells that were valid for this analysis (Pearson correlations: all cells: *r* = 0.50, *P* = 6.96 × 10^–18^, *n* = 262 cells × directions; non-significant cells [grey]: *r* = 0.43, *P* = 7.16 × 10^–10^, *n* = 189 cells × directions; significant non-separable cells [red]: *r* = 0.48, *P* = 1.83×10^−5^, *n* = 73 cells × directions); or plotted versus α in the right panel (Pearson correlations: all cells: *r* = 0.47, *P* = 6.61 × 10^–16^, *n* = 262 cells × directions; non-significant cells [grey]: *r* = 0.38, *P* = 5.53 × 10^−8^, *n* = 189 cells × directions; significant non-separable cells [red]: *r* = 0.45, *P* = 5.31 × 10^−5^, *n* = 73 cells × directions). Importantly, we also verified that the non-separability of the cells could not be explained by the quality of the spike-sorting: We found no correlation between the isolation-distance of the cells – a common metric used for quantifying spike-sorting quality^72^ – and the non-separability indices of the SVD analysis (λ: Pearson *r* = 0.09, *P* *=* 0.15, *n* = 262 cells × directions; α: Pearson *r* = 0.08, *P* = 0.18, *n* = 262 cells × directions). Likewise, we found no correlation between the isolation-distance of the cell and the distance tuning correlation between pairs of place fields of the same neuron (as in Fig. 5b–d; Pearson *r* = −0.05, *P* = 0.48, *n* = 170 place-field pairs). Further, the non-separability could not be explained by non-homogeneities in click-rate or speed – because the click-rate modulation profile was uniform along the tunnel (Fig. 1f, Extended Data Fig. 2d), and the speed profile was also uniform along the tunnel (Extended Data Fig. 6c, bottom). Thus, the non-separable coding is a genuine phenomenon.

To further characterize the non-separability of the 2D distance-by-position coding of CA1 neurons, we performed a singular value decomposition (SVD) analysis^31,32^ (Fig. 5e–g, Extended Data Fig. 14 and Methods). This standard analysis enables us to determine whether a 2D map is separable (Fig. 5e (cell 96)) or is more complex (non-separable cells; Fig. 5e (five rightmost cells)). We quantified the non-separability using two SVD indices (*λ* and *α*, where higher values indicate stronger non-separability; Methods), and compared it to shuffles: this revealed that 27.9% of the pyramidal cells tuned to distance were significantly non-separable (Fig. 5f). Dimensionality analysis showed that some of the non-separable cells required more than four dimensions to describe their 2D distance-by-position maps (Extended Data Fig. 14d,e; mean, 2.25 dimensions), highlighting the complexity of their 2D tuning. This non-separability could not be explained by spike-sorting quality, nor by the very small inhomogeneities in click rate or flight speed (details are provided in the legend of Extended Data Fig. 14) (Fig. 1f and Extended Data Fig. 6c). Moreover, we found that place cells with a higher number of place fields were more likely to have higher non-separability indices (Fig. 5g).

**Extended Data Fig. 15:**
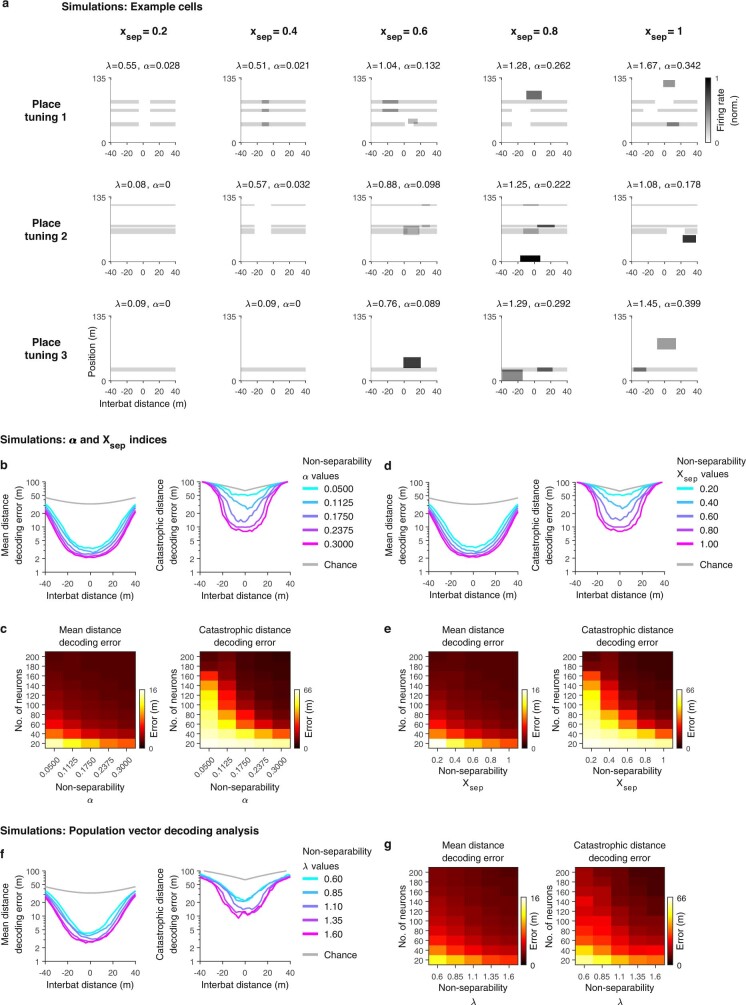
Functional advantage of non-separability: theoretical decoding analysis. **a**, Fifteen examples of simulated cells, showing 2D maps of distance by position for 3 different underlying position tunings (3 rows) and 5 different levels of non-separability (5 columns). For each position tuning (each row), we created different distance modulations using 5 values of the non-separability parameter, *x*_sep_ (Methods): low *x*_sep_ generates separable 2D maps, while larger *x*_sep_ generates non-separable maps with higher values of non-separability indices λ and α, computed as in the SVD analysis; *x*_sep_, λ, α are indicated for each map. **b–e**, Results of the maximum likelihood decoder. **b, d**, Mean distance decoding error (left) and catastrophic distance decoding error (right, 99% percentile of the errors), plotted in log-scale as a function of the interbat distance, separately for simulated populations of cells with different non-separability values (α values used in panel **b**, and *x*_sep_ values used in panel **d**). Note that as the population of cells becomes more non-separable (higher α or higher *x*_sep_) the decoding error decreases. **c, e**, Mean distance decoding error (left, colour-coded) and catastrophic distance decoding error (right) as a function of the number of neurons used for decoding and the non-separability index (α values used in panel **c**, and *x*_sep_ values used in panel **e**). Note that in these four matrices, increasing the non-separability has a similar effect on error-reduction as adding more neurons. **f, g**, Similar plots to main Fig. 5h, i, but here we used population vector decoding instead of maximum likelihood decoding. Both types of decoders yielded very similar results: As the population of cells became more non-separable, the decoding error decreased.

Although non-separable complex tuning has been found also in other brain areas^31–36^, it remained unclear whether such tuning offers any functional advantage. A previous theoretical study showed that conjunctive 2D representation of two variables is more efficient than two separate 1D representations (by separate neuronal populations), especially when fast computations are needed for the two variables simultaneously^37^. However, that study did not examine specifically whether a non-separable conjunctive 2D representation is useful. To investigate the possible advantage of such non-separable coding in the hippocampus, we simulated populations of place cells with the same distance modulations but with different degrees of non-separability (Extended Data Fig. 15a). We then decoded the interbat distance from each population, and found that higher levels of non-separability led to lower decoding errors (Fig. 5h and Extended Data Fig. 15b,d,f). Furthermore, increasing the non-separability of the cells was equivalent to increasing the population size—that is, the same decoding error can be achieved with fewer neurons if their tuning is non-separable (Fig. 5i and Extended Data Fig. 15c,e,g). This suggests that the non-separable distance-by-position tuning yields a more efficient encoding of distance.

## Discussion

Here we studied naturalistic behavioural switches between navigation and collision avoidance in flying bats, and found three key results: (1) more than half of the hippocampal place cells encoded conjunctively distance-by-position information (Fig. 3)—switching very rapidly (as fast as 100 ms) from a position representation to a distance-by-position representation (Fig. 3c and Extended Data Fig. 11e). Crucially, these rapid switches occurred at the level of single neurons. (2) The distance tuning was modulated by the bat’s sonar-guided attention (Figs. 2e–g and  4g,h and Extended Data Fig. 13c,d). (3) Finally, we found that, for many cells, the distance-by-position tuning was non-separable—an individual neuron could exhibit different distance modulation at different positions along the tunnel; specifically, different place fields of the same neuron exhibited very different distance tuning (Figs. 3a and 5). This may suggest modularity of hippocampal processing across different place fields. Simulations of a theoretical model showed that such non-separable neuronal code leads to better encoding of distance information (Fig. 5h,i). Our results are fundamentally distinct from previous reports (for details, see the ‘Additional discussion’ section in the Methods).

**Extended Data Fig. 16:**
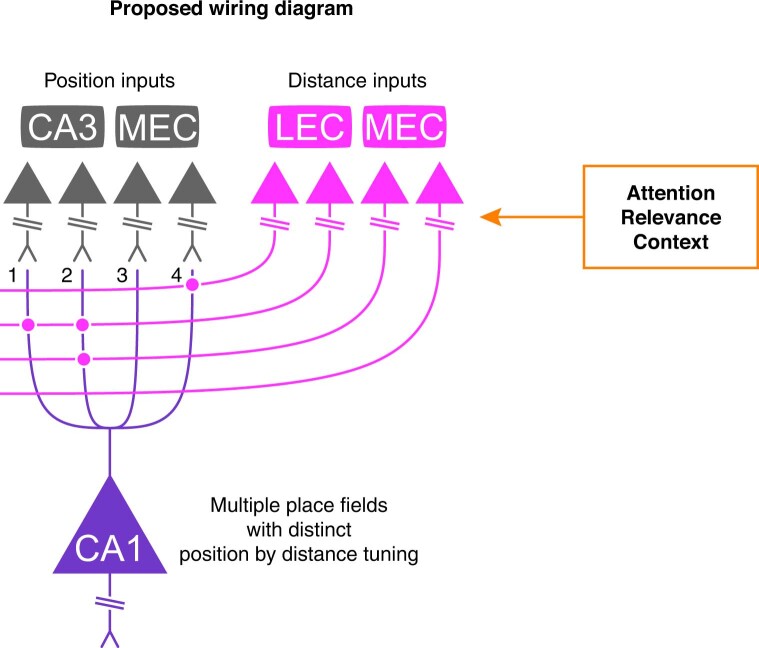
Proposed wiring diagram for explaining the non-separable distance by position coding. Cartoon of a CA1 neuron. Multiple independent position inputs (grey) arrive from neurons with single place fields in hippocampal area CA3, as was suggested in ref. ^14^, and impinge on different dendrites of a CA1 neuron – forming a place cell with multiple place fields. These position inputs could also originate from medial entorhinal cortex (MEC)^73^. In addition, we propose that independent diverse distance inputs (pink) arrive to the dendrites of the same CA1 neuron. The result of such convergence between independent distance and position inputs can create a non-separable CA1 neuron with multiple place fields, each with a different distance tuning in each place-field – as we observed in many neurons in our data (Fig. 3a, Fig. 5a–d). It can also create separable neurons (see below). Several comments are noteworthy here. (1) First, the distance inputs could arrive possibly from lateral entorhinal cortex, LEC, where egocentric coding was reported^20^, or from the subiculum via the medial entorhinal cortex (MEC)^18,21,23^. These LEC/MEC inputs might activate pyramidal CA1 neurons either directly, or disynaptically through CA1 interneurons^74^. We note that the distance information can arrive to LEC/MEC from either visual areas (as these bats are highly-visual^29^), or it can reflect echolocation-based sensory information about the distance from the other bat^75,76^. An alternative model posits that since MEC and LEC inputs converge anatomically already in CA3, upstream of CA1^77^, it is also possible that the distance by position tuning might be found already in CA3 – and is inherited from CA3 by the CA1 neurons. Both options could explain the non-separability of the 2D distance-by-position maps found in CA1. Future experiments will be needed in order to test these possibilities. (2) Second, this schematic wiring-diagram suggests that not only a CA1 neuron as a whole can be conjunctively tuned to distance by position, but also that each of its single dendrites might be conjunctively tuned to an independent combination of distance by position. In other words, each dendrite may serve as a complex processing-stage – a possibility that is supported by the literature on dendritic computations^78–81^, but will need to be tested directly in future experiments. Consistent with this, we found that most place-field pairs within the same cell in the data exhibited low correlations between their distance tunings; only a minority showed high positive correlations, which may reflect a common distance input to both fields, i.e. to both dendrites (Fig. 5b, small over-representation of positive high correlations). To account for this possibility, the wiring diagram here shows also that two dendrites of the same neuron can sometimes receive distance input from the same neuron in LEC or MEC (see distance input to dendrites no. 1 and 2 in the schematic). An alternative explanation for this minority of cells with high correlations (seen in Fig. 5b) might be that the LEC or MEC itself carries distance by position information; however, the position information in LEC was reported to be very weak^82^, and distance tuning *per se* was not reported to date in MEC – so this option seems less likely. Therefore, we believe that our schematic wiring diagram is more probable. (3) Third, the proposed schematic model could also explain how sub-threshold position fields, which are not defined as place fields, are enhanced during cross-overs (Fig. 3d, Extended Data Fig. 12: distance tuning inside “interfields”): This can occur via summation of a sub-threshold position input and sub-threshold distance input, which together cross the firing-threshold. (4) Fourth and finally, we note that in this model we posit also external inputs that carry attention, relevance, or context signals (see rectangle on the right). These inputs could explain, for example, why distance tuning is observed during cross-overs, when it is highly relevant, but not during tracking (Extended Data Fig. 9). These hypothesized attention / relevance / context inputs may arrive directly to CA1, or via LEC or MEC – both options may explain the attentional modulation of the 2D distance-by-position coding that we observed in CA1.

We propose that the non-separable code could be formed by independent position and distance inputs arriving to CA1 (Extended Data Fig. 16). Specifically, we previously proposed that the multiple place fields of a single CA1 neuron arise from different dendrites receiving independent position inputs^14^ (Extended Data Fig. 16 (grey); inputs from CA3 or medial entorhinal cortex). Here we speculate that there are also independent distance inputs, with a variety of different distance modulation profiles, that arrive at CA1 (for example, from the lateral entorhinal cortex or from the subiculum through the medial entorhinal cortex), as well as inputs that carry attention or context signals (Extended Data Fig. 16). These converging streams are combined in CA1, generating multiple place fields with different distance modulations, similar to what we found experimentally. This model suggests a key computational role for dendrites in generating the complex distance-by-position tuning observed in CA1.

Overall, our results suggest that, during a natural behavioural switch, the same hippocampal neurons can switch between two different types of neural codes very rapidly and flexibly, reflecting the animal’s behavioural needs. This dynamic view of hippocampal function calls for future dynamic experiments designed to further elucidate how the hippocampus supports flexible natural behaviours.

## Methods

### Bats and behavioural set-up

#### Bats

Four adult male Egyptian fruit bats, *Rousettus aegyptiacus*, were included in this study for electrophysiological recordings (weight, 160–175 g). No randomization or blinding was applied in this study. Sample size was not predetermined; the number of animals is standard for studies in this research field. Information on the individual bats is summarized in Extended Data Table 1. An additional four bats were used for the behavioural assay as partners. All of the bats in this paper were caught as adults in the wild (in Israel). All experimental procedures were approved by the Institutional Animal Care and Use Committee of the Weizmann Institute of Science.

#### Set-up

Bats flew in pairs (two bats together in each experiment) in a long straight tunnel (135 m × 2.3 m × 2.35 m (length, width, height); Fig. 1a). This flight tunnel was part of a longer 200 m tunnel, and the 135 m straight part was blocked by an opaque curtain. The bats flew between two landing balls that were located at the two ends of the tunnel, at a distance of 125 m from each other. On these balls, the bats could rest and receive food. The tunnel was uniformly illuminated (illuminance level: 5 lux). To ensure that the bats were aware of their position, 10 unique landmarks were distributed randomly along the tunnel, and were positioned at fixed locations across all of the experiments.

#### Training

The bats were initially pretrained for ~2 weeks (15–20 min per day) to fly alone between two landing balls in small-scale environments (either a flight room of ~6 × 5 m, or a short ~6 m segment of the flight tunnel), with the aim of getting used to handling by humans, and learning to directly fly between the two landing balls. The bats were then introduced individually to the long tunnel (135 m) with three training aims: first, getting the bats used to the experimental set-up; second, enhancing flight stamina; and third, training them to fly direct flights between the landing balls without performing U-turns. Once they reached good flight performance (which took around 1–2 weeks), they were introduced to their partner and flew together in the tunnel for about 1 week before surgery.

After training, the bats were implanted with a microdrive for electrophysiological recordings in the dorsal hippocampal area CA1 (see below). Each experimental session started and ended with sleep sessions (each sleep session lasting 5–10 min). For the sleep sessions, the bat was placed alone inside a small covered cage which was positioned on the floor in a quiet location inside the tunnel.

#### Behavioural paradigm

A pair of bats flew together in the tunnel, performing direct flights between two landing balls. We considered two different behavioural modes (Fig. 1a,b and Extended Data Fig. 1): (1) Solo flights, in which one bat was flying alone and the other bat was resting, or when the distance between the bats was >40 m. (2) Cross-over flights, in which the two bats flew towards each other with an interbat distance of within ±40 m (for more details, see the ‘Extracting flights and dividing data to different behavioural modes’ section below). During cross-overs, the bats had to be attentive to avoid collision with the other bat as this is a relatively narrow tunnel and because the bats crossed each other at a very high combined relative speed of around 14 m s^−1^ (see Extended Data Table 1 for the speeds of individual bats). Although collision avoidance is a very natural behaviour for bats, it was by no means trivial—we actually had a few collisions or near-collisions between the bats during training, as well as during the experiments themselves (Extended Data Fig. 2g), emphasizing that the need to pay attention was not hypothetical.

The experimenters timed the bats’ take-offs from the two balls at both ends of the tunnel to create a nearly uniform coverage of cross-overs along the tunnel, which was roughly randomly distributed over time (Fig. 1c and Extended Data Fig. 1). The experimenters stood near to the landing balls at the two ends of the tunnel, and did not enter the central 125 m behavioural zone. The bats flew in total 13.14 ± 3.50 km per session (mean ± s.d.; the distance per bat is shown in Extended Data Table 1); the behavioural session lasted around 1.5–2 h.

For one pair of bats, we had two additional behavioural modes (recorded bat no. 30; Extended Data Table 1). (1) Tracking mode: the two bats flew in the same direction with a short interbat distance (one bat tracked the other with an interbat distance of less than ±20 m; Extended Data Fig. 9). (2) Obstacle-avoidance mode: in the middle of the session, we introduced a stationary obstacle (vertical pole) inside the tunnel, which the bats had to avoid colliding with. As we decided to focus here on cross-over behaviours with other bats, we removed epochs near to this stationary obstacle for those cells that showed significant modulation by the obstacle (this resulted in the removal of 0.49% of the flight data for the cells recorded with obstacle).

In another bat pair, we conducted an additional session in which we switched the partner bat. In the first session, the recorded bat (bat no. 2299) was flying with his usual partner for a full session of 1.5–2 h (session a), then it rested ~40–60 min and we then ran the experiment again with an alternative partner for another ~1 h (session b). The recorded bat was familiar with both the usual partner and the alternative partner before surgery. As this was a very long and physically demanding experiment for the recorded bat (the recorded bat flew 21.85 ± 2.07 km per day in these two sessions together (mean ± s.d.), while carrying the recording-devices on its head), we succeeded to run this two-session experiment for only 3 days. A comparison of neural recordings from these two sessions is shown in Extended Data Fig. 10.

The use of a long flight tunnel in our experiment was crucial for several reasons: (1) in such a large-scale set-up, the bats fly very fast, which enabled us to investigate hippocampal neural activity during fast behavioural switches. (2) As we knew that the bats respond to each other at a distance of approximately −20 m before cross-over (Fig. 1d,e), and as we wanted to record also baseline activity before that (for comparison), this required defining a large symmetric window of ±40 m as the cross-over flight, which necessitated a large-scale environment. (3) To examine the response to interbat distance irrespective of position, we needed to disentangle distance from position in the experiment, which required having cross-over events at different locations in the tunnel (Fig. 1c and Extended Data Fig. 1). This required a long tunnel. (4) As in large-scale environments there are multiple place fields for each place cell^14^, it enabled us to test whether different place fields of the same neuron exhibit different distance tuning (Fig. 5). This would not have been possible in small environments, in which typically only one place field is observed for each place cell^14^.

### Animal localization system

We tracked the position of the bats using wireless radio-frequency localization tags (weight, 6.6 g, including battery; BeSpoon), which received and transmitted signals to an array of 14 ground-based antennas that were distributed around the tunnel. A spherical estimation of the tag’s distance from each antenna was computed on the basis of the time interval between the signal transmission and arrival. The position of the bat in 3D was then estimated using the intersection of these spheres from all of the antennas. This localization method yielded a good precision of around 9 cm in the longitudinal and lateral axes of the tunnel^14^, but the vertical precision was poorer, and we therefore did not use the height measurements for analysis. Each bat had his own tag with a unique ID; the position of each bat was computed at a sample rate of 12.8 Hz or 16 Hz. Both tags were synchronized to the neural recordings using a non-periodic sequence of TTL pulses (precision of <1 ms).

### Extracting flights and dividing data to different behavioural modes

All data in this study were analysed using MATLAB. Location data from the localization system were processed as described in ref. ^14^. In brief, we first removed outliers (defined as data points that were far away (>2 m) perpendicularly from the tunnel’s midline, or data with unreasonably high speed (>20 m s^−1^)). We then linearized the data by projecting the valid positional data onto the long 1D axis of the tunnel. We then filled short gaps where data were missing, as described in ref. ^14^. Finally, the positional data were upsampled to 100 Hz.

We analysed only flight epochs, and excluded data from take-offs and landings: flight epochs were detected based on: (1) speed (>2 m s^−1^), and (2) distance from the landing balls (>3 m from the balls). In the cases in which the bat performed a U-turn in the middle of the tunnel, we discarded an extra 3 m before and after the U-turn event (beyond the 2 m s^−1^ speed threshold) to avoid contamination by possible ‘U-turn coding’.

We further divided the data to distinct behavioural modes on the basis of the localization data of the two bats and their flight direction:

1. Solo flights: the recorded bat was flying while the other bat was resting, or both bats were flying and the interbat distance was >40 m. The 40 m threshold was taken because bats are probably not attentive to the other bat at large distances of >40 m (see the next paragraph), and we therefore considered such large distances as if the bat was flying alone. Short solo flights that lasted less than 2 s (corresponding to around 14 m) were discarded from the analysis.
2. Cross-overs: the bats flew in opposite directions and passed each other. The cross-over event was defined as the point of interbat distance = 0, with the distance measured along the long axis of the tunnel. As cross-overs are momentary events, we considered a distance-window of ±40 m around each cross-over event, which we defined as a cross-over flight. The bats increased their echolocation rate at distances between −20 m and 0 m (Fig. 1d–f and Extended Data Fig. 2), which suggests that they are attending to the other bat at these distances^1–9,38–41^; to enable the analysis of an extra baseline, we considered a larger and symmetrical distance window of ±40 m, which enabled us to detect neuronal modulations and their return to baseline activity. Cross-overs that occurred less than 8 m from the landing balls were excluded from the analysis to avoid effects of landing and take-off (see the light grey vertical rectangles in Fig. 1c and Extended Data Fig. 1b,e). Furthermore, we excluded cross-over flights in which the two bats did not fly in opposite directions for at least 5 m before and 5 m after the cross-over event. Most of the valid cross-over flights were long (median, 80 m, which is also the maximum possible length of a cross-over flight, given our ±40 m analysis window); however, cross-over events that were close to landing balls or U-turns resulted in cross-over flights that were shorter than 80 m (mean cross-over flight length: 74.84 ± 11.36 m (mean ± s.d.); minimal length 20.77 m).
3. Tracking: the two bats flew in the same direction with an interbat distance of less than ±20 m. We further divided the tracking epochs to flights in which the recorded bat was following the other bat (interbat distance between −20 m and 0 m) and when the recoded bat was leading (interbat distance between 0 m and 20 m). Tracking behaviour was analysed only in bat no. 30, which was trained to perform many tracking flights: these analyses are shown in Extended Data Fig. 9. In all of the other bats we had only a few or no tracking flights during the session, and they were therefore not analysed.

### Audio recording and click detection

#### Audio recording

In Egyptian fruit bats, echolocation consists of pairs of very short ultrasonic clicks^5,6^. To detect these clicks, we recorded the audio signal using an on-board audio logger with an ultrasonic microphone. As there is a limit to the weight that bats can carry, in most experiments we could not record simultaneously the audio signal (audio logger) together with the neuronal recordings (neural logger) and position (positioning tag). Thus, in two bat pairs (recorded bat–partner no.: 2336–2331 and 2389–2387) we recorded the audio signal separately in some of the days instead of recording neuronal data. For bat 30, audio was recorded using a different audio-logger device and therefore its click amplitude was not comparable with the other bats and was not analysed in Extended Data Fig. 2c (the click rate from bat 30 appears only in Extended Data Fig. 9b). In our last bat pair (recorded bat–partner no.: 2299–2331), a new miniature version of the data logger was developed, which enabled us to record simultaneously both the neuronal activity and the audio signal. Together, behavioural analyses of the bats’ echolocation were performed on *n* = 6 bats (Extended Data Figs. 2c and 9b), and analyses combing simultaneously recorded neural data and echolocation data were performed on one bat (bat no. 2299; Figs. 2e–g and 4g,h and Extended Data Figs. 7 and 13c,d). For all of the audio recordings, with all devices, audio signals were filtered online (in hardware) between 4–40 kHz, and were recorded at a 100 kHz sample rate (this frequency range covers most of the energy of the echolocation clicks of this bat species^6^).

#### Click detection

Detection of echolocation clicks emitted during flight was performed offline as follows: we first further high-pass-filtered the audio signal at 10 kHz, as the echolocation clicks of this species do not contain energy below 10 kHz^6^. We then normalized the signal by its mean absolute deviation (MAD) over the entire session—transforming the amplitude to a SNR. We then used an amplitude threshold of 50 MADs (that is, SNR = 50), which detected clicks reliably, together with several time-domain criteria on the basis of known properties of the sonar clicks of this bat species^6^: (1) duration of click of 30–2,500 μs; (2) maximum rise time of 500 μs; and (3) minimum inter-click interval of 10 ms. We also used a frequency criterion, consisting of a minimum energy ratio of 10 dB between a high-frequency band (18–40 kHz) and a low-frequency band (5–12 kHz). As we recorded audio signals from both bats, we could remove clicks originating from the other bat by estimating their expected time-of-arrival to the recorded bat’s microphone using the interbat distance (measured using the localization system) divided by the speed of sound: this enabled us to discard low-amplitude clicks (SNR = 50–200) emitted by the other bat, which were detected on the recorded bat’s microphone at these estimated timings ±3 ms (Extended Data Fig. 2a). In the bat with simultaneous audio and neural recordings, we also manually curated the detected clicks to further improve the click detection (1.77% manually added clicks, 0.01% removed clicks).

### Surgery and neural recording techniques

All of the surgical procedures were performed as described previously^14^. In brief, after completion of training, bats were implanted with either a 4-tetrode microdrive (weight, 2.1 g; Neuralynx), or a 16-tetrode microdrive (weight, 3.4 g; modified from ref. ^42^), loaded with tetrodes, with each tetrode constructed from four strands of insulated wire (17.8 μm diameter platinum-iridium wire). Tetrodes were gold-plated to reduce the wire impedance to 0.3 MΩ (at 1 kHz). The microdrive was implanted above the right dorsal hippocampus (3.0–3.6 mm lateral to the midline and 5.8 mm anterior to the transverse sinus that runs between the posterior part of the cortex and the cerebellum); the craniotomy was then covered with an inert silicone elastomer (Kwik-Sil or Kwik-Cast). During the implantation surgery, we used an injectable anaesthesia cocktail composed of medetomidine (0.25 mg kg^−1^), midazolam (2.5 mg kg^−1^) and fentanyl (0.025 mg kg^−1^), and added supplemental injections as needed, based on the bat’s breathing and heart-rate^43^. The microdrive was attached to the skull with bone screws, using a layer of adhesive (Super-Bond C&B) followed by dental acrylic. We attached the ground wire from the microdrive to a bone-screw that touched the dura in the skull’s frontal plate.

After surgery, the tetrodes were slowly lowered towards the CA1 pyramidal layer; positioning of tetrodes in the layer was provisionally performed on the basis of the presence of high-frequency field oscillations (ripples) and associated neuronal firing, and was later verified histologically (Extended Data Fig. 3a). For each bat, one tetrode was left in an electrically quiet zone and served as a reference, and the remaining tetrodes served as recording probes.

During recordings, a wireless neural-recording device (neural-logger; 16-channels or 64-channels, Deuteron Technologies) was attached to a connector on the microdrive. Signals from all channels were amplified (×200) and bandpass filtered (1–7,000 Hz), and were then sampled continuously at 31.25 or 32 kHz per channel, and stored on board the neural-logger. During subsequent processing, the neural recording was further high-pass filtered with a 600 Hz cut-off for spikes, creating a spike bandwidth of 600–7,000 Hz, and then a voltage threshold was used for extracting 1 ms spike waveforms.

### Histology

Histology was performed as described previously^14^. In brief, at the end of recordings, the bats were anaesthetized, and electrolytic lesions (DC positive current of 30 μA, 15-s duration) were made to assist in the precise reconstruction of tetrode positions. The bat was then given an overdose of sodium pentobarbital and, with tetrodes left in situ, was perfused transcardially using 4% paraformaldehyde or 4.5% Histofix. The brain was removed and thin coronal sections were cut at 30 μm intervals. The sections were Nissl-stained with cresyl violet and were photographed to determine the locations of tetrode tracks in the dorsal CA1 (Extended Data Fig. 3a). Recording sites were all located in the dorsal CA1 (except for one tetrode from one bat, which was possibly placed in CA2, that was also included in the dataset), and spanned the entire extent of the proximo-distal axis of CA1.

### Spike sorting

Spike-sorting procedures were similar to those described previously^13,14^. In brief, spike waveforms were sorted manually using Plexon Offline Sorter, on the basis of their relative amplitudes on different channels of each tetrode. Data from all the behavioural sessions and sleep sessions from the same recording day were spike-sorted together. Well-isolated clusters of spikes were manually selected, and a refractory period (<2 ms) in the interspike-interval histogram was verified. Spike sorting was performed in consecutive time windows to allow for drift correction of the spike clusters.

In total, we recorded 499 well-isolated CA1 neurons from 4 bats. We further analysed only 430 neurons that were valid for analysis (see the inclusion-criteria below). Out of the 430 valid neurons, we detected 389 putative pyramidal neurons (90.5% of the cells; based on average firing rate <5 Hz during the entire recording session), and 41 putative interneurons (9.5%; firing rate ≥5 Hz). As hippocampal place cells are known to remap between movement directions in a linear track^14,44,45^ (Extended Data Fig. 3g), we separated all analyses into flight directions and considered each direction independently. Cells × directions were defined as valid for analysis on the basis of the following behavioural and neuronal inclusion criteria: (1) 10 or more cross-overs per flight direction; and (2) or (3), where: (2) a minimum of 50 spikes per direction during solo flights; (3) a minimum of 30 spikes per direction during cross-over flights. This resulted in 693 pyramidal cells × directions and 74 interneurons × directions that were valid for analysis in this paper.

### Statistics

Unless otherwise noted, for all the pairwise comparisons, we used two-tailed (two-sided) statistical tests, with a probability threshold of *P* = 0.05. All correlations were based on a two-tailed Pearson’s correlation coefficient (except for a few cases in which we used non-parametric Spearman correlations, if the scatters were clearly non-Gaussian). We used two-sample Kolmogorov–Smirnov tests to compare distribution shapes. To determine the significance of place tuning and of distance tuning, we compared the real data with shuffled data (see below). When plotting shuffle tuning curves for neurons, we always plotted 5–95% of the shuffle tuning curves.

### Position tuning, field detection and place cells classification

#### Position tuning

As explained above, we performed all analyses in this Article separately for the two flight directions. Tuning curves for 1D position (place tuning) were computed by counting the number of spikes and the time spent in each spatial bin (0.5 m bins). Bins with a time spent of <0.8 s were discarded from the analysis (corresponding to around 11 flights passing through that bin). Spike-count maps and time-spent maps were then smoothed (Gaussian kernel *σ* = 3 bins = 1.5 m), and we then divided, bin by bin, the smoothed 1D spike-count map by the smoothed 1D time-spent map. We computed the spatial information (SI) as follows: ${\rm{SI}}\left(\frac{{\rm{bits}}}{{\rm{spike}}}\right)=\sum _{{\rm{i}}}{p}_{i}\left(\frac{{r}_{i}}{\bar{r}}\right){\log }_{2}\left(\frac{{r}_{i}}{\bar{r}}\right)$, where *r*_*i*_ is the firing rate of the cell in the *i*th bin, *p*_*i*_ is the probability of the bat to be in this bin and $\bar{r}$ is the mean firing rate of the cell.

#### Place cell classification

Place cells were classified using data from solo flights on the basis of the following inclusion criteria: (1) significant spatial information compared to shuffle (>99% of the shuffles): to shuffle the spike train, we rigidly and circularly shifted in time the spikes of each flight, using a uniform random shift; the value of the shift differed randomly between individual flights, so each shuffle consisted of a unique set of temporal shifts across the set of flights. We performed 1,000 such shuffles. (2) Spatial information was >0.25 bits per spike. (3) The cell emitted ≥50 spikes during solo flights. (4) The cell had at least one significant place field, as described next.

#### Place field detection

Place fields were detected during solo flights similarly to our previous study^14^: (1) First, we extracted local peaks in the firing rate map, with a peak rate of >0.5 Hz. (2) To remove small local peaks ‘riding’ on a large field, we searched for shallow ‘dips’, that is, cases in which the dip between two adjacent peaks was >50% of the firing rate of the larger peak, and then disregarded the lower peak. (3) We next defined the field width as the zone in which the firing rate was ≥20% of the peak firing rate of that place field. (4) Field stability criterion: we required at least five different laps with spikes to have occurred inside the place field, or 20% of the laps with spikes—whichever is larger. (5) Field significance criterion: to capture clear distinct fields, we treated a place field as significant only if it had significant spatial information in its local area, near the place field. To quantify this, we focused on the local area surrounding the place field (the width of the field itself plus 50% of the field’s width in each direction), and calculated the spatial information in this local area for the real spikes, and also for 1,000 shuffles (same rigid shuffling of spikes as above). We considered the field to be significant only if it had spatial information of >95% of the shuffles in the same local area.

### 1D distance tuning and classification of 1D distance cells

#### Distance tuning

Tuning curves for the 1D distance between the bats were computed by counting the number of spikes in each distance bin (2 m bins), and dividing it by the time spent in each bin. Bins with a time spent of <0.4 s were discarded from the analysis. The tuning curves were next smoothed (rectangular window of 3 bins). Note that the 1D interbat distance along the tunnel axis was highly correlated with the Euclidean distance between the bats and with the time to cross-over (Extended Data Fig. 4d–g; mean Pearson correlation: *r* = 0.99999 and *r* = 0.9987, respectively), so any of these variables could be used to analyse the data; for consistency with the 1D position coding, we focused on the 1D interbat distance along the tunnel.

#### Shuffles for distance tuning

For classifying neurons as 1D distance cells, we computed two types of shuffles, and required the neuron to be significant according to both types of shuffles: (1) cross-over data shuffle: this shuffle was aimed to test whether during cross-overs the neuron showed enhanced or suppressed distance responses relative to the cross-over data. We performed 10,000 rigid spike shuffles for the flight data, as we did for the place cell classification above, but here used the data from the cross-over flights. (2) Solo projected on cross-over shuffle: owing to the prominent position coding in the hippocampus, this shuffle was aimed to test whether during cross-overs the neuron showed enhanced or suppressed distance-responses relative to the expected response from the solo data. We performed 10,000 shuffles in which we projected the solo spikes onto the cross-over behavioural data as follows (illustrated in Extended Data Fig. 5a). For each cross-over flight, we pooled all of the solo flights that occurred within the same position in the tunnel and, for each shuffle, we randomly picked one solo flight out of this pool (Extended Data Fig. 5a (i and ii)). We then computed what should be the projected interbat distance of each spike that occurred during this solo flight on the basis of the cross-over flight data (using linear interpolation; Extended Data Fig. 5a (ii and iii)). This yielded 2D distance by position shuffle datasets of spikes that occurred during solo, projected onto the cross-over behaviour (Extended Data Fig. 5a (iv)). This entire process was repeated 10,000 times, creating nearly unique shuffle datasets (across cells, 99.99% of shuffles were based on unique sets of solo flights). This shuffle conserves the spiking statistics of the neuron and its position tuning. This shuffling procedure reflects the null hypothesis that the expected firing pattern of the neuron is driven only by the position along the tunnel, without exhibiting any tuning to interbat distance.

#### Significant bins

To detect significant 1D distance modulation of firing rate, we looked for bins along the interbat distance axis, which exhibited significantly high or significantly low firing rate compared with both types of shuffles. This was done as follows: we first smoothed with a three-bin rectangular window the real distance tuning curve and all of the shuffled tuning curves (this smoothing was performed to avoid detection of transient increase or decrease in firing rate). We next computed the percentile of each bin from the real distance tuning curve compared with the shuffles’ values for the same bin. To correct for multiple comparisons for the number of bins, we computed the average response width, and required the following corrected critical-value: $\frac{\alpha }{{N}_{{\rm{total}}{\rm{bins}}}}\times {N}_{{\rm{bins}}{\rm{in}}{\rm{width}}}$, where: *α* = 5% (the standard critical-value significance criterion, before correction (95% shuffling)); *N*_total bins_ = 40 (total number of 2 m bins of distance within the ±40 m distance range); and *N*_bins in width_ = 4 (average response width = 4 bins = 8 m). This resulted in *α*_corrected_ = 0.5% (two-sided), that is, we considered the firing rate in this bin to be significant if it was above the 99.5% percentile or below the 0.5% percentile of the shuffles. To prevent edge effects, we removed significant bins from the edges of the ±40 m range (that is, if the first or last bin were significant). Moreover, in each of the shuffles, we considered bins to be significant only if they had at least one additional significant neighbouring bin (that is, we required ≥2 adjacent significant bins; single significant bins were discarded). Finally, we considered a bin to be significant only if it was significant at the 99.5% level according to both types of shuffles (cross-over shuffle, and solo projected on cross-over shuffle). This requirement to be significant according to two fundamentally different shuffling procedures ensured that only robust distance tuning would be detected.

#### Defining 1D distance cells

1D distance cells (cells with significant 1D distance tuning) were defined by analysing the 1D distance tuning, using the following criteria: (1) the 1D tuning had significant distance bins above or below both types of shuffles (as described above). (2) The cell emitted ≥30 spikes during cross-overs in that flight-direction. (3) Stability of the tuning curve: Pearson correlation of *r* ≥ 0.3 between distance tuning curves computed in even flights and odd flights. We note that most of the cells with significant 1D distance tuning were not purely tuned only to distance, but rather had complex 2D tuning to distance by position (Figs. 3a,g and 5).

### Control for movement-related responses

During cross-overs, bats tended to slightly lower their speed and slightly deviate laterally to avoid colliding with each other (Extended Data Fig. 6b,c (speed and velocity *Y*, respectively)). To test whether the distance modulation of the neurons is genuine and is not due to neuronal responses to these movements, we reasoned that we can compute the tuning to these movement variables during solo flights to disentangle it from the cross-over effects: if a cell is modulated by a movement variable, we expect to see the same movement-related modulation both during solo and during cross-over flights. We considered three movement-related variables that could potentially modulate the firing rate of the cells—we focused on speed and velocity because previous studies have shown that some hippocampal neurons are sensitive to movement speed or to manoeuvring^44,46^: (1) speed: $S=\sqrt{({{V}_{X}}^{2}+{{V}_{Y}}^{2})}$ where *V*_*X*_ is the velocity along the long axis of the tunnel, *X*, and *V*_*Y*_ is the velocity along the lateral axis of the tunnel, *Y* (see Extended Data Fig. 6a for an illustration of the *X,Y* axes); (2) *V*_*Y*_, velocity in the Y axis, reflecting deviation (manoeuvring) in the lateral axis of the tunnel; and (3) *S*_*Y*_, speed in the *Y* axis (absolute *Y* velocity, irrespective of the direction of the deviation). The positional resolution along the *Z* axis was not high enough to analyse behavioural modulations in *Z*, but observations during the experiment indicated that the deviations in *Z* were small.

For each movement variable, we built solo tuning curves and cross-over tuning curves (Extended Data Fig. 6d–f). To compare between solo and cross-over, we limited the data from which we built both tuning curves to include the same range of flight speeds in both cases; the range was set to 5–95% of the flight speeds during cross-overs, computed separately for each bat. We computed the tuning curves as described above (see the ‘1D distance tuning and classification of 1D distance cells’ section), using bin sizes of 0.2 m s^−1^ for velocity *Y* and speed and 0.1 m s^−1^ for speed *Y*. To evaluate the significance, we used 10,000 circular shuffles of the spikes as follows: we concatenated all of the in-flight spikes across all of the flights (separately for solo and cross-over flights), and we then rigidly shifted the times of all of the spikes by a random time interval in a circular manner (with the end of the session wrapped to the beginning). For each shuffle, we computed velocity tuning and speed tuning as for the real data. We then fitted a linear function to these tuning curves for both the solo and cross-over data, as well as for their shuffles, as velocity and speed modulations were found to be approximately linear in the hippocampal formation^47,48^. We defined cells as significantly modulated by velocity or speed if the slope of the linear fit was >97.5% percentile or <2.5% percentile of those seen in the shuffles (with significance assessed separately for the solo and for the cross-over conditions; Extended Data Fig. 6g–i). In total, we performed 6 linear fits = 3 movement variables (*S*, *V*_*Y*_, *S*_*Y*_) × 2 behavioural modes (solo, cross-overs). We considered a cell to be movement-modulated by one of the variables if it was significantly modulated by this movement variable both in solo and in cross-over, and had the same slope direction of the tuning during both solo and cross-over. The results of these control analyses are shown and further elaborated in Extended Data Fig. 6 – which ruled out any major contribution by velocity or speed.

### Control for sensory or motor responses to the echolocation clicks

To exclude the possibility that the observed distance tuning during cross-over is directly linked to the echolocation clicks—that is, reflects a direct sensory neuronal response to the clicks themselves (auditory responses) or a motor neuronal activity before individual clicks—we used the solo flights as a control. As during the solo flights bats also emit echolocation clicks (albeit at a lower rate; Fig. 1e (black error bar) and Extended Data Fig. 2b), we tested whether the neurons show any auditory/motor response to the clicks when appearing outside of the context of cross-overs, and examined whether such responses could explain the distance tuning observed during cross-overs. To this end, we computed click-triggered responses during solo flights at ±200 ms around each click, using 10 ms bins, and averaged the responses across clicks (Extended Data Fig. 7a (bottom, black lines)). We compared each click-triggered response to 10,000 shuffles (rigid circular shuffling of spikes within the ±200 ms time window around each click, similar to the shuffling described above for place tuning), and considered a bin to be significant if it was above the 99.5% percentile or below the 0.5% percentile of the shuffles (corrected for multiple comparisons as described above, assuming 4 bins = 40 ms for the minimal duration of sensory/motor response). To exclude transient and unreliable responses, we considered only modulations that were significant in two or more consecutive bins. This analysis was performed on all of the 1D distance cells that were recorded simultaneously with audio (*n* *=* 41 cells × directions). The results of these control analyses are shown in Extended Data Fig. 7a–d and they strongly argue against the possibility that the 1D distance tuning could be explained by sensory or motor responses to clicks.

### Modulation of the 1D distance tuning curve by attention

To test how attention modulates the distance tuning curves and firing rates of the neurons during cross-overs, we used the increase in echolocation click rate as an index of attention^1–9,38–41^. For 1D distance cells that were recorded simultaneously with audio (which was performed in one bat), we divided the cross-over flights into two equally sized groups according to the click rate (median bisection). This yielded two sets of flights: flights with high click rate versus flights with low click rate (reflecting high versus low attention). We then computed the tuning curves separately for these two sets of flights (as done above for the full 1D tuning curves). The separation to flights with high and low click rates was based on the mean click rate in a distance window between −15 to 0 m (this distance window matches the typical region in which the click rate was increased during cross-overs; Fig. 1e and Extended Data Fig. 2c). Click-rate tuning curves were computed for each individual cross-over flight (using a bin size of 2 m and smoothing by a Gaussian kernel with *σ* = 1.5 bins). We included in this analysis only valid flights that met the following criteria: (1) the flight had enough continuous distance data (from −25 to 5 m with no gaps; 4.4% of the flights were excluded); (2) the peak click rate was greater than 10 Hz—this threshold was used to exclude rare atypical cases of very sparse use of echolocation (on average 2.56 flights per direction per session were excluded, 5.6% of the flights in total); and (3) more than twofold change in sonar click rate during cross-over relative to the preceding minimum—here we removed atypical flights with almost no modulation of sonar click rate along the cross-over flight (this excluded 2.8% of the flights).

After applying these criteria for valid flights, we included in this analysis only cells that had 20 or more valid cross-over flights, and 30 or more spikes in high-attention trials or low-attention trials (similar to the requirement of 30 spikes in the main analysis of 1D distance tuning). This left for analysis *n* = 37 cells × directions out of the *n* *=* 41 total 1D distance cells that were recorded simultaneously with audio.

To examine the difference between the tuning curves in high-attention versus low-attention flights, we focused on a window of ±10 m around the peak firing rate of each cell (peak firing rate of the 1D distance tuning curve using all cross-over flights), and computed within this window two quantities: (1) the rate difference of the peak firing rates between the high-attention tuning curve and the low-attention tuning-curve, and (2) the rate difference of the mean firing rates between the two tuning curves. For significance, we divided randomly 10,000 times the cross-over flights into two groups, and computed the same two quantities, resulting in two shuffle distributions (Extended Data Fig. 7e (bottom row)). Cells that were higher than the 95th percentile in one of these shuffle distributions were defined as significantly attention modulated (*n* = 11 cells × directions, comprising 29.7% of the cells in this analysis; see Fig. 2e and Extended Data Fig. 7e for examples and Fig. 2f,g for the population).

### Testing for position representation of the other bat

To test whether during cross-overs there is representation of the position of the other bat in world coordinates (that is, representation of the other bat’s allocentric position)—as opposed to representation of the distance from the other bat—we computed the corresponding 1D tuning curve: the firing rate of the neuron as a function of the position of the other bat (using the same procedure as described above for the tuning curve for self position). We defined a cell to be significantly tuned for position of the other bat if it passed the following criteria: (1) criteria identical to those used for determining position tuning for self place cells: (i) significant spatial information (99% percentile) compared to shuffles; (ii) spatial information was >0.25 bits per spike. (2) Criteria identical to those used for determining distance tuning during cross-over, as here the analysis was done during cross-over flights: (i) the cell emitted ≥30 spikes during cross-over flights; (ii) stability of the tuning-curve over even and odd flights: Pearson correlation of *r* ≥ 0.3 between these tuning curves. The results of these control analyses are shown and further elaborated in Extended Data Fig. 8, ruling out the possibility that, in this particular experiment, the bat’s hippocampal CA1 cells represented the position of the other bat; rather, the neurons represented the distance to it.

### Distance tuning within place fields and between place fields (within interfields)

Computation of the 1D distance tuning curves within place fields (Fig. 3a and Extended Data Fig. 11a) was performed on the cross-over data, using only segments of behavioural data and neuronal data that occurred within the place field (the place field was defined during solo-flights, as described above). For computing the 1D distance tuning curves in interfield areas (between place fields), the interfield area was defined as follows: after we expanded each place field by 50% of its width to both sides (thus adding extra margins), the remaining areas between these expansions were defined as interfields. These extra margins were removed to prevent leakage of spikes from the adjacent place fields into the interfield. We then computed the 1D distance tuning curves for data within fields/interfields in the same way as it was computed for the entire cross-over data (described above), with small modifications to account for the smaller amount of data available within fields/interfields: (1) we used larger distance bins (2.6667 m; 30 bins); and (2) we discarded bins with less than 0.1 s time spent. To include place fields and interfields with good coverage and reliable spiking, we analysed only tuning curves with at least 30 spikes (as in the 1D tuning curve) and for which at least 80% of the distance bins were valid (above the minimal time spent of 0.1 s). Note that most interfields had, by definition, a small number of spikes, and their tuning could therefore not be analysed reliably (*n* = 87 interfields were valid for analysis). The resulting 1D tuning curves were based on 20.87 ± 6.55 flights per place field and 29.41 ± 12.38 flights per interfield (mean ± s.d., across all place fields or interfields).

#### Significant bins

To identify significant bins in these tuning curves (within fields/interfields), we performed a similar analysis as described above for the full cross-over data, but with slight modifications to account for the smaller amount of data. After finding the significant bins in both types of shuffles as described above (cross-over rigid shuffles and solo projected on cross-over shuffles), we removed significant bins at the edges of the distance range. We then considered a bin to be significant if it was significant in one type of shuffle (above the 99.5% percentile or below the 0.5% percentile of the shuffles, that is, corrected for multiple comparisons as described above), and was also above the 95% percentile or below the 5% percentile of the other type of shuffle. We required that the number of flights that contributed to a significant distance field (the consecutive set of significant bins) was at least two flights. The number of flights differed between narrow and wide fields; across the population, the mean number of flights per distance field was 8.13 ± 4.12 (mean ± s.d.).

#### Switch time

To quantify how rapidly place cells can change their representation from representing position to representing distance by position (Fig. 3c and Extended Data Fig. 11e), we analysed switch times for individual place fields. We first recomputed the tuning curve within the place field in terms of time to cross-over rather than interbat distance (using the same number of bins). As time to cross-over and interbat distance are highly correlated (Extended Data Fig. 4d,e; Pearson *r* *=* 0.9987 ± 0.0009 (mean ± s.d.)), the tuning curves were highly similar to each other. We next created a shuffle distribution using the cross-over rigid shuffling (as above), and computed the median of these shuffles per time bin. We upsampled (×10) both the real data and the median shuffle tuning curves to get finer temporal resolution. We next computed the time difference between the time point at which the real data crossed the median of the shuffle, and the time point of the first significant bin, and defined this time difference as the rise time for enhancement and fall time for suppression (Fig. 3c (dark green in the top plot and dark brown in the bottom plot)). Similarly, we defined the time difference between the time point of the last significant bin and the time point at which the data subsequently crossed the median of the shuffle as the fall time for enhancement and rise time for suppression (Fig. 3c (light green at the top and light brown at the bottom; see the arrows)). We computed these switch times only for tuning-curves with one significant enhancement or one significant suppression (not both), and only when the modulation crossed the median shuffle on both sides, resulting in *n* = 204 fields (out of a total of *n* = 303 significant fields), of which *n* = 143 were enhancement tuning curves and *n* = 61 were suppression tuning curves.

### Distance-tuning correlations between place fields

In Fig. 5b,c (correlations of distance tunings between place fields), we excluded pairs of tuning curves if both place fields did not have significant distance modulations to avoid correlating small random noises, or if the position gap between the place fields was smaller than half their average width to avoid ‘leaking’ of spikes from one field to the other.

### 2D firing rate maps for distance by position, and patch analysis

#### Firing rate maps

2D firing-rate maps for interbat distance by position were computed by counting spikes and time spent in each 2D bin (bin size: 3 × 3 m), which resulted in two 2D maps—a spike-count map and a time-spent map. Bins with time spent <0.2 s were discarded from the analysis, unless an adjacent bin was visited. We smoothed the spike map and the time-spent map with a 2D Gaussian kernel (*σ* = 1.5 bins = 4.5 m). The 2D firing rate map was then computed by dividing bin by bin the smoothed spike-count map by the smoothed time-spent map. Note that identical binning and smoothing were also used for plotting 2D distance-by-position maps of click rate and velocity (Fig. 1f and Extended Data Figs. 2d and 6b,c).

#### Patch analysis

To find regions in the 2D firing-rate maps for distance by position that were significantly modulated (significant 2D patches; Fig. 3f and Extended Data Fig. 12e,f), we used cluster-based analysis^49,50^. This method, which is widely used for analysing data from functional magnetic resonance imaging and electroencephalography experiments, searches for contiguous groups of significantly modulated bins or pixels. First, we compared the firing rates in each bin of the real 2D map with the firing rates in the same bin in all 10,000 shuffle maps (using solo projected on cross-over shuffling; see Extended Data Fig. 5c for examples of shuffle maps), and computed the *P* value for each bin on the basis of its percentile compared with all the shuffle maps. Here we used the solo projected on cross-over shuffle because it accounts for the prominent position coding in the hippocampus, and it reflects the null hypothesis of how a neuron would have responded if it was only place-tuned and was not modulated by the interbat distance (see above for the description of this shuffling; Extended Data Fig. 5a,c). Second, neighbouring bins that passed a significance threshold of *P* > 0.995 were clustered together as enhancement clusters, and neighbouring bins that passed a threshold of *P* < 0.005 were clustered together as suppression clusters. For each cluster, we computed the surprise values for each bin (surprise was defined as –log_10_[*P*] for suppression clusters and –log_10_[1 − *P*] for enhancement clusters). We then summed the surprise values over all of the bins in that cluster to get the cluster score. We did this entire procedure (first and second step) both for the data 2D map and for all 10,000 shuffle 2D maps (treating each shuffle map as if it was a data map). Third and finally, we then compared the cluster scores in the data 2D map with the distribution of the highest cluster score in each shuffle 2D map (separately for enhancement and suppression). We considered clusters that were ranked above the 95% percentile compared with the shuffle as significant 2D patches. We then considered only 2D patches that passed additional two criteria, both of which were aimed to accept only patches that were based on enough behavioural-data: (1) at least 2 s of total flight time inside the 2D patch; and (2) on average at least 0.15 s of flight time for each bin in the patch (3 × 3 m bins). Moreover, we used three spike-based criteria for enhancement patches only (not for suppression patches)—these criteria were aimed to accept only enhancement patches that contained enough spikes: (1) at least 20 spikes inside the 2D patch, (2) average of at least 0.5 spikes per bin, and (3) at least 3 flights with spikes inside the 2D patch. We performed this analysis on all of the pyramidal cells that had a peak firing rate of ≥2 Hz in their 2D map (*n* = 607 cells × directions). Applying all the above criteria enabled us to conduct the analysis only on cells with reliable behavioural coverage and robust firing rates. Note that the patches were generally quite localized along the distance axis (the median distance patch width was 24.6 m for enhancement patches and 18.5 m for suppression patches, with the median taken over the maximum distance width for each patch; the median over the average width for each patch was 16.9 m for enhancement patches and 12.3 m for suppression patches).

#### Defining 2D distance-by-position cells

A cell was defined as a significant 2D distance-by-position cell if either: (1) it had significant modulation of firing rate within a place field or interfield (see above; Fig. 3a,d and Extended Data Figs. 11 and 12a–d); or (2) it had a significant patch within the 2D distance by position map (see above; Fig. 3f and Extended Data Fig. 12e,f). These were the *n* = 366 cells × directions that were marked as cells with 2D distance by position tuning in Fig. 3g (purple ellipse).

### Decoding analysis

We simultaneously decoded the interbat distance and the position of the bat during cross-overs (Fig. 4e–h and Extended Data Fig. 13) using a Bayesian maximum-likelihood decoder^11,51^. Decoding was performed for each flight direction separately. We performed leave-one-out cross-validation, in which each cross-over flight served as test data, while the remaining flights were used to build the 2D firing-rate map for each cell (train data). We analysed only sessions with ≥10 simultaneously recorded cells (16 sessions; *n* = 13.19 ± 3.08 cells per session (mean ± s.d.); for the decoding analysis, we included all cells without any exclusion criteria).

The decoded position and interbat distance at every moment were those that maximized the log-likelihood function, assuming Poisson firing, that is, maximized:

$$A({x}_{D},{x}_{P}| \{{{\bf{n}}}_{i}\})=\mathop{\sum }\limits_{i=1}^{N}{n}_{i}\log [\tau {f}_{DP,i}({x}_{D},{x}_{P})]-\tau \mathop{\sum }\limits_{i=1}^{N}{f}_{DP,i}({x}_{D},{x}_{P})+\log [P({x}_{D},{x}_{P})]$$

The term on the left denotes the log-likelihood of the bat being located in a specific combination of (distance, position) = (*x*_*D*_*,* *x*_*P*_) given the observation of a vector of {**n**_*i*_} spikes in each of the *N* neurons. On the right, the first term corresponds to a sum of the log of the spatial tunings of all neurons (that is, ${f}_{DP,i}({x}_{D},{x}_{P})$), weighted by their activity (*n*_*i*_) in the integration window *τ*, that is, the time bin used for decoding (we used *τ* = 1 s, with a 250 ms overlap: a relatively long time window, which was used due to the relatively small number of neurons). The second term corresponds to a correction for unequal coverage of the neuronal representation in different locations. The third term on the right side corresponds to the prior for the decoding (*P*(*x*_*D*_,*x*_*P*_)), which is needed due to the non-perfectly-uniform behaviour (time spent in the 2D distance by position space). The decoded position and distance coordinates were then taken as the combination that maximizes *A*(*x*_*D*_,*x*_*P*_|{**n**_*i*_}), that is, the maximum of the log-likelihood function.

In Fig. 4c, we used the same type of decoding, but instead of using the 2D firing-rate map we used the 1D position tuning computed based on the solo flights to decode the position during cross-over flights (Fig. 4c (pink)). Chance error was estimated by computing the median difference between the actual (real) positions and 100 random positions, which were sampled from the experimentally measured behaviour of the animal, to account for non-homogeneous behaviour (Fig. 4c (grey)).

In Fig. 4a–d, we used a 10 m distance sliding-window (2 m steps), and computed position tuning curves during cross-over flights separately for each distance window. These were then used to compute correlations with solo position tuning (Fig. 4a), spatial information (Fig. 4b) and mean normalized firing rate (Fig. 4d).

### SVD analysis

To quantify the non-separability of the 2D distance-by-position coding, we performed a singular value decomposition (SVD) analysis on the 2D distance by position firing-rate map, denoted here by the matrix *M*. This is a standard analysis performed in neuroscience to assess the separability of 2D coding^31–33,36,52^. In this analysis, we decompose the matrix *M* (throughout this analysis, the matrix *M* was always mean-subtracted before the SVD decomposition):

$$M=U\times S\times {V}^{T}$$

Here *U* and *V* are matrices containing the singular vectors corresponding to the position and distance axes, respectively, and *S* is a diagonal matrix with non-negative singular values (*s*_*j*_ in descending order); the superscript *T* denotes matrix transpose. This analysis enabled us to test whether the matrix *M*, namely the 2D distance by position firing-rate map, is completely separable, which means that it equals the first column vector in *U* multiplied by the first column vector in *V* × *s*_1_ (see Extended Data Fig. 14a (cell 117), where the first dimension, that is, the multiplication of the first two vectors, is almost identical to the original matrix); otherwise the matrix *M* is non-separable and requires additional terms, each being a product of vectors from *U* and *V* scaled by the corresponding *s*_*j*_ (Extended Data Fig. 14a (cells 325 and 235)). We computed two types of measures to quantify the non-separability, based on the singular values. These measures, *λ* and *α*, were: (1) *λ* is the exponential decay fitted to the singular values: ${s}_{j}=a\times {e}^{\frac{-j}{\lambda }}$. For separable cells, the decay is fast, that is, *λ* is small, whereas, for non-separable cells, *λ* is large. (2) $\alpha =1-\frac{{s}_{1}^{2}}{\mathop{\sum }\limits_{j=1}^{n}{s}_{j}^{2}}$: the fraction of variation in *M* that is not captured by the first singular value *s*_1_. For separable cells the first singular value is large relative to the subsequent singular values, and therefore *α* is close to zero; by contrast, non-separable cells have larger values of *α* (ref. ^31^). Note that these two measures, *λ* and *α*, are highly correlated among themselves (Pearson *r* = 0.98, *P* = 7.1 × 10^–172^, *n* *=* 262 cells × directions).

As SVD analysis requires a rectangular matrix *M*, and our 2D firing-rate maps were non-rectangular (see the 2D distance by position maps in Fig. 2a), we cropped our 2D map along the position axis (*y*) such that in each row we will have at least 80% valid distance bins (80% of the ±40 m range). We computed the 2D firing rate map again using only spikes and behavioural data that occurred within this cropped position area (this cropping is the reason for the smaller *y*-range (positions) of the maps in Fig. 5e as compared to Fig. 2a). To create a full rectangular map as SVD requires (that is, to fill non-visited bins in the 2D map) we used the following iterative procedure to estimate the matrix entries while not adding any excess non-separability: (1) we initially filled-in the empty bins on the basis of the local mean firing rate of the neighbouring bins; this created a full matrix, *M*_first iteration_. (2) We next computed the SVD for this new full matrix *M*_first iteration_, and changed the values of the original empty bins to values computed from the first 10 singular values and vectors, resulting in a new matrix, *M*_second iteration_ (using a large number of 10 singular values ensured that this estimation procedure would not restrict the map dimensionality). We then computed the sum of squared differences between the current matrix *M*_second iteration_ and matrix *M*_first iteration_. (3) We iteratively continued to change the values of these bins by computing the SVD and building a new matrix *M*_*i*_, and then computing the sum-of-squared-differences between the current matrix *M*_*i*_ and the previous matrix *M*_*i* − 1_, until reaching convergence (sum of squared difference smaller than 0.001). The final matrix used in this analysis was referred to as *M*_SVD_, which is rectangular and has no empty bins.

We conducted SVD analysis only on pyramidal cells with significant distance modulation either in 1D or 2D that met the following criteria. Criteria on behavioural coverage: (1) the length of the position axis of the cropped rectangle was ≥45 m. (2) The largest position gap between adjacent cross-over flights was <10 m (that is, there were no large ‘holes’ in the behavioural coverage). (3) The number of cross-overs in the cropped map was >10. Criteria on spiking: (4) the number of spikes in the cropped map was >30. (5) The peak firing rate in the cropped 2D map was >2 Hz. Applying these criteria enabled us to conduct the analysis only on cells with reliable behavioural coverage and robust firing rates (*n* = 262 cells × directions).

To assess the significance of *λ* and *α*, we used as shuffles our 2D matrices from the solo projected on cross-over shuffle (see above; and see examples in Extended Data Fig. 5c). These maps were cropped and filled in the same way as the data map, and as by definition they exhibit only position tuning, we multiplied each row of the 2D matrix by the 1D distance tuning of the cell (Extended Data Fig. 14c (bottom panel in each shuffle); multiplied map). This results in matrices with the same behavioural data, the same spike statistics and the same 1D distance tuning as in the real data, but these ‘multiplied maps’ are almost separable, and any non-separability that we would measure in them must therefore arise from non-uniform coverage of bat behaviour or from noisy spiking. Thus, by comparing the 2D data maps to these shuffles, we can test whether the detected non-separability in the cell’s 2D data map is genuine, or whether it originates from noisy spiking or from non-homogenous behaviour. Cells were defined as significantly non-separable cells if both their *λ* and *α* were above the 95% percentile of the shuffle distribution, and their rounded projection dimension was ≥1 in the cross-validation test (see below).

#### Cross-validated SVD

We also performed a cross-validated SVD analysis to assess the dimensionality of the matrix^53^ (Extended Data Fig. 14d). Here we divided the bins in the matrix *M*_SVD_ into train bins (90%) and test bins (10%, which were randomly picked for each of the 1,000 iterations of the cross-validation). We first set the values of the 10% test bins to the average value of the entire matrix *M*_SVD_. We then used SVD to reconstruct this matrix (90% real data, 10% set to the mean value) with increasing cumulative dimensions (for example, cumulative dimension 2 = *U*_1_ × *s*_1_ × *V*_1_^*T*^ + *U*_2_ × *s*_2_ × *V*_2_^*T*^; *x* axis in the bottom left panel of each cell in Extended Data Fig. 14d). We iteratively changed the test bins using the same method as described above for filling-in the empty bins, until reaching convergence. We next computed (separately for the test and the train bins) the mean squared error between the values of the original bins and the values in the new reconstructed matrix using different cumulative dimensions. For the training bins, it is guaranteed that the error decreases as we add more dimensions (that is, as we increase the cumulative dimension). However, for the test bins, the error will decrease only if the added dimension indeed describes the data; thus, at some point, the error will start to increase when adding more dimensions because the additional dimensions are over-fitted to the training portion of the data, and therefore effectively add noise. We therefore define the cumulative dimension with the minimal test error as the meaningful dimension of the data (Extended Data Fig. 14d (bottom left panel in each cell): minimum of red curves). Finally, to remove any non-separability that may result from the non-uniform behavioural coverage, we carried out the same procedure also for the median map of the solo projected on cross-over shuffles (Extended Data Fig. 14d (right column for each cell)). Then, to assess the real dimensionality of our data, excluding the behavioural dimensionality, we computed the projection dimension: for each of the 2D matrices—the cross-over data matrix and the solo-median matrix—we took only the meaningful vectors from *U* and *V* (all of the vectors until and including the meaningful dimension); and projected the vectors of the solo median map out of these data vectors. This results in a subspace that is orthogonal to the solo median map space and therefore does not contain the behavioural dimensionality anymore:

$${U}_{p}={U}_{{\rm{data}}}-\left({U}_{{\rm{solo}}}\times {U}_{{\rm{solo}}}^{T}\right)\times {U}_{{\rm{data}}}$$

$${V}_{p}={V}_{{\rm{data}}}-\left({V}_{{\rm{solo}}}\times {V}_{{\rm{solo}}}^{T}\right)\times {V}_{{\rm{data}}}$$

where *U*_data_ and *V*_data_ are matrices containing the meaningful vectors of the data, and *U*_solo_ and *V*_solo_ are matrices containing the meaningful vectors of the solo median map. Then, the projection dimension of the data excluding the behavioural dimensionality was computed as the minimum of the sum of the normalized projected vectors:

$${\rm{projection}}\,{\rm{dimension}}=\min \left(\mathop{\sum }\limits_{i=1}^{N}{U}_{{p}_{i}}^{2},\mathop{\sum }\limits_{i=1}^{N}{V}_{{p}_{i}}^{2}\right)$$

### Model of 2D distance by position maps, and decoding the model’s simulations

To assess the possible functional role of the observed non-separable maps (Figs. 3a and 5), we created populations of neurons with simulated distance by position maps, and systematically studied the effect of map separability on decoding performance. All of the simulations were conducted using MATLAB.

In our simulations, the bin size was 0.5 m both for the distance coordinate (*x*_*D*_, interbat distance; between ±40 m) and for the position coordinate (*x*_*P*_, position in the tunnel; between 0–130 m; we simulated the 130 m that were effectively covered by the bats). We note that below we make repeated use of the gamma distribution, defined by a shape parameter *k* and a scale parameter *θ*, to fit the model to the empirical data. The 2D distance by position map of neuron *i* is denoted below by *f*_*DP,i*_(*x*_*D*_*,* *x*_*P*_).

#### Position encoding

Position tunings were generated using a similar procedure as in our previous work^14^, with slight modifications of the parameters to fit the current experimental dataset. In brief, for each model neuron, we randomly picked a position coverage value from a gamma distribution that was fit to the data (*k*_coverage_ = 1.76, *θ*_coverage_ = 0.18); if this sample value was larger than 0.8, we resampled. Next, we sampled field sizes from a gamma distribution fitted to the data (*k*_field-size_ = 4.62, *θ*_field-size_ = 2.68 m), adding fields until the cumulative (total) sizes of all fields reached the coverage value. This number of place fields is denoted *n*_field_. Then, the field locations were randomly and uniformly distributed along the environment, with no overlaps. To avoid distorting the uniform distribution of fields near the boundaries of the environment, we allowed fields to be located anywhere and we truncated them at the boundaries. This procedure created cells with multiple place fields, where the sizes of the fields of the same neuron were broadly distributed, as seen in the experimental data.

#### Distance modulation

Distance modulation profiles were generated as follows. For each neuron, the number of distinct distance profiles was set to *n*_dis_ = *n*_field_ × *x*_sep_ (rounded up, where *x*_sep_ is a parameter controlling the degree of non-separability). We varied *x*_sep_ between 0.2 and 1 in jumps of 0.2 (Extended Data Fig. 15a (columns)). Each field was assigned with one of the *n*_dis_ modulation profiles. When *n*_dis_ = 1, all fields undergo the same distance modulation (maximal separability), and when *n*_dis_ = *n*_fields_, each field undergoes different distance modulation (maximal non-separability). We generated *n*_dis_ sets of field-modulation values: distance modulation was enhancing/suppressing/not modulated with a probability of 0.38/0.20/0.42, respectively (numbers fitted to the experimental data). Across the population, the average number of fields that underwent distance modulation was independent of *x*_sep_ because the probabilities (0.38/0.20/0.42) were independent of *x*_sep_. The centre location of each distance-modulation profile was sampled from a Gaussian distribution (*µ* = 0 and σ = 16.8 m; fitted to the experimental data), and the profile width was sampled from a uniform distribution between 4.3–12.9 m (the average of that distribution was matched to the experimental data). When a field is enhanced/suppressed, the firing rate in the region modulated by the distance coordinate was multiplied by a factor sampled from a gamma distribution (*k*_enhance_ = 9.98, *θ*_enhance_ = 0.32; *k*_suppress_ = 0.39, *θ*_suppress_ = 0.17; fitted to the experimental data).

We also added 2D distance-by-position modulation irrespective of place fields (Extended Data Fig. 15a (hotspots)), to reflect better the experimental data, where activation hotspots were found in the interfield analysis (Fig. 3d and Extended Data Fig. 12a) and in the patch analysis (Fig. 3f and Extended Data Fig. 12e,f). The distance-by-position firing rate map of each neuron had a hotspot with probability (*x*_sep_ − 0.2)/0.8 (that is, no hotspots when *x*_sep_ = 0.2 and one hotspot for each neuron when *x*_sep_ = 1). The size, position and increase in firing rate of the hotspot was sampled similarly to the distance enhancement modulation profile. The position of the hotspot centre was sampled uniformly.

#### Quantifying the non-separability of maps

The parameter *x*_sep_ enabled us to create 2D distance-by-position maps *f*_*DP*,*i*_(*x*_*D*_, *x*_*P*_) with differing non-separability levels (Extended Data Fig. 15a (columns)). After maps were generated, we computed for each 2D map the non-separability indices of the SVD analysis, *λ* and α, as we did for the experimental maps (Fig. 5e–g; see the definitions of *λ* and *α* in the ‘SVD analysis’ section above).

#### Generating spike counts for decoding analysis

We assumed that the animal starts each iteration (each ‘simulation-trial’) at a position *x*_*P*_ = *x*_0*P*_ in the tunnel (between 0 and 130 m) at a distance *x*_*D*_ = *x*_0D_ from the other bat (between ±40 m). Both animals fly at a speed of *v* = 8 m s^−1^ for a time interval Δ*t* = 500 ms, in opposite directions. The expected spike count of the neuron during that trial is based on the bat’s trajectory through the 2D map *f*_*DP*,*i*_(*x*_*D*_, *x*_*P*_), and is given by:

$${m}_{i}=\frac{{m}_{0}}{\Delta t}{\int }_{0}^{\Delta t}{f}_{DP,i}({x}_{0D}+2vt,{x}_{0P}+vt){\rm{d}}t$$

where *m*_0_ is the in-field expected spike count without distance modulation: we used *m*_0_ = 5 in all our simulations, as in ref. ^14^. The factor 2 in *x*_0*D*_ + 2*vt* reflects the double distance travelled when each of the two bats flies at a speed *v* towards the other. The actual spike count in each trial was drawn from a Poisson distribution with rate *m*_*i*_, and is denoted below as *n*_*i*_.

#### Decoding

We employed two commonly used types of decoders: a maximum-likelihood decoder and a population vector decoder.

For the maximum likelihood (ML) decoder, we computed the log-likelihood for each neuron, and summed over the *N* neurons^11,14,51^:

$${A}_{{\rm{ML}}}\left({x}_{D},{x}_{P}| \left\{{n}_{i}\right\}\right)=\mathop{\sum }\limits_{i=1}^{N}{n}_{i}\log [{m}_{0}\,{f}_{DP,i}({x}_{D},{x}_{P})]-{m}_{0}\mathop{\sum }\limits_{i=1}^{N}{f}_{DP,i}({x}_{D},{x}_{P})$$

The term on the left side denotes the log-likelihood of the simulated bat being located in a specific combination of (distance, position) = (*x*_*D*_, *x*_*P*_) given the observation of a set of {n_*i*_} spikes in each of the neurons *i*. On the right side, the first term corresponds to a sum of the log of the spatial tunings of all neurons, weighted by their activity; the second term corresponds to a correction for unequal coverage of the neuronal representation in different locations. This equation is very similar to the equation used for decoding the data, with the main difference being that here we created a uniform coverage and therefore did not include a term for the prior. This is an approximation of the likelihood function, where the decoder knows each neuron’s firing rate map (that is, *f*_*DP*__,__*i*_(*x*_*D*_, *x*_*P*_)), but it does not rely on continuously computing a convolution of the firing rate map with the animal’s motion and the other animal’s motion. The decoded position and distance coordinates were then taken as the combination that maximizes *A*_ML_(*x*_*D*_, *x*_*P*_|{n_*i*_}), that is, the maximum of the log-likelihood function.

We considered population sizes *N* between 20 and 200 neurons, in jumps of 20. For each value of *x*_sep_ and *N*, we generated 250 random populations. For each population, decoding was done in 41 equally spaced distances (between ±40 m) and 11 equally spaced positions (between 0 and 130 m), that is, 451 combinations of distance by position; we used a larger number of distances as in this study we focused on the distance modulation. For each distance-by-position combination, spike counts were randomly generated five times. Decoding errors were computed after grouping the data in three different ways: (1) based on the non-separability parameter used to generate the maps, *x*_sep_ (Extended Data Fig. 15d,e); (2) based on the *λ* value of the SVD analysis computed post hoc for each population (Fig. 5h,i); and (3) based on the *α* value of the SVD analysis computed post hoc (Extended Data Fig. 15b,c). All three groupings yielded similar results and similar conclusions from the decoder simulations.

Population vector (PV) decoder: the classical PV decoder^54,55^ was adapted to the case in which the stimulus space (that is, the environment) is not circular, and in which neurons can represent more than one location and distance. In each trial, we computed the following sum over the *N* neurons:

$${A}_{{\rm{PV}}}\left({x}_{D},{x}_{P}| \left\{{{\rm{n}}}_{i}\right\}\right)=\mathop{\sum }\limits_{i=1}^{N}{n}_{i}\log [{m}_{0}\,{f}_{DP,i}({x}_{D},{x}_{P})]$$

The decoded location was then taken as the one that maximizes ${A}_{{\rm{PV}}}({x}_{D},{x}_{P}| \{{{\rm{n}}}_{i}\})$.

### Additional discussion

Our results are distinct from previous reports. (1) Two studies tested how brief switches in the environment affect hippocampal activity^56,57^: these studies used non-ethological manipulations such as teleportation^56^ or rotating-platform^57^, whereas here we focused on natural behavioural switches. Moreover, these two studies^56,57^ reported switching between two position maps, whereas here we found switching from position representation to distance-by-position representation. Furthermore, both studies found that different place cells encode the two different position maps at different time points, whereas in our data, the same cells encoded conjunctively distance by position at the same time. (2) Our results are also different from studies that examined hippocampal spatial representation in response to objects that were moved within the arena between trials, but remained stationary within a trial and, therefore, did not evoke a behavioural switch^15,16^. They found neurons that exhibited spatial tuning either with respect to the room or to object coordinates, but not to both conjunctively, whereas, in our data, the same cells encoded conjunctively distance by position at the same time. (3) Our results are fundamentally different from classical remapping studies^58,59^, in which the hippocampus exhibits different position maps when the animal explores different environments in two different sessions. In such studies, there is no behavioural switch at all, because it typically takes a very long time (timescales of minutes) to transfer the animal between the different sessions and, therefore, such studies are not designed to examine rapid behavioural and neuronal switches, as we did here (with timescales as fast as ~100 ms). (4) Our results are also different from our previous findings of encoding of distance to a stationary goal^19^, as here we focused on representation of a moving conspecific, which required a very rapid switch of attention, in contrast to the previous study. (5) Finally, our results also differ fundamentally from our previous study, which found CA1 neurons that represent the position of another bat when the recorded bat is stationary^60^, as here we did not find CA1 neurons that encode the position of the other bat (Extended Data Fig. 8). This difference may stem from task requirements—in the previous study^60^, it was behaviourally important to represent the other bat’s position, whereas here it was important to represent the interbat distance in the context of collision avoidance; we suggest that these major differences in behavioural demands were reflected in the hippocampal neural codes.

Our results showing a non-separable neural code may reflect the non-separable aspect of natural behaviour in the wild. Navigation behaviours typically depend on the location where they happen, for example, commuting in one location versus foraging in another location^61^. This non-separability of behaviour was demonstrated also for collision-avoidance behaviours, for example, in bats that avoid wind turbines differently based on their location^62^, or in social-foraging bats, which respond differently to their conspecifics at different locations^63^. We speculate that the hippocampal system evolved to support (together with other brain areas) the animal’s ability to perform these challenging location-dependent behaviours. As a consequence, we suggest that the non-separable code that we found in the hippocampus of wild-born bats—a code that we showed is more efficient when considering 2D decoding of position by distance—is particularly suitable to guide such non-separable behaviours in the wild. By contrast, a separable neural code might be more suitable in other brain areas, such as inferior temporal cortex, in which an invariant and separable representation of objects is needed (for example, classifying a cat versus a dog irrespective of position). Indeed, a largely separable neuronal code was demonstrated experimentally in the inferior temporal cortex, and was shown to be beneficial for position-invariant object classification^64,65^.

### Reporting summary

Further information on research design is available in the Nature Research Reporting Summary linked to this article.

## Online content

Any methods, additional references, Nature Research reporting summaries, source data, extended data, supplementary information, acknowledgements, peer review information; details of author contributions and competing interests; and statements of data and code availability are available at 10.1038/s41586-022-05112-2.

### Supplementary information

Reporting Summary

### Source data

Source Data Fig. 1

Source Data Fig. 2

Source Data Fig. 3

Source Data Fig. 4

Source Data Fig. 5

## Figures and Tables

**Extended Data Table 1:**
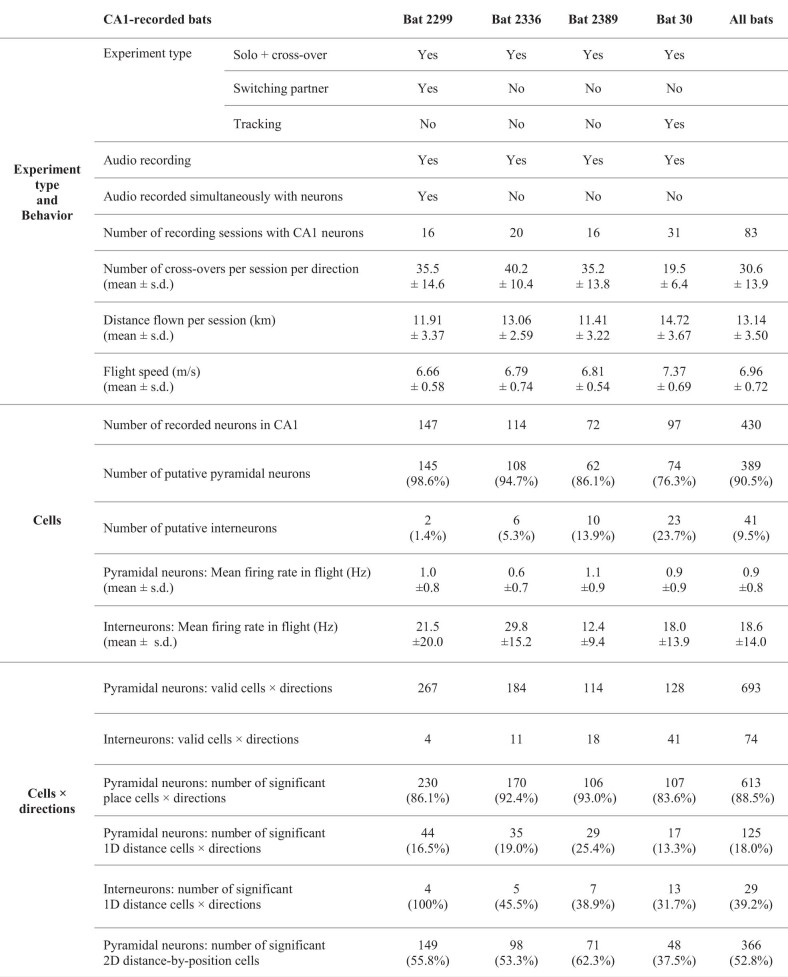
Summary of behaviour and neuronal recordings for all the individual bats

## Data Availability

The data generated and analysed in the current study are available from the corresponding authors on reasonable request. Source data are provided with this paper.

## Code availability

The MATLAB code generated for the current study is available from the corresponding authors on reasonable request.
