# Supplementary material for: Natural switches in behaviour rapidly modulate hippocampal coding
Source: Nature. 2022 Aug 24;609(7925):119–27. doi: 10.1038/s41586-022-05112-2 (PMC9433324; doi:10.1038/s41586-022-05112-2)
Supplement: Supplementary file 1 — Reporting Summary [file 41586_2022_5112_MOESM1_ESM.pdf]

nature portfolio

Corresponding author(s): Nachum Ulanovsky

Last updated by author(s): May 29, 2022

Reporting Summary

Nature Portfolio wishes to improve the reproducibility of the work that we publish. This form provides structure for consistency and transparency in reporting. For further information on Nature Portfolio policies, see our Editorial Policies and the Editorial Policy Checklist.

Statistics

For all statistical analyses, confirm that the following items are present in the figure legend, table legend, main text, or Methods section.

n/a	Confirmed	
☐	☒	The exact sample size (*n*) for each experimental group/condition, given as a discrete number and unit of measurement
☐	☒	A statement on whether measurements were taken from distinct samples or whether the same sample was measured repeatedly
☐	☒	The statistical test(s) used AND whether they are one- or two-sided<br>*Only common tests should be described solely by name; describe more complex techniques in the Methods section.*
☒	☐	A description of all covariates tested
☐	☒	A description of any assumptions or corrections, such as tests of normality and adjustment for multiple comparisons
☐	☒	A full description of the statistical parameters including central tendency (e.g. means) or other basic estimates (e.g. regression coefficient) AND variation (e.g. standard deviation) or associated estimates of uncertainty (e.g. confidence intervals)
☐	☒	For null hypothesis testing, the test statistic (e.g. *F*, *t*, *r*) with confidence intervals, effect sizes, degrees of freedom and *P* value noted<br>*Give P values as exact values whenever suitable.*
☐	☒	For Bayesian analysis, information on the choice of priors and Markov chain Monte Carlo settings
☒	☐	For hierarchical and complex designs, identification of the appropriate level for tests and full reporting of outcomes
☐	☒	Estimates of effect sizes (e.g. Cohen's *d*, Pearson's *r*), indicating how they were calculated

Our web collection on statistics for biologists contains articles on many of the points above.

Software and code

Policy information about availability of computer code

Data collection	Neural data acquisition was done using a wireless neural logger, and echolocation signals were recorded using a wireless audio logger (Deuteron Technologies, SpikeLog-16, SpikeLog-64, AudioLogger). Positional tracking was done using ultra-wideband radio-frequency tags and antennas (BeSpoon Inc). Spike sorting was done using Plexon (version 4.4.2).
Data analysis	We used custom MATLAB code for data analysis (MATLAB version 2019b). The code generated for the current study is available from the corresponding author on reasonable request.

For manuscripts utilizing custom algorithms or software that are central to the research but not yet described in published literature, software must be made available to editors and reviewers. We strongly encourage code deposition in a community repository (e.g. GitHub). See the Nature Portfolio guidelines for submitting code & software for further information.

Data

Policy information about availability of data

All manuscripts must include a data availability statement. This statement should provide the following information, where applicable:

- Accession codes, unique identifiers, or web links for publicly available datasets
- A description of any restrictions on data availability
- For clinical datasets or third party data, please ensure that the statement adheres to our policy

The data generated and analyzed in the current study are available from the corresponding author on reasonable request. Source data are provided with this paper (as excel files).

Field-specific reporting

Please select the one below that is the best fit for your research. If you are not sure, read the appropriate sections before making your selection.

☒ Life sciences ☐ Behavioural & social sciences ☐ Ecological, evolutionary & environmental sciences

For a reference copy of the document with all sections, see nature.com/documents/nr-reporting-summary-flat.pdf

Life sciences study design

All studies must disclose on these points even when the disclosure is negative.

Sample size	We analyzed a dataset of 430 neurons, recorded in dorsal hippocampal area CA1 of 4 bats. The activity of each neuron was recorded for ~2 hours. No power analysis was used to pre-determine the sample size: neither for the number of animals nor for the number of neurons. The numbers of animals and neurons are typical for studies in this research field, in both rodents and bats (e.g. refs. 16, 18, 19, 24, 60 in the paper).
Data exclusions	The inclusion criteria for the cells were based on sufficient behavioral coverage, spike number, and firing stability (see Methods), and are standard for this research field. Exclusion criteria were not pre-determined.
Replication	The effects described were confirmed in multiple neurons recorded over multiple recordings-sessions in 4 animals. The findings reported in this paper were found in each of the 4 individual animals.
Randomization	Not relevant, as there was no randomized treatment of the animals: This study is based on observing the neural responses to the natural behavior of the animals.
Blinding	The investigators were not blinded to the animal identity. Analysis of neural and behavior data was conducted regardless of the identity of the animal from which the data were collected.

Reporting for specific materials, systems and methods

We require information from authors about some types of materials, experimental systems and methods used in many studies. Here, indicate whether each material, system or method listed is relevant to your study. If you are not sure if a list item applies to your research, read the appropriate section before selecting a response.

Materials & experimental systems

n/a	Involved in the study	
☒	☐	Antibodies
☒	☐	Eukaryotic cell lines
☒	☐	Palaeontology and archaeology
☐	☒	Animals and other organisms
☒	☐	Human research participants
☒	☐	Clinical data
☒	☐	Dual use research of concern

Methods

n/a	Involved in the study	
☒	☐	ChIP-seq
☒	☐	Flow cytometry
☒	☐	MRI-based neuroimaging

Animals and other organisms

Policy information about studies involving animals; ARRIVE guidelines recommended for reporting animal research

Laboratory animals	No laboratory animals were used in this study. We used here wild-caught animals.
Wild animals	We studied here Egyptian fruit bats (Rousettus aegyptiacus). Sex: male. Age: adult, 2-10 years of age (note: these long-living bats have a life-span of >20 years). We studied N = 4 bats in which neural recordings were performed, and N = 4 partner bats ("other bats") in which no neural recordings were performed (we performed audio recordings in some of them; Methods). The 8 bats in this study were all wild-born, and were captured as adults in Israel, using butterfly nets. They were transported in a car to the Weizmann Institute, where they were quarantined and then joined a large bat colony at the Institute. After a few months in the lab, we ran the experiments described in this study. Following experiments, the bats were euthanized with Pental for purpose of brain histology.
Field-collected samples	This study did not involve samples collected from the field.
Ethics oversight	The experimental procedures described in this study were approved by the Institutional Animal Care and Use Committee (IACUC) of the Weizmann Institute of Science.

Note that full information on the approval of the study protocol must also be provided in the manuscript.
